# Atmospheric Chemistry of *N*-Methylmethanimine (CH_3_N=CH_2_): A Theoretical and Experimental Study

**DOI:** 10.1021/acs.jpca.2c01925

**Published:** 2022-05-11

**Authors:** Arne Joakim C. Bunkan, Nina G. Reijrink, Tomáš Mikoviny, Markus Müller, Claus J. Nielsen, Liang Zhu, Armin Wisthaler

**Affiliations:** † Section of Environmental Sciences, Department of Chemistry, University of Oslo, P.O. Box 1033-Blindern, 0315 Oslo, Norway; ‡ Institute for Ion Physics and Applied Physics, University of Innsbruck, 6020 Innsbruck, Austria

## Abstract

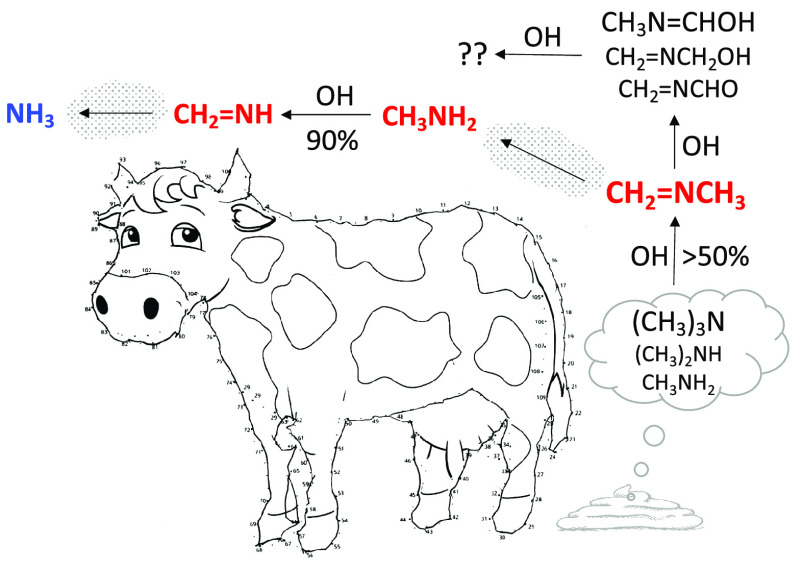

The OH-initiated photo-oxidation of *N*-methylmethanimine, CH_3_N=CH_2_, was investigated in the 200 m^3^ EUPHORE atmospheric simulation chamber and in a 240 L stainless steel photochemical reactor employing time-resolved online FTIR and high-resolution PTR-ToF-MS instrumentation and in theoretical calculations based on quantum chemistry results and master equation modeling of the pivotal reaction steps. The quantum chemistry calculations forecast the OH reaction to primarily proceed via H-abstraction from the =CH_2_ group and π-system C-addition, whereas H-abstraction from the −CH_3_ group is a minor route and forecast that N-addition can be disregarded under atmospheric conditions. Theoretical studies of CH_3_N=CH_2_ photolysis and the CH_3_N=CH_2_ + O_3_ reaction show that these removal processes are too slow to be important in the troposphere. A detailed mechanism for OH-initiated atmospheric degradation of CH_3_N=CH_2_ was obtained as part of the theoretical study. The photo-oxidation experiments, obstructed in part by the CH_3_N=CH_2_ monomer–trimer equilibrium, surface reactions, and particle formation, find CH_2_=NCHO and CH_3_N=CHOH/CH_2_=NCH_2_OH as the major primary products in a ratio 18:82 ± 3 (3σ-limit). Alignment of the theoretical results to the experimental product distribution results in a rate coefficient, showing a minor pressure dependency under tropospheric conditions and that can be parametrized *k*(*T*) = 5.70 × 10^–14^ × (*T*/298 K)^3.18^ × exp(1245 K/*T*) cm^3^ molecule^–1^ s^–1^ with *k*_298_ = 3.7 × 10^–12^ cm^3^ molecule^–1^ s^–1^. The atmospheric fate of CH_3_N=CH_2_ is discussed, and it is concluded that, on a global scale, hydrolysis in the atmospheric aqueous phase to give CH_3_NH_2_ + CH_2_O will constitute a dominant loss process. N_2_O will not be formed in the atmospheric gas phase degradation, and there are no indications of nitrosamines and nitramines formed as primary products.

## 1 Introduction

Imines have been detected as major products in the atmospheric gas phase photo-oxidation of amines,^1−9^ with *N*-methylmethanimine (CH_3_N=CH_2_, MMI) accounting for around 70% of the products formed in dimethylamine and 50% in trimethylamine photo-oxidation.^4^ Amines are normally found in the low ppbv-range in the natural atmosphere, with methylamine, dimethylamine, and trimethylamine being among the most abundant.^10^ Animal husbandry, oceans, and biomass burning are the major sources of methylamines, and cattle are estimated to account for 25% of all methylamine, 33% of all dimethylamine, and 55% of all trimethylamine emissions.^11^ It has recently been established that methylamine and dimethylamine are also among the process degradation products of the more complex amines used in CO_2_ capture,^12^ and they may therefore always be present in the cleaned flue gas, no matter which parent amine that is used in the CO_2_ capture process.

Experimental information on the atmospheric chemistry of imines is scarce; a possible and plausible explanation is that imines are prone to adsorb on surfaces, where they may hydrolyze (>C=NR + H_2_O → >C=O + H_2_NR),^13^ and/or undergo a reversible trimerization reaction to form the corresponding 1,3,5-triazinane.^14^ Tuazon and co-workers^15^ detected MMI as product in the (CH_3_)_2_NH and (CH_3_)_3_N reactions with O_3_ and reported the compound to be essentially nonreactive toward O_3_ contrary to an earlier suggestion that the O_3_ reaction with MMI leads to CH_3_NO_2_ and CH_2_O.^16^ Lazarou and Papagiannakopoulos studied the reaction of MMI with Cl atoms employing the “very low pressure reactor” technique and reported *k*_CH_3_N=CH_2_+Cl_ = (1.9 ± 0.15) × 10^–11^ cm^3^ molecule^–1^ s^–1^ at 303 K,^17^ which is comparable to the low pressure rate coefficient for the CH_3_CH=CH_2_ + Cl reaction (4 × 10^–11^ cm^3^ molecule^–1^ s^–1^ at *p* = 0.44 mbar).^18^ The early study of emission of aliphatic amines from animal husbandry by Schade and Crutzen^11^ includes a speculative atmospheric degradation mechanism for MMI that potentially could lead to N_2_O formation.

There are no previous reliable experimental literature data on products formed in atmospheric imine photo-oxidation; the first MMI photo-oxidation studies were carried out as part of the Norwegian “CO_2_ and Amines Screening Study for Environmental Risks”.^19^ The experiments were hampered by aerosol formation and heterogeneous reactions to the extent that no conclusions were offered.^4^ Recent results from theoretical studies of the OH radical reaction with the simplest imine, CH_2_=NH, imply that this reaction primarily proceeds via H-abstraction with *k*_CH2=NH+OH_ in the range (3–4) × 10^–12^ cm^3^ molecule^–1^ s^–1^ at 298 K^20,21^ and that the major product under atmospheric conditions is HCN.^20^

The present communication reports results from a series of MMI photo-oxidation experiments in the EUPHORE atmospheric simulation chamber, the Oslo stainless steel photochemical reactor, and quantum chemistry based evaluations of the MMI + OH gas phase kinetics and major routes in the OH initiated photo-oxidation of MMI under atmospheric conditions.

## 2 Methods

### 2.1 Experimental Methods and Chemicals

A series of experiments was carried out in chamber B in the EUPHORE facility at CEAM (Valencia, Spain, 39°28′12″N, 00°22′35″W); local time = UTC + 2 during the experiments. The facility and analytical methods have previously been reported in detail;^20^ special online instrumentation employed in the present experiments include a high-resolution PTR-ToF 8000 instrument (*m/*Δ*m* > 3000) from Ionicon Analytik GmbH, interfaced to the EUPHORE chamber via a Sulfinert-passivated stainless-steel tube (length, 125 cm; inner diameter, 5.33 mm; temperature, 75 °C; flow, 11 lpm). A flow of 0.16 lpm was branched off from this main inlet flow into a shortened, 10 cm PEEK inlet capillary. Subsequently, a sample flow of 0.025 lpm was branched off into the PTR-ToF-MS drift tube for analysis (inlet capillary and the drift tube both temperature-controlled at 75 °C). The drift tube was operated at an electric field strength *E*/*N* 88 Td (1 Td = 10^–21^ V m^2^).

In a typical experiment, 1,3,5-trimethyl-1,3,5-triazinane (TMT) was evaporated and flushed into the chamber giving an initial mixing ratio in the range from 50 to 200 ppb. The canopy of the chamber was kept closed for several hours, during which time TMT slowly entered toward equilibrium with MMI, and NO/NO_2_ and an OH-radical precursor were added. The photo-oxidation was followed for around 1 h, after which the chamber was closed and flushed overnight with scrubbed air.

Further MMI and TMT photo-oxidation experiments were carried out in the Oslo 240 L stainless steel Smog Chamber employing FTIR and high-resolution PTR-ToF-MS detection; the system was recently described in detail (in the present experiments the PTR drift tube was operated at 107 Td).^22^ MMI was added to the evacuated chamber by heating a TMT sample to 180 °C and trapping impurities and TMT in two dry ice cold-traps on the fly. TMT and an OH-radical precursor were added to the chamber by injection in a constant stream of replenishment air compensating for the PTR sampling.

Infrared absorption cross sections of TMT were obtained from calibrated spectra obtained of the pure gas at 294 ± 2 K in a cell of 9.85 ± 0.10 cm equipped with CsI windows. The spectra were recorded in the region 4000–400 cm^–1^ using a Bruker IFS 66v FTIR spectrometer equipped with a Ge/KBr beam splitter and employing a nominal resolution of 0.5 cm^–1^. Single channel spectra were recorded averaging 128 interferograms applying Boxcar apodization. To ensure optical linearity, a DTGS detector was used. The pressure in the cells ranged from 1 to 10 mbar and was measured using CERAVAC CTR 100 transmitters with an accuracy of 0.2% of reading (Oerlicon Leybold Vacuum). The absorption spectrum of a 50 ppm·m TMT sample is shown in Figure S1 in the Supporting Information. Figure S2 shows two spectra of MMI/TMT obtained at 80 min intervals and a synthetic spectrum of MMI obtained by spectral subtraction is presented in Figure S3; the figure also includes the vibrational assignment of MMI.^23,24^ It should be noted that the absorption cross sections of MMI are almost an order of magnitude smaller than those of TMT.

1,3,5-Trimethyl-1,3,5-triazinane (Sigma-Aldrich, 97%) and 2-propanol 3,3,3,6,6,6-*d*_6_ (Sigma-Aldrich, 99 atom % D) were used without further purification. *N*-Methylmethanimine was prepared by heating 1,3,5-trimethyl-1,3,5-triazinane to 180 °C and trapping the vapor at liquid nitrogen temperature. 2-Propyl nitrite (isopropyl nitrite, IPN) and 2-propyl nitrite 3,3,3,6,6,6-*d*_6_ (IPN-*d*_6_) were synthesized from sulfuric acid, sodium nitrite, and 2-propanol or 2-propanol-3,3,3,6,6,6-*d*_6_ and purified by repeated washing with ice water.

### 2.2 Computational Methods

Geometry optimization of stationary points on the potential energy surface (PES) of the OH reaction with CH_3_N=CH_2_ was made in MP2^25^ and M06-2X density functional^26^ calculations employing Dunning’s correlation-consistent aug-cc-pVTZ basis sets.^27,28^ The subsequent atmospheric reactions were characterized in M06-2X calculations. Energies of stationary points on the reaction surfaces were improved by explicitly correlated CCSD(T) calculations with scaled triples contributions, CCSD(T*)-F12a,^29^ in the following abbreviated CC. Excited states and surface crossings were explored in TD-DFT, CIS, and CASSCF calculations. Additional dipole moments and isotropic polarizabilities, serving as input to prediction of ion–molecule reaction rate coefficients,^30^ were obtained in B3LYP calculations; the results are summarized in Table S1 in the Supporting Information. Reaction enthalpies were calculated using the G4 multilevel method.^31^ The M06-2X (tight optimization criteria and ultrafine integration grids), B3LYP, CIS, CASSCF, MP2, and G4 calculations were performed with Gaussian09^32^ and Gaussian16,^33^ whereas the coupled cluster calculations were carried out with Molpro 2019.2.^34^

Master equation calculations were carried out using the Master Equation Solver for Multi-Energy-well Reactions (MESMER v.4.1)^35^ to simulate the kinetics of the OH radical reactions with CH_3_NCH_2_ and the branching in consecutive reactions under atmospheric conditions. The required input parameters for molecules, intermediate species and products were obtained from the ab initio calculations. Tunneling corrections were approximated in the models employing a one-dimensional asymmetrical Eckart barrier using the method described by Miller.^36^ Rate coefficients for barrierless association reactions were approximated by *k*_association_ = 4.0 × 10^–10^ × (*T*/298 K)^1/6^ from long-range transition state theory.^37^ Spin–orbit coupling in the OH radical (139.7 cm^–1^)^38^ was included in the model by lowering the energy of the OH radical with half of the splitting and including the ^2^P_3/2_ and ^2^*P*_1/2_ spin–orbit states in the electronic partition function. It was assumed that spin–orbit coupling could be neglected in the prereaction adduct and in the saddle points.

Lennard-Jones parameters for the CH_3_N=CH_2_ + OH reactions were approximated by values for methyl acetate (ε = 469.8 K, σ = 4.94 Å)^39^ having a similar number of atoms and dipole moment as the prereaction adduct, and the energy transfer in collisions with N_2_ and O_2_, ⟨Δ*E*_down_⟩, was set to 250 cm^–1^. Variation of these parameters resulted in only insignificant changes in the calculated rate coefficients; changing ⟨Δ*E*_down_⟩ by ±50 cm^–1^ resulted in changes of ±0.5% in the overall rate coefficients; changing the Lennard-Jones parameters by ±50% resulted in changes of <1.5% in the overall rate coefficients.

## 3 Results and Discussion

### 3.1 Computational Results

The initial step in the CH_3_N=CH_2_ reaction with OH radicals will either be an addition to the π-system or a hydrogen abstraction; the reaction enthalpies listed stem from G4 calculations and refer to 1013 mbar and 298 K:
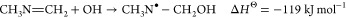
1a
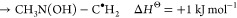
1b
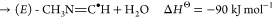
1c
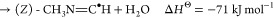
1d
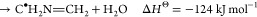
1eFigure 1 illustrates the relative energies of stationary points on the potential energy surface (PES) of the initial CH_3_NCH_2_ + OH reaction; the underlying quantum chemistry data are summarized in Table S2 (energies, *T*_1_(40) and *D*_1_(41,42) diagnostics values, vibrational frequencies, rotational constants, and Cartesian coordinates of the stationary points). The *T*_1_ diagnostic values for the saddle points are all significantly below 0.044 (the largest value being 0.036 for the SP-1c), indicating that the coupled cluster calculations are not seriously affected by multireference problems.^40,42^

**Figure 1:**
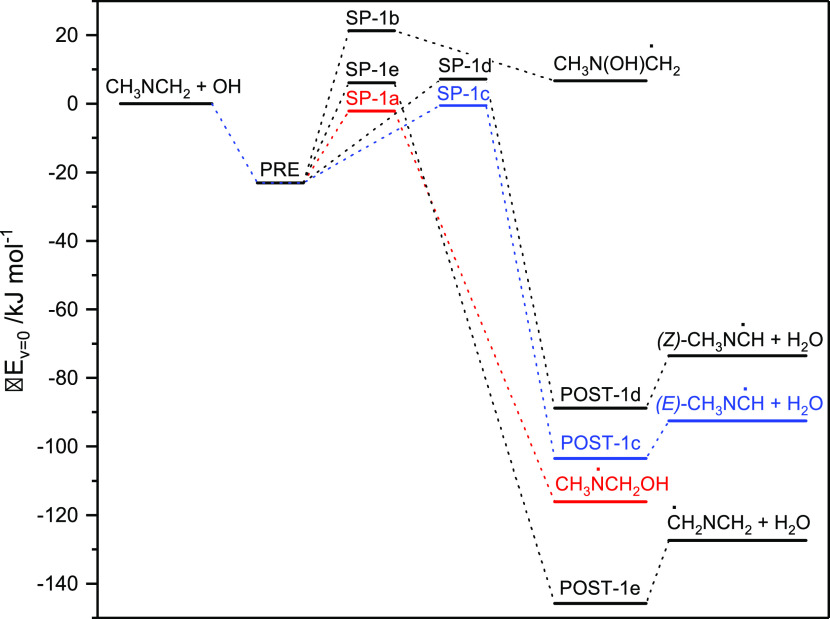
Relative energies of stationary points on the potential energy surface of the CH_3_N=CH_2_ + OH reaction. Results from CCSD(T*)-F12a/aug-cc-pVTZ//M06-2X/aug-cc-pVTZ calculations.

All routes, with the exception of (1b), are calculated to be exothermic proceeding via a common prereaction adduct (PRE), and to have barriers below 10 kJ mol^–1^. The CC//M06-2X results point to reactions 1a and 1c as the more important pathways having submerged saddle points at −2.1 and −0.5 kJ mol^–1^, respectively, whereas reactions 1d and 1e with barriers of 7.0 and 6.1 kJ mol^–1^ will constitute minor pathways. The N-addition route, having a calculated barrier of 21.3 kJ mol^–1^, is of no importance under atmospheric conditions.

The CC//MP2 calculations (Table S2) give somewhat higher barriers of 3.3, 32.2, 0.9, 12.5, and 18.4 kJ mol^–1^, respectively. The MP2 saddle point structures are distinctively closer to the product sides of reaction than the M06-2X structures, and they also show significantly steeper potentials, Table S2. The difference between the CC//MP2 and CC//M06-2X results can conveniently be divided into contributions from the coupled cluster electronic energy (Δ_CC_) and the zero-point energy (Δ_ZPE_) that is negligible for the reactants (Δ_CC_ = −0.1, Δ_ZPE_ = 0.1 kJ mol^–1^), but substantial for PRE and the saddle points SP-1a through SP-1e: Δ_CC_/Δ_ZPE_ = −3.0/17.4, 1.9/3.4, 7.5/3.4, −1.9/3.3, 1.8/3.8, and 5.2/7.2 kJ mol^–1^, respectively. The unusual differences in calculated ZPEs are related to an inappropriate MP2 description of the π-system during the reaction that, in its most extreme, is manifested by bizarre vibrational wavenumbers such as υ̃_C=N_ = 4071 cm^–1^ in PRE and 2724 cm^–1^ in SP-1e, Table S2. The fact that the Δ_CC_ values are relatively small, in spite of some structure differences being >0.1 Å, support the M06-2X description of the MMI + OH PES having wider potentials, over that of MP2.

#### 3.1.1 Kinetics and Branching in the CH_3_NCH_2_ + OH Reaction

The kinetics of the CH_3_NCH_2_ + OH reaction may in principle be governed by both formation of the prereaction adduct and by one or more tight inner transition states. Microcanonical rate coefficients for the inner transition states were calculated using RRKM theory based on energies and rovibrational data from CC//M06-2X calculations. Rate coefficients for the outer transition state were calculated using the inverse Laplace transform of capture rate expressions of the form *k*(*T*) = *C* × (*T*/298 K)^−1/6^ from long-range transition state theory (LRTST)^37^ assuming a dipole–dipole potential (*C* = 4.0 × 10^–10^ cm^3^ molecule^–1^ s^–1^, and calculated dipole moments are collected in Table S1). Long-range transition state theory results represent upper limits to the actual capture rates. Akbar and Barker^21^ studied the influence of the prereaction complex on the reaction of methanimine and OH radicals with canonical variational transition state theory (CVTST) and reported that LRTST overestimated the formation rate by a factor of 2 in this system. The sensitivity of the calculated rate coefficient to variations in the capture rate was tested by varying C between 10^–9^ and 10^–10^ cm^3^ molecule^–1^ s^–1^; only minor changes in the overall and individual rates were found. It can be concluded that the reaction rate is controlled by the inner, tight transition states and that simple capture rate expressions like LRTST or even assuming the gas kinetic collision rate is sufficient for kinetic modeling of the present reaction.

The addition reactions, (1a) and (1b), were treated as reversible isomerization reactions, while the hydrogen abstraction routes (1c)–(1e) were treated as irreversible reactions. The transition states SP-1a, SP-1d, and SP-1e give rise to doubly degenerate reaction paths. The structure of SP-1c also seem to give a degenerate reaction path, but the two saddle points are connected by a rotation of the OH fragment with a small barrier only 0.7 kJ mol^–1^ above the entrance energy of the reactants, and are therefore treated as a single reaction path.

Rotation of the methyl group in MMI is hindered by a barrier calculated to be around 8.9 kJ mol^–1^ (∼740 cm^–1^), which is ∼50 cm^–1^ higher than the experimental value for the CH_3_ rotational barrier in propene.^43−45^ The barrier is slightly higher in the prereaction complex (9.5 kJ mol^–1^) and lowered in the saddle points SP-1a, SP-1c, and SP-1d to 5.8, 7.3, and 7.6 kJ mol^–1^, respectively. On the exit side the CH_3_ rotational barriers are further lowered to 4.0, 2.8, and 5.0 kJ mol^–1^. The barriers to rotation of the OH fragment at the saddle points of reaction are very different in both shape and height; M06-2X calculations reveal barriers ranging from 3 to 25 kJ mol^–1^ (Figure S4).

The calculations imply that the hydrogen abstraction route (1c) leading to (*E*)-CH_3_N=C^•^H is dominant at all temperatures and pressures relevant to the atmosphere. In the harmonic oscillator approximation, the branching between reactions 1a–1e is calculated to be 41:0:53:2:4 with a total rate coefficient of 1.4 × 10^–12^ cm^3^ molecule^–1^ s^–1^ at SATP (298 K, 1000 mbar). Including tunneling in the model increases *k*_SATP_ to 1.9 × 10^–12^ cm^3^ molecule^–1^ s^–1^ and modifies the branching to 39:0:50:3:8. Treating the CH_3_ and OH torsional motions as hindered internal rotors in the master equation calculations and employing the above-mentioned calculated rotational potentials changes *k*_SATP_ to 3.3 × 10^–12^ cm^3^ molecule^–1^ s^–1^ and the branching to 27:0:64:3:6.

Ab initio calculated vibrational frequencies are often multiplied by a scale factor to compensate in part for the electronic structure calculation being approximate and for the potential energy surface not being harmonic. For M06-2X/aug-cc-pVTZ calculations, the recommended scaling factor is 0.958,^46^ and employing this scaling to the vibrational frequencies in the model increases *k*_SATP_ to 3.5 × 10^–12^ cm^3^ molecule^–1^ s^–1^ and alters the branching to 25:0:64:4:7.

The rate coefficient at SATP is comparable to that of the CH_2_=NH + OH reaction, calculated in a similar way (3.0^20^ and 4.0^21^ × 10^–12^ cm^3^ molecule^–1^ s^–1^), and is almost an order of magnitude smaller than the recommended high-pressure value for the CH_3_CH=CH_2_ reaction with OH.^47^ In this context it should be noted that the CH_3_CH=CH_2_ + OH reaction is entirely an addition reaction under atmospheric conditions, whereas the CH_3_N=CH_2_ + OH reaction—like the CH_2_=NH + OH reaction^20,21^—proceeds via both addition and H–abstraction.

Considering an uncertainty of ±4 kJ mol^–1^ in the calculated saddle point heights, we arrive at the following unpretentious limits for the branching ratios, Γ_i_, at 298 K: 7% < Γ_1a_ < 56%, Γ_1b_ < 0.01%, 34% < Γ_1c_ < 90%, Γ_1d_ < 13%, Γ_1e_ < 12%, and an uncertainty factor of 5 for the total rate coefficient (model sensitivity matrix presented in Table S3).

#### 3.1.2 Atmospheric Photo-oxidation

On a global scale, reaction with OH radicals is the dominant gas phase loss process for a majority of tropospheric trace gases.^48^ Other relevant atmospheric oxidants include ozone, Cl atoms, and NO_3_ radicals; the rate coefficient for the Cl atom reaction with MMI has been reported,^17^ and the rate coefficient for NO_3_ radical reaction with MMI can to a first approximation be estimated from the “linear free energy relationship” between OH and NO_3_ radical reactions.^49^

The present theoretical study addresses the OH-initiated photo-oxidation of MMI, the MMI + O_3_ reaction, and the tropospheric photolysis of MMI. Only primary products are considered, and for the sake of simplicity, we have not attended minor routes in the atmospheric photo-oxidation (RO_2_ + RO_2_ → R_–H_O + ROH + O_2_, RO_2_ + RO_2_ → RO + RO + O_2_, RO_2_ + HO_2_ → ROOH + O_2_, RO_2_ + NO_3_ → RO + NO_2_ + O_2_, and RO_2_ + NO → RONO_2_).

##### 3.1.2.1 Fate of the CH_3_N^•^CH_2_OH Radical

The kinetic calculations indicate that ∼30% of the initial CH_3_N=CH_2_ + OH reaction will follow the C-addition route:
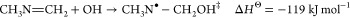
1a

The reaction is highly exothermic, and the activated CH_3_N^•^CH_2_OH^‡^ radical may conceivably isomerize with a rate potentially orders of magnitude faster than any competing bimolecular reactions:
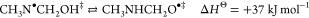
2

The unimolecular isomerization reaction 2 is, however, calculated with a high barrier of around 120 kJ mol^–1^, which roughly places it at the energy of the initial reactants in reaction 1a. Table S4 summarizes the relative energies of stationary points on the CH_3_N^•^CH_2_OH radical formation and subsequent isomerization reaction including the relevant underlying quantum chemistry data. The rate coefficient for isomerization of thermalized CH_3_N^•^CH_2_OH radicals is calculated to be *k*_2_ ≈ 1.6 × 10^–6^ s^–1^ under atmospheric conditions, and a master equation model of reaction 2 shows that less than 0.1% of the activated CH_3_N^•^CH_2_OH^‡^ radicals will actually undergo isomerization before being thermalized. It can therefore be concluded that the isomerization reaction 2 will not be significant under atmospheric conditions.

**Figure 2:**
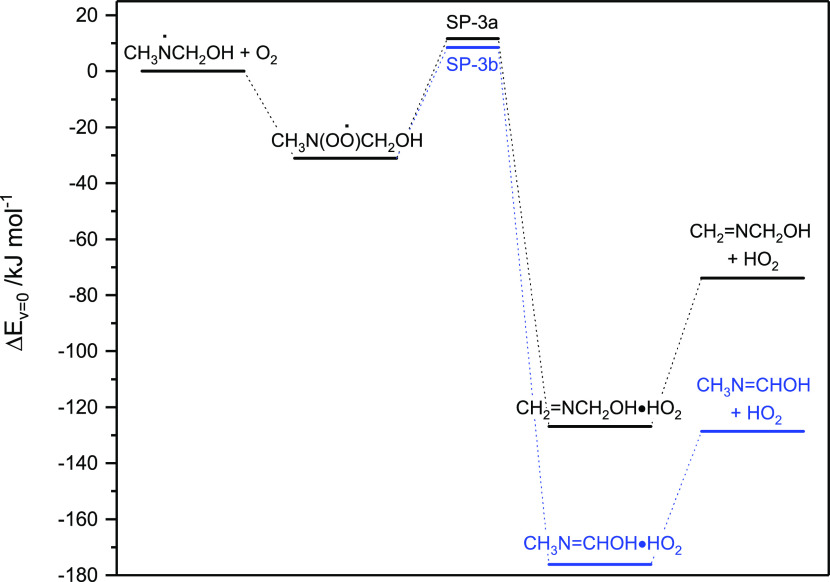
Relative energies of stationary points on the potential energy surface of the CH_3_N^•^CH_2_OH + O_2_ reaction and the subsequent isomerization/dissociation reactions. Results from CCSD(T*)-F12a/aug-cc-pVTZ//M06-2X/aug-cc-pVTZ calculations.

Following results from experimental studies of the CH_3_N^•^CH_3_ radical reactions,^1,2,4,50^ the CH_3_N^•^CH_2_OH radical may conceivably react with O_2_, NO, and NO_2_. There are two routes in the O_2_ reaction, both proceeding via the >NOO^•^ radical on the entrance side, medium sized barriers of respectively 11.7 and 8.5 kJ mol^–1^, and HO_2_ post reaction complexes on the exit side as illustrated in Figure 2 (the underlying quantum chemistry data are summarized in Table S5). For comparison, the barrier to the corresponding CH_3_N^•^CH_3_ + O_2_ reaction is calculated to be 21.5 kJ mol^–1^ at the same level of theory.
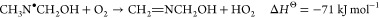
3a
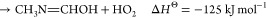
3b

Reaction 3 was investigated in a master equation model based on the PES illustrated in Figure 2. The CH_3_N^•^CH_2_OH + O_2_ association reaction was treated as reversible with *k*_association_ = 10^–10^ cm^3^ molecule^–1^ s^–1^ and the post reaction complexes, CH_2_=NCH_2_OH•HO_2_ and CH_3_N=CHOH•HO_2_, were assumed to dissociate instantaneously to the reaction products; treating dissociation of the postreaction complexes explicitly makes no difference to the outcome of the kinetic modeling. The CNOO torsional mode in CH_3_N(OO^•^CH_2_OH was described as a hindered internal rotor (the potential obtained in M06-2X calculations is shown in Figure S5).

The model predicts *k*_3_ = 1.4 × 10^–14^ cm^3^ molecule^–1^ s^–1^ at 298 K and a branching (3a):(3b) ≈ 1:99 when tunneling is included. The model is not very sensitive to the association rate; reducing *k*_association_ by an order of magnitude lowers the calculated rate coefficient by less than 5%. The model predicts *k*_3_ = 1.2 × 10^–16^ cm^3^ molecule^–1^ s^–1^ at 298 K and a branching (3a):(3b) ≈ 10:90 when tunneling is not integrated; the changed branching ratio is due to quite different imaginary vibrational wavenumbers at the saddle points, Table S5. Uncertainties in the barrier heights were considered by reducing SP-3a by 4 and increasing SP-3b by 4 kJ mol^–1^ at the same time; this extreme results in a branching of 73:27 when tunneling is not integrated in the model.

The CH_3_N^•^CH_2_OH radical reactions with NO and NO_2_ both proceed without electronic barriers:
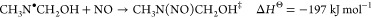
4
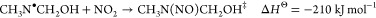
5a
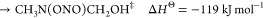
5b

The activated CH_3_N(ONO)CH_2_OH^‡^ is metastable and will dissociate directly without any electronic barrier in addition to the reaction endothermicity:
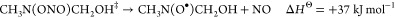
6

Although the CH_3_N^•^CH_2_OH + NO/NO_2_ reactions may be very fast, the loss rate of CH_3_N^•^CH_2_OH radicals due to reaction with O_2_ will be in the range 6 × 10^2^ to 7 × 10^4^ s^–1^ under atmospheric conditions, which in any case will be orders of magnitude faster than the competing reactions with realistic atmospheric ppb-levels of NO and NO_2_. It can be concluded from the theoretical results that that the CH_3_N^•^CH_2_OH radical reaction with O_2_ is so fast that the competing (and barrierless) reactions with NO and NO_2_ are of little importance under atmospheric conditions. That is, insignificant nitrosamine and/or nitramine formation will result in the atmospheric reactions of the CH_3_N^•^CH_2_OH radical. Concerning the branching in reaction 3, the present theoretical calculations cannot predict this accurately.

The two products formed in reaction 3 may in principle both undergo tautomerization reactions. *N*-methylformimidic acid (CH_3_N=CHOH) can tautomerize to the *E*-conformation of *N*-methylformamide via a barrier of around 135 kJ mol^–1^ whereas the 1,3-H transfer in *N*-methanol methaneimine (CH_2_=NCH_2_OH), proceeding via a barrier near 185 kJ mol^–1^, is calculated to dissociate spontaneously to methanimine and formaldehyde:
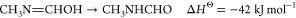
7
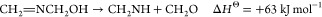
8

Reaction 8 is clearly not relevant under atmospheric conditions, and a master equation model simulation of reaction 7 indicates *k*_7_ × 5 × 10^–7^ s^–1^ for thermalized CH_3_N=CHOH at 1 atm and 298 K (thermal lifetime ∼20 d). The CH_3_N=CHOH tautomerization to CH_3_NHCHO (*N*-methyl formamide) is calculated with a barrier that is slightly higher than found for the corresponding HN=CHOH → H_2_NCHO isomerization^20^ (138.1 vs 119.7 kJ mol^–1^, which results from M06-2X/aug-cc-pVTZ calculations), and will not be significant under atmospheric conditions–even should all the available enthalpy of reaction 3b be deposited in CH_3_N=CHOH.

In summary, the theoretical calculations locate CH_2_=NCH_2_OH and CH_2_N=CHOH as the dominating products resulting from the OH addition reaction 1a with <10% of the former and >90% of the latter. However, extreme conservative limits to the yields are <75% and >25%.

##### 3.1.2.2 Fate of the CH_3_NC^•^H radical

Around 70% of reaction 1 is predicted to give CH_3_N=C^•^H radicals that are formed predominantly as the low energy *E*-isomer; see Figure 1. There is a barrier of around 35 kJ mol^–1^ between the *Z*-isomer having around 19 kJ mol^–1^ higher energy than the *E*–isomer, and the unimolecular *Z* → *E* conversion rate at thermal equilibrium is estimated to be around 4 × 10^5^ s^–1^. Since the subsequent reactions of the *Z*- and *E*-isomers are the same, we only consider the low energy *E*-isomer in the following.

Direct H-ejection from the CH_3_N=C^•^H radical is highly endothermic and can therefore be neglected under atmospheric conditions:
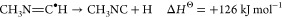
9

The main atmospheric sink for CH_3_N=C^•^H is therefore reaction with O_2_. Two routes have been identified: direct H-abstraction, resulting in CH_3_NC, and the formation of an activated peroxy radical:
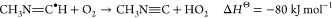
10a
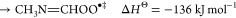
10b

The H-abstraction reaction proceeds via a submerged barrier (SP-10a, Δ*E*_elec_ = −3 kJ mol^–1^) linked to a weak prereaction adduct on the entrance side (PRE-10a, Δ*E*_elec_ = −6 kJ mol^–1^, basis set superposition error ≈0.8 kJ mol^–1^) and to a H-bonded HO_2_ radical complex on the exit side. The vibrational zero-point energy of the prereaction adduct PRE-10a is around 5 kJ mol^–1^ larger than that of the saddle point SP-10a, apparently placing Δ*E*_*v*=0_(PRE-10a) > Δ*E*_*v*=0_(SP-10a). However, the *T*_1_ diagnostic value for PRE-10a is 0.059, suggesting that the results of the coupled cluster calculations should be considered with caution.

There are two conformations of the CH_3_N=CHOO^•^ radical separated by a few kJ mol^–1^ barrier–the low energy form has a synperiplanar HCOO moiety (*syn*); the high energy form (∼+16 kJ mol^–1^) has an antiperiplanar HCOO moiety (*anti*). The activated peroxy radical may initiate internal H-shift reactions with barriers below the entrance energy in reaction 10:
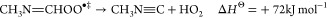
11a
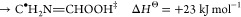
11b

The M06-2X calculations find the C^•^H_2_N=CHOOH radical to be metastable with an electronic barrier of only 7.5 kJ mol^–1^ to dissociation:
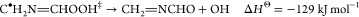
12

The couple cluster calculations, however, reverse the energies to −2.3 kJ mol^–1^. Since the *T*_1_-values are below 0.025 for both structures, we suggest that the alleged electronic barrier is an artifact of the M06-2X functional.

**Figure 3:**
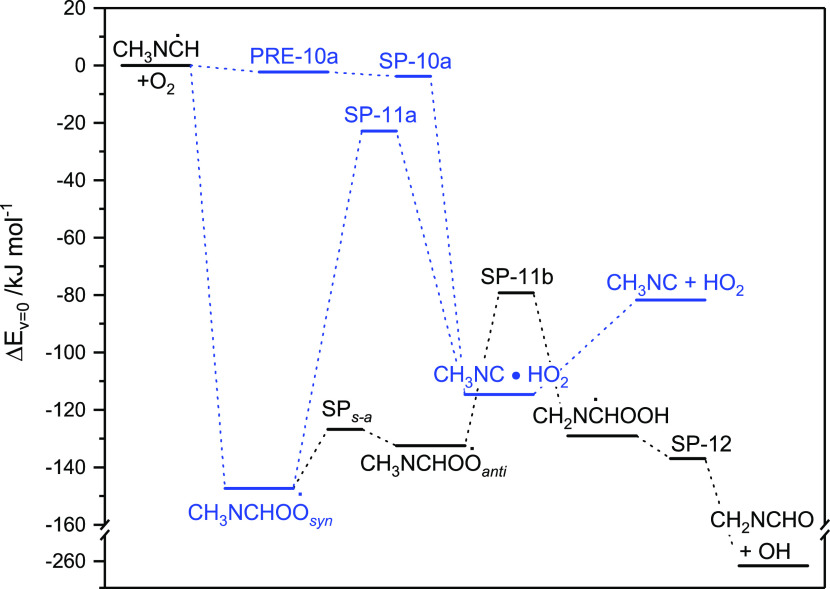
Relative energies of stationary points on the potential energy surface of the *E*-CH_3_NC^•^H + O_2_ reaction. Results from CCSD(T*)-F12a/aug-cc-pVTZ//M06-2X/aug-cc-pVTZ calculations.

The CH_3_N=CHOO^•^ peroxy radical may also react with NO to form the corresponding oxy radical that may either eject an H atom directly resulting in methyl isocyanate or undergo H-abstraction by O_2_ to give the same product. H-ejection is endothermic and proceeds essentially without any additional electronic barrier.
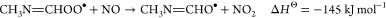
13
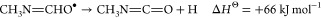
14
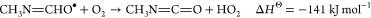
15Figure 3 shows the relative energies of stationary points on the CH_3_N=C^•^H + O_2_ PES; the underlying data are documented in Table S6. The CH_3_N=C^•^H + O_2_ reaction sequence, (10)–(12), was modeled in master equation calculations based on the PES illustrated in Figure 3, and including the sequence (13)–(15) as a competing RO_2_-sink. The calculations reveal that direct H-abstraction (10a) is 2 orders of magnitude slower than the RO_2_-routes initiated via (10b)—even when lowering the energy of PRE-10a by 20 kJ mol^–1^—and that route (11b) dominates the atmospheric fate of the CH_3_N=CHOO^•^ radical with a yield of >98%.

In conclusion, under atmospheric conditions *N*-methyleneformamide, CH_2_=NCHO, will be the by far dominant product following H-abstraction from the CH_2_-group in MMI.

##### 3.1.2.3 Fate of the CH_2_NC^•^H_2_ radical

Less than 5% of the initial CH_3_N=CH_2_ + OH reaction is predicted to result in CH_2_NC^•^H_2_ radicals that, under atmospheric conditions, will react with O_2_ forming an activated peroxy radical:
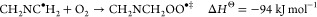
16The addition reaction appears without any electronic barrier, and the activated peroxy-radical may undergo unimolecular reactions before being thermalized by collisions or entering bimolecular reactions. Potentially, a 1,5-H transfer may be followed by either H-ejection or dissociation:
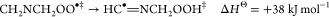
17
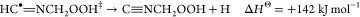
18a
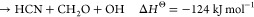
18b

**Figure 4:**
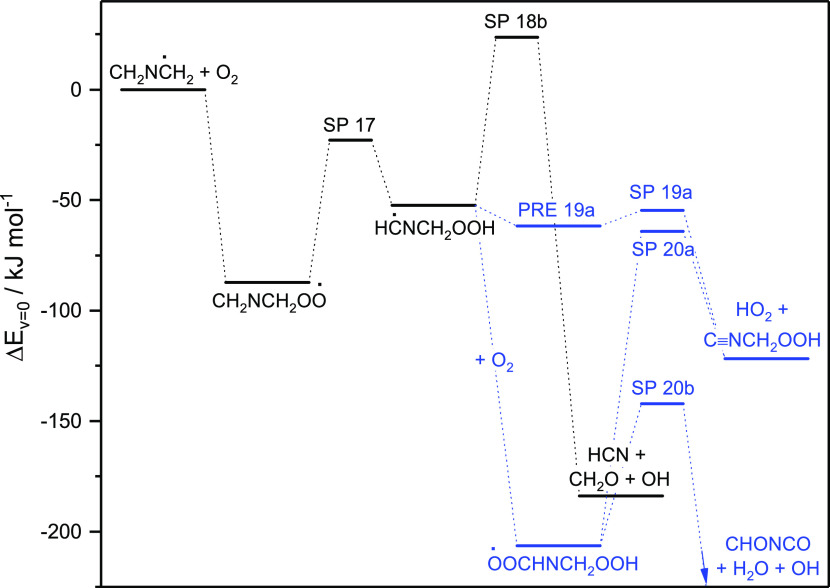
Relative energies of stationary points on the potential energy surface of the CH_2_=NC^•^H_2_ + O_2_ reaction. Stationary points in black include the energy of an additional O_2_. Results from CCSD(T*)-F12a/aug-cc-pVTZ//M06-2X/aug-cc-pVTZ calculations.

The endothermic 1,5–H transfer reaction 17 has a barrier well below the entrance energy of the initial reactants, but the subsequent unimolecular reactions of HC^•^=NCH_2_OOH are hindered by barriers above the entrance energy. There is also a relatively high barrier of around 140 kJ mol^–1^ to direct H-ejection, and this route will therefore not be relevant under atmospheric conditions. Finally, the dissociation reaction 18b is not a simple unimolecular dissociation; the quantum chemistry calculations show an initial barrier of around 20 kJ mol^–1^ above the entrance energy to give HCN and the metastable C^•^H_2_OOH radical, which then dissociates to CH_2_O and OH. The latter fine details have not been included in Figure 4 illustrating the relative energies of the stationary points on the PES of the CH_2_NC^•^H_2_ + O_2_ reaction (energies and Cartesian coordinates of the stationary points of the reaction are summarized in Table S7).

As a consequence of the significant barriers to reaction 18, the atmospheric fate of CH_2_NCH_2_OO^•^ radicals will be determined by the competition between collisional quenching, reaction with NO, and the O_2_ reaction with HC^•^=NCH_2_OOH radicals. The latter autoxidation may either proceed via a direct or an indirect H-abstraction leading to C≡NCH_2_OOH, or via an activated O^•^OCH=NCH_2_OOH^‡^ peroxy-radical and a second internal 1,5–H transfer resulting in HOOCH=NC^•^HOOH^‡^, which is found to spontaneously undergo an extremely exothermic internal reaction resulting in CHONCO (formyl isocyanate) and H_2_O and in regeneration of the OH radical:
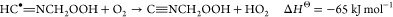
19a
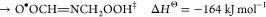
19b
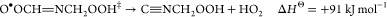
20a
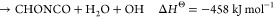
20b

The relative energies of the stationary points on the PESes of reactions 19 and 20 are included in Figure 4 (energies and Cartesian coordinates of the stationary points of the reactions are found in Table S7). Reaction 19a proceeds via a weak prereaction complex, a submerged barrier, and a postreaction HO_2_ complex; for the sake of legibility, the postreaction complex has been omitted from Figure 4.

The CH_2_=NC^•^H_2_ + O_2_ reaction sequence (16)–(20) was modeled in master equation calculations based on the PES illustrated in Figure 4 and including the peroxy radical removal by NO:
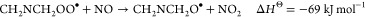
21

Typical rate coefficients for the R + O_2_ → RO_2_ and RO_2_ + NO → RO + NO_2_ reactions (5 × 10^–12^ to 10^–11^ cm^3^ molecule^–1^ s^–1^ at 298 K^51^) and an NO level of 10 ppbV were employed in modeling the competing reactions. RRKM calculations give a thermal rate coefficient *k*_19a_ ≈ 3 × 10^–14^ cm^3^ molecule^–1^ s^–1^ at 298 K, which is orders of magnitude too slow to compete with reaction 19b. It is also obvious that reaction 20b will be orders of magnitude faster than reaction 20a and that CHONCO therefore will be the by far dominant product (>99.9%) in the HC^•^=NCH_2_OOH + O_2_ reaction.

Concerning the branching between routes 17–19 and 21, the master equation calculations forecast a maximum CH_2_NCH_2_O^•^ yield of 15% under atmospheric conditions assuming an NO level of 10 ppb; under chamber conditions with ∼50 ppbV NO, the yield could be up to 50%.

The oxy-radical formed in (21) may either dissociate or undergo H-abstraction by O_2_:
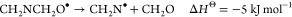
22
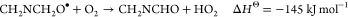
23

The barrier to the N–C scission, reaction 22, is calculated to be well below the entrance energy of reactants in reaction 21, and the fate of the CH_2_NCH_2_O^•^ radical will therefore depend on pressure and the energy partitioning in reaction 21. Figure S6 shows the relative energies of the stationary points on the PES of the CH_2_NCH_2_OO^•^ + NO reaction; energies and Cartesian coordinates are found in Table S8.

Master equation calculations were carried out to estimate the branching ratio (22):(23) at typical atmospheric conditions. For equipartitioning of the reaction enthalpy in reaction 19 the (22):(23) branching ratio is calculated to be 97:3 under atmospheric pressure and ⟨Δ*E*_down_⟩ = 250 cm^–1^. The fundamental modes of vibration in NO_2_ are around 750, 1318, and 1618 cm^–1^. Assuming that the product NO_2_ has one quantum of the antisymmetric stretching mode (∼19 kJ mol^–1^) and that the remaining reaction enthalpy is equipartitioned, the (22):(23) branching is calculated to be around 50:50. There are no experimental data in the literature on how the energy is distributed in ROO + NO reactions, and the theoretical study thereof can therefore only indicate limits to the atmospheric fate of CH_2_NCH_2_O^•^ radicals: >50% HCN + CH_2_O and <50% CH_2_=NCHO.

In summary, more than 85% of the CH_2_NC^•^H_2_ radicals, formed in H-abstraction from the −CH_3_ group in MMI, will result in CHONCO, while less than 15% will result in HCN, CH_2_O, and CH_2_=NCHO.

##### 3.1.2.4 CH_3_N=CH_2_ Reaction with O_3_

The 1,3-dipolar cycloaddition of ozone to a double bond is challenging to describe accurately in quantum chemistry calculations due to the high multireference character of ozone and the transition states.^52^ Nonetheless, Wheeler et al. showed that several multilevel methods perform well for such reactions.^53^ We have previously employed the G4 approach to compare the barriers to the O_3_ reactions with CH_2_=CH_2_ and CH_2_=NH,^20^ and we recognized that the HOMO–LUMO gap is more than 100 kJ mol^–1^ larger in the imine than in the corresponding alkene and that this impacts the thermochemistry of all steps in the reaction: 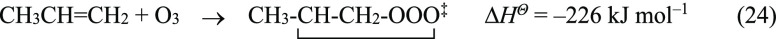
24
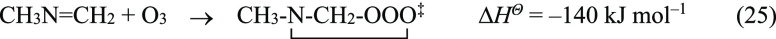
25Table S9 compares energies of the stationary points on the PES for the two systems. Both reactions proceed via weak van der Waals complexes and distinctive barriers to formation of the primary ozonides. The barrier to formation of the primary ozonide is significantly higher for MMI (Δ*E*^†^_Elec+ZPE_ = 38.3, Δ*G*^†^_298_ = 87.6 kJ mol^–1^) than for propene (Δ*E*^†^_Elec+ZPE_ = 15.2, Δ*G*^†^_298_ = 61.9 kJ mol^–1^). Accordingly, the reactivity toward ozone is obviously lower, and Transition State Theory predicts the rate coefficients to be 1.1 × 10^–22^ cm^3^ molecule^–1^ s^–1^ for the CH_3_N=CH_2_ + O_3_ reaction and 3.5 × 10^–18^ cm^3^ molecule^–1^ s^–1^ for the CH_3_CH=CH_2_ + O_3_ reaction, for which the recommended rate coefficient is 1.6 × 10^–18^ cm^3^ molecule^–1^ s^–1^ at 1 atm and 298 K.^54^ This gives confidence in the computational approach, and even allowing for a significant error in the calculated barrier to the CH_3_N=CH_2_ + O_3_ reaction, it can be concluded that the reaction is too slow to be of any importance under atmospheric conditions—MMI is “essentially non-reactive toward O_3_”.^15^

##### 3.1.2.5 Tropospheric Photolysis

TDDFT calculations^55^ employing the B3LYP functional place the lowest vertical singlet excitation energy in MMI (n → π* transition) at 255 nm with an oscillator strength *f* = 0.0005. The corresponding vertical excitation energy in CH_2_=NH is calculated at 245 nm with an oscillator strength *f* = 0.0019, which compares well to the experimental observation of a broad and structureless band with a maximum absorption cross section ∼4 × 10^–19^ cm^2^ molecule^–1^ near 250 nm.^56^ Assuming a Gaussian line profile with 10 nm half-width, the calculated absorption cross sections of both MMI and CH_2_=NH just about extend into the actinic region with absorption cross sections becoming <10^–20^ cm^2^ molecule^–1^ at 290 nm and <10^–21^ cm^2^ molecule^–1^ at 310 nm. The actinic flux in the 290–310 nm region is below 10^14^ photons cm^–2^ nm^–1^ for a solar zenith angle θ = 0°,^48^ and tropospheric photolysis of MMI can therefore, at best, only be efficient in a very few regions of the Earth.

As in CH_2_=NH,^57^ there is conical intersection between the S_0_ and S_1_ potential surfaces of MMI with the minimum energy crossing point (MECP) located close to the S_1_ potential energy minimum. This indicates that an excitation to the S_1_ state will be followed by vibrational relaxation and a very rapid radiationless crossing to S_0_, where at most 400 kJ mol^–1^ (λ = 300 nm) then will be available to dissociation processes before collisional quenching establishes thermal equilibrium:
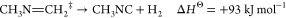
26a
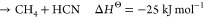
26b
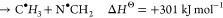
26c
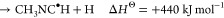
26d

There are two routes to H_2_ elimination (*E*- and *Z*-saddle point configurations) having barriers of 364 and 342 kJ mol^–1^, respectively; there is no electronic barrier to CN-scission in addition to the endothermicity, and the CH_4_ + HCN route is located with a barrier of 340 kJ mol^–1^ (CC//M06-2X results, Table S10). The conceivable tropospheric photolysis processes will therefore be completely dominated by route 26c, where the N^•^CH_2_ radical subsequently will undergo H-abstraction by O_2_ resulting in HCN:
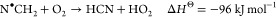
27

Schade and Crutzen considered route 26a in their reflections on routes to N_2_O formation in the atmospheric degradation of methylamines.^11^ The present results clearly demonstrate that high barriers block this route. In addition, a recent experimental and theoretical study of the atmospheric chemistry CH_3_NC shows CH_3_NCO as the only product.^22^

##### 3.1.2.6 Photo-oxidation Mechanism

**Scheme 1:**
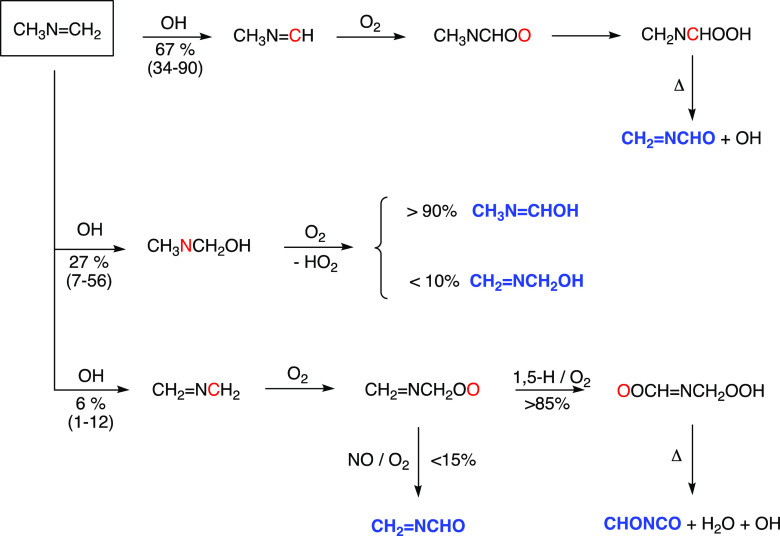
Major Routes for the OH-Initiated Photo-oxidation of CH_3_N=CH_2_ under Atmospheric Conditions as Resulting from Theoretical Calculations Conservative limits to estimated branchings are given in parentheses; thermally stable products are typeset in bold blue font; radical sites are indicated in red font.

The theoretically predicted major atmospheric degradation routes of MMI are outlined in Scheme 1 and include the ab initio calculated branching ratios with estimated range limits. The mechanism, originating in quantum chemistry and master equation calculations, displays little resemblance to that proposed by Schade and Crutzen,^11^ who did not consider abstraction from the =CH_2_ group, which we find to be a dominant route. The major primary products in atmospheric MMI photo-oxidation are predicted to be other imines: CH_2_=NCHO (*N*-methyleneformamide) and CH_3_N=CHOH (*N*-methylformamidic acid). The latter is a tautomer of *N*-methylformamide, but the barrier, being around 135 kJ mol^–1^, slows tautomerization resulting in a thermal lifetime ∼20 d in the gas phase.

The predicted photo-oxidation products allow an experimental determination of the branching in reaction 1: CHONCO (formyl isocyanate) is unique to the CH_3_-abstraction route; CH_3_N=CHOH and CH_2_=NCH_2_OH (methyleneamino-methanol)—having the same sum formula–are unique to the C-addition route; CH_2_=NCHO (*N*-methyleneformamide) is not unique to the CH_2_–abstraction route, but for all practical purposes it is, as the contribution from the CH_3_-abstraction route will be minute.

### 3.2 Experimental Results

#### 3.2.1 EUPHORE Experiments

Six MMI photo-oxidation experiments were carried out in the 200 m^3^ EUPHORE atmospheric simulation chamber. The attempts to determine the MMI photo-oxidation products unambiguously were unconvincing due to (1) the slow monomer–trimer equilibration in the simulation chamber, (2) surface reactions, and (3) prominent particle formation. The experiments were, however, not without intellectual value.

TMT was not identified in any chamber experiments by PTR-ToF-MS (C_6_H_16_N_3_^+^, *m*/*z* 130.134). This is a natural consequence of the TMT ⇄ 3 MMI equilibrium being strongly temperature dependent (Δ*H*_exp_ ∼ 150,^14^ Δ*G*_calc_ = 95, Δ*H*_calc_ = 177; all in kJ mol^–1^), the subppm level TMT concentrations in the experiments and the surface temperatures of the PTR instrument inlet and detection system; an initial 1 ppm V TMT will equilibrate to ∼30% trimer at room temperature; at 75 °C the equilibrium is shifted to <0.1% TMT.

**Figure 5:**
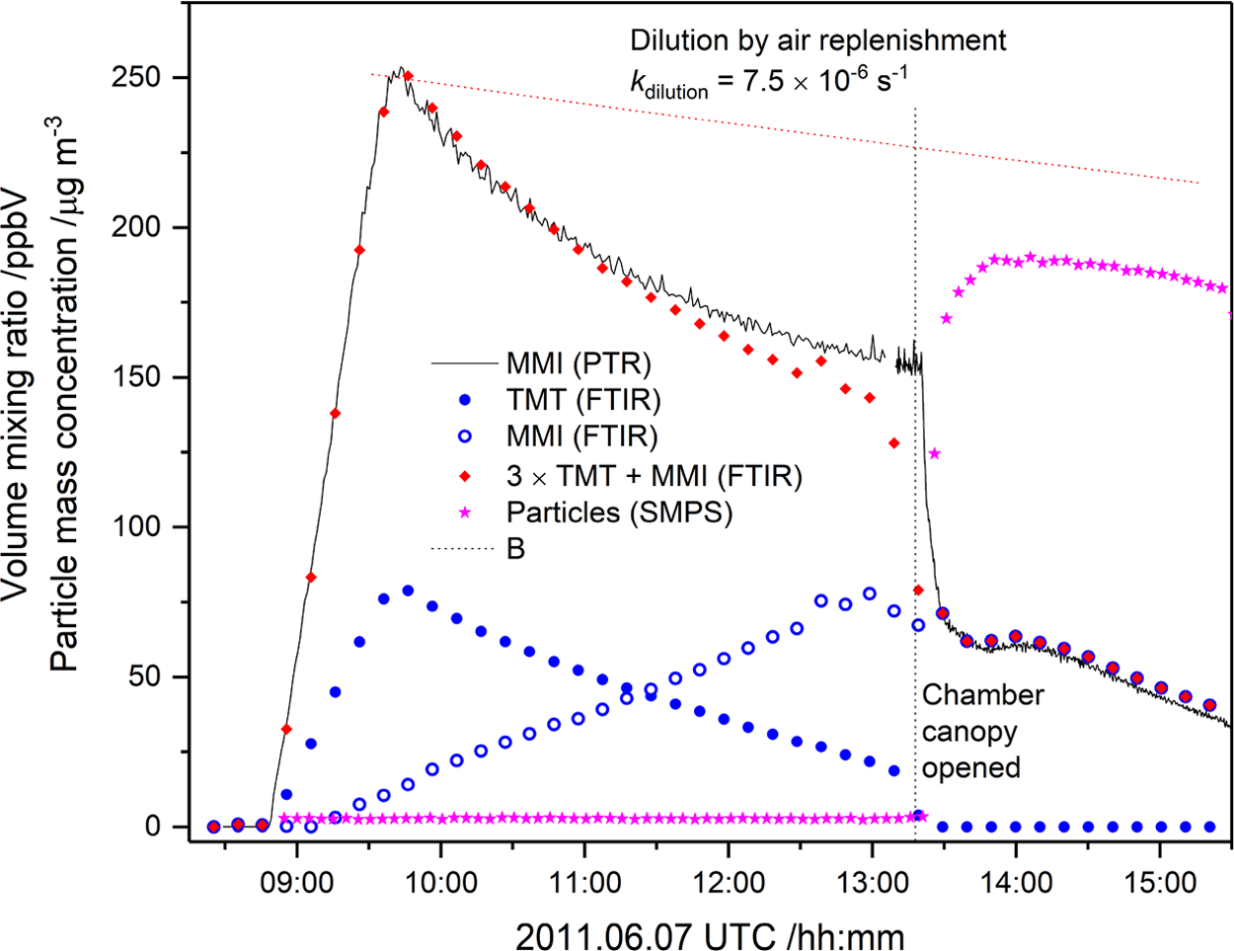
Comparison of 1,3,5-trimethyl-1,3,5-triazinane (TMT) and *N*-methylmethanimine (MMI) volume mixing ratios obtained by PTR-TOF-MS and FTIR, and the temporal particle mass concentration during the 2011.06.07 photo-oxidation experiment in the EUPHORE atmospheric simulation chamber B.

Figure 5 compares the time profiles of MMI and TMT independently obtained by FTIR and PTR-ToF-MS (protonated MMI, C_2_H_6_N^+^, *m*/*z* 44.050) during an EUPHORE experiment. In this particular experiment, 170 mg TMT was injected in an airstream to the chamber and left for nearly 4 h before the OH precursor IPN-d6 was added and the chamber canopy opened to sunlight ((CD_3_)_2_CHONO + *h*ν → (CD_3_)_2_CHO^•^ + NO; (CD_3_)_2_CHO^•^ + O_2_ → (CD_3_)_2_CO + HO_2_; HO_2_ + NO → OH + NO_2_). During this period the SMPS (Scanning Mobility Particle Sizer) showed only a minute gas-to-particle transfer, while the FTIR showed around 75% reduction in TMT and a less than stoichiometric increase in MMI. That is, an appreciable amount of TMT and/or MMI was lost to the chamber walls before the photo-oxidation was initiated by opening the chamber canopy. This is also reflected in the PTR-TOF-MS signal that correlates well with the sum MMI + 3 × TMT from FTIR; the temporal MMI signal shows an exponential decay with a rate of 3.5 × 10^–5^ s^–1^, which is around 5 times larger than the chamber dilution by replenishment air.

**Figure 6:**
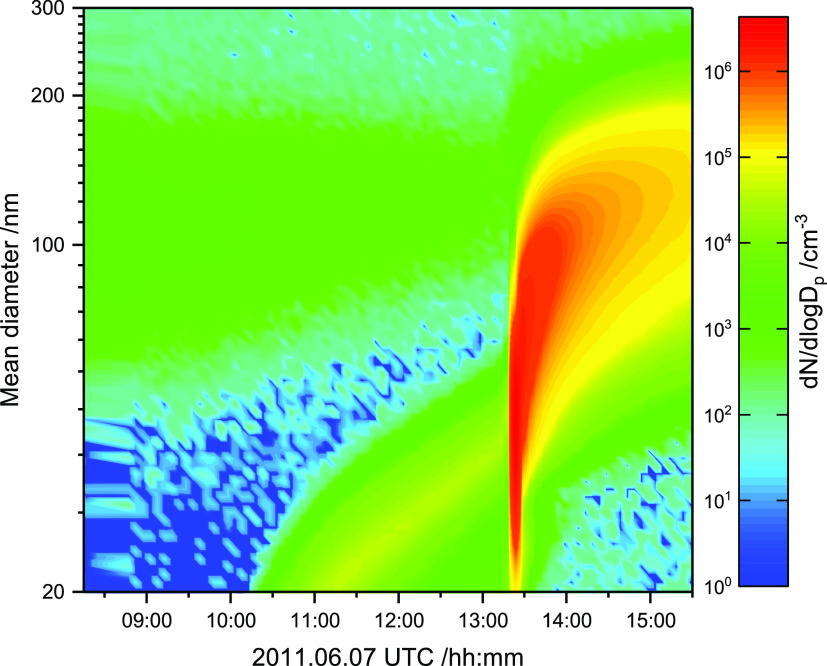
Particle number concentration and particle size distribution from SMPS measurements during the 2011.06.07 photo-oxidation experiment in the EUPHORE atmospheric simulation chamber B.

TMT is a tertiary (cyclic) triamine and is therefore expected to react very fast with OH radicals, *k*_TMT+OH_ > 5 × 10^–11^ cm^3^ molecule^–1^ s^–1^.^9^ When the chamber canopy was opened to solar radiation (∼13:20 UTC, Figure 5), the remaining gas phase TMT reacted within 20 min, whereas the MMI showed a more sedate decay. Figure 5 also includes the SMPS results for the total particle mass concentration during the experiment, while Figure 6 shows the particle number concentration and particle size distribution. It can be seen that the very fast TMT loss is paralleled by a steep increase in particle mass concentration to around 175 μg m^–3^, which hypothetically corresponds to ∼25 ppb TMT being transferred from the gas to the particle phase as 1:1 TMT:HNO_3_ salt. MMI, being a strong base, will also transfer to the particle phase. However, Figure 5 suggests that only a small amount of MMI is transferred to the particles in the initial phase of the photo-oxidation experiment.

**Figure 7:**
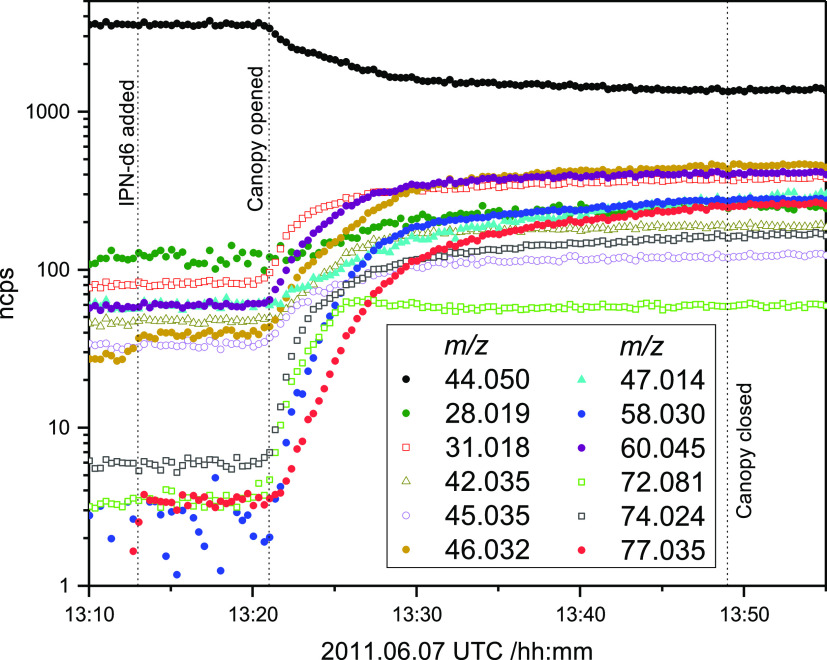
Normalized ion counts registered by PTR-ToF-MS during the 2011.06.07 photo-oxidation experiment in the EUPHORE atmospheric simulation chamber B.

The temporal PTR-ToF-MS ion signals observed in the 2011.06.07 experiment are illustrated in Figure 7, and the PTR-MS results from the six experiments are summarized in Table 1 containing ion signals having an intensity >1% of the decrease in the TMT/MMI signal *m*/*z* 40.050 during the time the chamber canopy was open. It is emphasized that there are no indications of the nitrosamine, CH_3_N(NO)CH_2_OH, or of the nitramine, CH_3_N(NO_2_)CH_2_OH, which potentially could result in the photo-oxidation of MMI; see section 3.1.2.1. It should also be noted that particles to some degree can evaporate in the heated sampling lines and, in particular, in the drift tube of the PTR-MS analyzer.^58^ Some of the ion signals reported in Table 1 and Figure 7 may therefore, at least in part, have their origin in the particle phase.

**Table 1:**
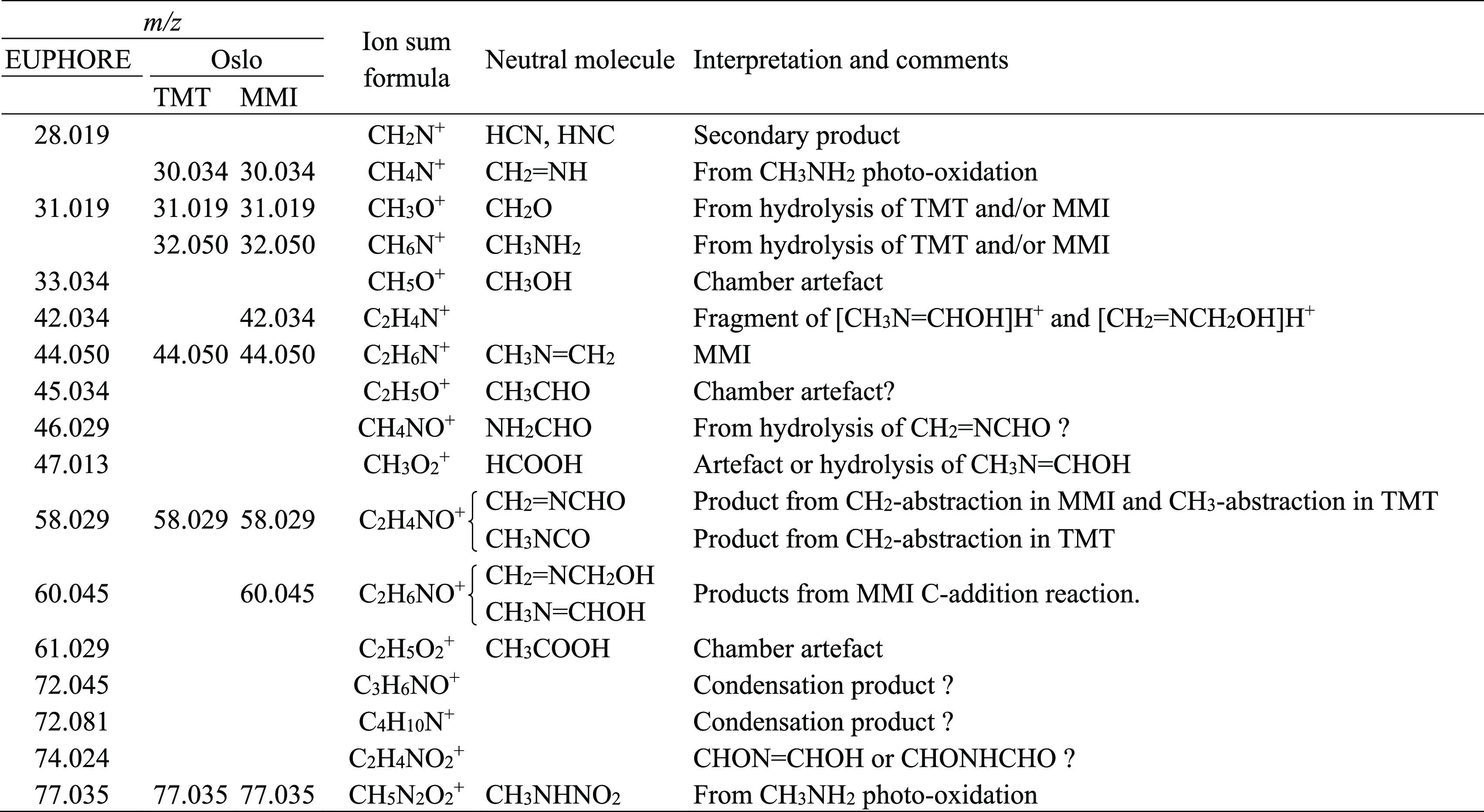
Ion Signals Observed in *N*-Methylmethanimine (MMI) and 1,3,5-Trimethyl-1,3,5-triazinane (TMT) Photo-oxidation Experiments^a^

a Only ion signals having an intensity >1% of the decrease in the MMI signal *m*/*z* 44.050 during the time of reaction are included in the table. Fragment ions, ^13^C-containing ions, instrument-intrinsic ions, and ions arising from side reactions are not included.

The ion signals can be divided into two main groups: (1) *m*/*z* 31.019, 33.034, 42.034, 46.029, and 72.081 that are distinctly correlated with TMT before opening the chamber canopy and anticorrelated after; (2) *m*/*z* 45.034, 58.029, 72.045, and 77.035 that only increase after opening the chamber canopy. The most striking signal is that of *m*/*z* 77.035 (CH_5_N_2_O_2_^+^), which will be addressed later. The *m*/*z* 28.019 (CH_2_N^+^) is burdened by a high background, but has the temporal profile of a secondary product. Finally, the *m*/*z* 72.081 (C_4_H_10_N^+^) has distinct temporal signal profile in all experiments and is clearly the result of heterogeneous processing. In conclusion, most of the ion signals observed in the EUPHORE experiments likely have several origins making mechanistic deductions irrational.

#### 3.2.2 Oslo Smog Chamber Experiments

A series of low concentration experiments were carried out in the Oslo stainless steel reactor to establish a distinction between products from TMT and from MMI photo-oxidation and various artifacts related to possible surface and particle reactions. The disadvantage of metallic surfaces in relation to bases like MMI and TMT is to some extent countered by ease of cleansing the walls, interfacing preparative equipment, and selection of photolysis light sources.

**Figure 8:**
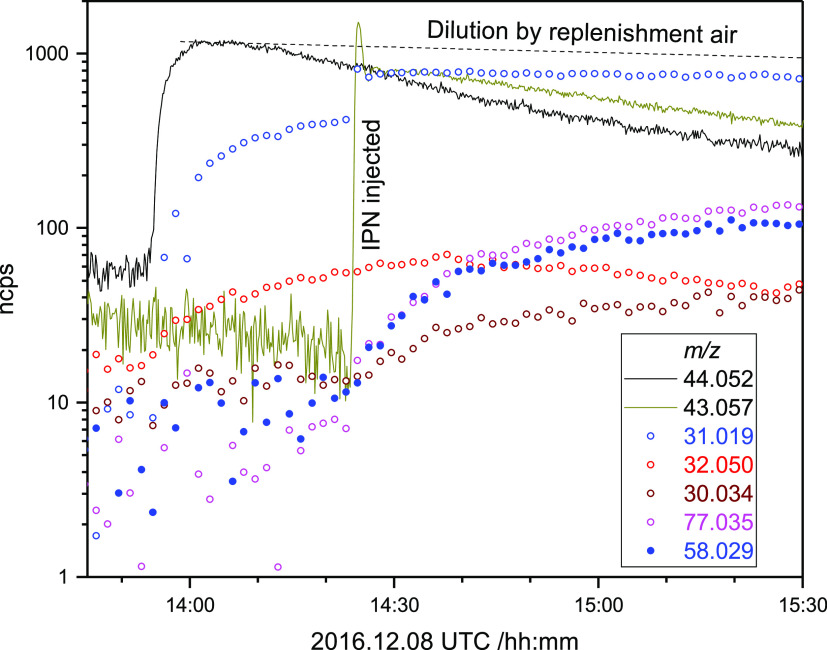
Normalized ion counts registered during the high-NOx 1,3,5-trimethyl-1,3,5-triazinane (TMT) photo-oxidation experiment on 2016.12.08. Signals: *m*/*z* 44.052 (C_2_H_6_N^+^, protonated CH_3_N=CH_2_), 43.057 (C_3_H_7_^+^, fragment of IPN), 31.019 (CH_3_O^+^, protonated CH_2_O), 32.050 (CH_6_N^+^, protonated CH_3_NH_2_), 30.034 (CH_4_N^+^, protonated CH_2_=NH), 77.035 (CH_5_N_2_O_2_^+^, protonated CH_3_NHNO_2_), and 58.029 (C_2_H_4_NO^+^, protonated CH_3_NCO and/or CH_2_=NCHO).

Low concentration TMT photo-oxidation experiments were performed by first injecting TMT into the 350–400 nm irradiated chamber followed by injecting the OH precursor IPN. Figure 8 illustrates the results of an experiment in which TMT was administered to the chamber to around 25 ppbV in clean air, from which it can be seen that there is the foreseeable, extensive loss of TMT to the chamber walls, making quantification of yields futile.

The expected primary photo-oxidation products of TMT (1,3,5-trimethyl-1,3,5-triazinen-2-one, TMTCO, and 3,5-trimethyl-1,3,5-triazinena-1-carbaldehyde,TMTCHO, see Scheme S1) are in equilibrium with their monomeric constituents TMTCO ⇄ 2MMI + CH_3_NCO (Δ*G*_TMTCO,calc_ = 92_,_ Δ*H*_TMTCO,calc_ = 209 kJ mol^–1^) and TMTCHO ⇄ 2MMI + CH_2_=NCHO (Δ*G*_TMTCHO,calc_ = 111_,_ Δ*H*_TMTCHO,calc_ = 228 kJ mol^–1^). Like TMT, neither TMTCO nor TMTCHO were detected directly by the PTR-ToF-MS instrument employed; in fact, no relevant ion signals above *m*/*z* 78 were detected in any experiment.

In addition to ion signals related to IPN and TMT, only five ion signals above 10 normalized counts per second (ncps) were observed with temporal profiles correlated to the injections: (1) *m*/*z* 32.050 (CH_6_N^+^) and 31.018 (CH_3_O^+^) that both started to grow as soon as TMT was injected and (2) *m*/*z* 30.034 (CH_4_N^+^), 77.035 (CH_5_N_2_O_2_^+^), and 58.029 (C_2_H_4_NO^+^) that started to grow when IPN was injected, Figure 8. The group 1 ion signals are recognized as protonated CH_3_NH_2_ and CH_2_O that are formed by hydrolysis of TMT on the chamber surfaces; later, photo-oxidation of IPN also contributes to the *m*/*z* 31.018 ion signal. The group 2 signals *m*/*z* 30.034 and 77.035 are familiar from CH_3_NH_2_ photo-oxidation experiments and relate to protonated CH_2_=NH and CH_3_NHNO_2_.^4^ Finally, the *m*/*z* 58.029 is interpreted as protonated CH_3_NCO and/or CH_2_=NCHO—the two monomeric components of the expected primary TMT photo-oxidation products TMTCO and TMTCHO.

**Figure 9:**
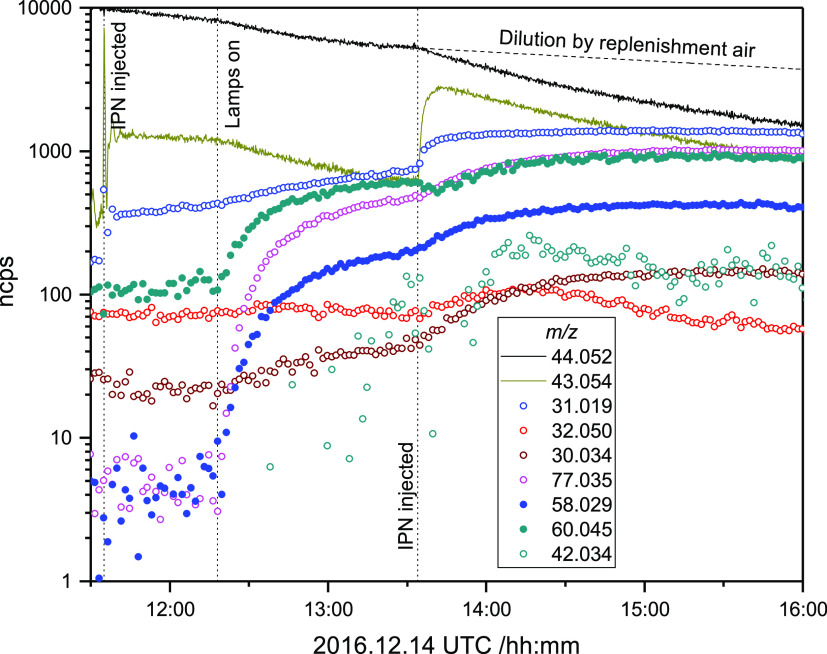
Normalized ion counts registered during the *N*-methylmethanimine (MMI) photo-oxidation experiment on 2016.12.14. Signals: *m*/*z* 44.052 (C_2_H_6_N^+^, protonated CH_3_N=CH_2_), 43.054 (C_3_H_7_^+^, fragment of IPN), 31.019 (CH_3_O^+^, protonated CH_2_O), 32.050 (CH_6_N^+^, protonated CH_3_NH_2_), 30.034 (CH_4_N^+^, protonated CH_2_=NH), 77.035 (CH_5_N_2_O_2_^+^, protonated CH_3_NHNO_2_), 58.029 (C_2_H_4_NO^+^, protonated CH_3_NCO and/or CH_2_=NCHO), 60.045 (C_2_H_6_NO^+^, protonated CH_3_N=CHOH and/or CH_2_=NCH_2_OH), and 42.034 (C_2_H_4_N^+^, fragment of protonated CH_3_N=CHOH and/or CH_2_=NCH_2_OH).

The MMI photo-oxidation experiments were performed by directing heated TMT/MMI vapor via dry ice traps directly into the evacuated chamber, which was then filled with clean air to atmospheric pressure before adding IPN and turning the photolysis lamps on. Figure 9 illustrates the PTR results from an experiment in which MMI was added to the chamber to achieve a mixing ratio of around 500 ppb (quantified by both FTIR and PTR). It is highly important that the FTIR spectra recorded during the experiment illustrated do not show any spectral features attributable to TMT. Again, it is emphasized that there are no indications of the nitrosamine, CH_3_N(NO)CH_2_OH, or of the nitramine, CH_3_N(NO_2_)CH_2_OH, which potentially could result in the photo-oxidation of MMI.

As in the TMT experiments, there is a clear loss of MMI to the chamber walls, making it difficult to assess mass balance in the experiment; the MMI wall loss is apparently roughly at the same level as the dilution by air replenishment. This is also evidenced by the visibly reduced CH_2_O and CH_3_NH_2_ formation from MMI hydrolysis.

Only two ion signals above 10 ncps were detected in addition to the ones observed in the “pure” TMT experiments: *m*/*z* 60.049 (C_2_H_6_NO^+^) and 42.034 (C_2_H_4_N^+^). The former signal, corrected for isotope interference from IPN and acetone, is interpreted as protonated CH_3_N=CHOH and/or CH_2_=NCH_2_OH – the photo-oxidation product(s) resulting from OH addition to the π-system carbon atom, Scheme 1. The latter weak and noisy signal is understood as the corresponding two dehydration fragments (CH_3_N=CH^+^ and CH_2_=NCH_2_^+^). The *m*/*z* 58.033 is interpreted as protonated CH_2_=NCHO – the major photo-oxidation product following H-abstraction from the CH_2_ group in MMI. CH_2_=NCHO is also predicted as a minor product resulting from H-abstraction from the CH_3_-group (<15%). There is, however, no obvious ion signal from the major product following H-abstraction from the CH_3_-group, CHONCO at *m*/*z* 72.009, indicating that the yield of this route is either very small or that CHONCO reacts very fast with OH. A recent study of the CH_3_NCO + OH reaction shows CHONCO as the primary product,^59^ and a comparison of the published CH_3_NCO and CHONCO time profiles (Figure 7 in ref (59)) indicates that CHONCO reacts around 20 times faster with OH than the parent compound, *k*_OH+CH3NCO_ = 1.36 × 10^–13^ cm^3^ molecule^–1^ s^–1^ at 298 K.^59^ This places the OH-reactivity of CHONCO on the same scale as that of MMI, which, in turn, implies that CHONCO should be a reliable indicator of H-abstraction from the CH_3_-group. While the *m*/*z* 72.008 intensity is well below the 1% cutoff limit, it can safely be concluded that the CH_3_-abstraction route in the MMI + OH reactions amounts to <2%.

Because the FTIR spectra unambiguously show that TMT is not present in any significant amount in this experiment, the relative ion signal intensities between *m*/*z* 58.029 and the sum of 60.045 and 42.034 reflect the branching between H-abstraction from the =CH_2_ group and C-addition in the MMI + OH reaction.

The relative instrument sensitivity to CH_2_=NCH_2_OH, CH_3_N=CHOH, and CH_2_=NCHO essentially only depends the ion–molecule reaction rate coefficients, since the instrumental mass discrimination function is effectively the same for *m*/*z* 58.029 and 60.045 and since ionization in PTR-MS happens at the collisional rate.^60^ The ion–molecule reaction rate coefficients, in turn, can be quite precisely estimated from the calculated dipole moments and isotropic polarizabilities listed in Table S1.^30^ For *E*/*N* 107 Td, the following rate coefficients are calculated: *k*_CH2=NCH2OH+H3O+_ = 2.28, *k*_CH3N=CHOH+H3O+_ = 1.69, and *k*_CH2=NCHO+H3O+_ = 3.06 × 10^–9^ cm^3^ molecule^–1^ s^–1^ at a drift tube temperature of 100 °C. The *m*/*z* 58.029 and 60.045 ion signals are excellently correlated throughout the experiment, except in the short period when IPN was injected. An analysis of the time periods 12:30–13:30 and 13:45–16:00, based upon the above-mentioned ion–molecule reaction rate coefficients and a 90:10 ratio in the CH_3_N=CHOH : CH_2_=NCH_2_OH product distribution of the OH addition route, finds the branching ratio between H-abstraction from the CH_2_ group and C-addition to be 18:82 ± 3 (3σ-limit). Changing the theoretical value for the CH_3_N=CHOH:CH_2_=NCH_2_OH product distribution in the addition route from 90:10 to 70:30 or 100:0 only alters the derived branching within the estimated error limits.

### 3.3 Synthesis of Experimental and Theoretical Results

The present quantum chemistry calculations are not capable of narrowing the branching in the MMI + OH reaction better than 34–90% CH_2_–abstraction, 7–56% C-addition, and 1–12% CH_3_-abstraction, Scheme 1. In principle, the three routes can be discerned by PTR-MS as the major product of each route has a different mass. The PTR-MS results place a clear upper limit of 2% to the CH_3_-abstraction route and an 18:82 ± 3 ratio between CH_2_ abstraction and C-addition. The experimental value assumes (1) that no tautomerization of the MMI + OH reaction products occurs in the instrument inlet lines and detection system and (2) that the dehydration of protonated CH_3_N=CHOH and CH_2_=NCH_2_OH takes place.

The fragmentation of protonated CH_3_N=CHOH and CH_2_=NCH_2_OH was investigated in theoretical calculations showing that proton transfer selectively takes place at the OH-group and that CH_2_=NCH_2_OH_2_^+^ spontaneously ejects H_2_O, resulting in the [CH_2_=N=CH_2_]^+^ cation. The proton transfer in the CH_3_N=CHOH + H_3_O^+^ reaction is more complex, taking place via complex formation on the entrance side followed by competing H-migration and H_2_O ejection. There is a relatively low barrier of 27 kJ mol^–1^ between the H_3_O^+^ complex on the entrance side and the post transfer dimeric H_2_O complex on the exit side and a somewhat larger barrier of 67 kJ mol^–1^ to the H-migration route.
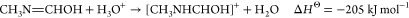
28a
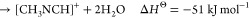
28b

The energetics of reaction 28 is illustrated in Figure S7 (the underlying quantum chemistry results are documented in Table S11). The branching in reaction 28 was investigated in master equation calculations based on the PES illustrated in Figure S7; the effective temperature in the PTR-ToF-MS drift tube being operated at *E*/*N* 88 Td (EUPHORE experiments) is ∼1000 K, whereas the 107 Td employed in the Oslo experiments corresponds to ∼1300 K. The calculations indicate the branching to be determined in part by thermodynamics, and predict a branching of 75:25 at 1000 K and 90:10 at 1300 K, which is consistent with higher relative ion signals of *m*/*z* 42.034 to 60.045 in the EUPHORE experiments, Figure 7, than in the Oslo experiments, Figure 9. The branching in the CH_3_N^•^CH_2_OH + O_2_ reaction (3) can be extracted from the observed relative intensities of the *m*/*z* 42.034 and 60.045 ion signals in the Oslo experiments when taking the calculated fragmentations of protonated CH_3_N=CHOH (10%) and CH_2_=NCH_2_OH (100%) into consideration. The average *m*/*z* 42.034 and 60.045 ion signal ratio 0.42 ± 0.11 corresponds to a branching (3a):(3b) = 22:78 (±10, 2σ). By providence, this compares well with the theoretical result 10:90.

Concerning CH_3_N=CHOH → CH_3_NHCHO tautomerization, the theoretical study located a barrier around 135 kJ mol^–1^ corresponding to a unimolecular rate coefficient around 1.4 × 10^–6^ s^–1^ at 298 K and 2.7 × 10^–4^ s^–1^ at 398 K. Consequently, CH_3_N=CHOH is not expected to tautomerize to any significant degree in the PTR inlet and detection system unless the process is surface catalyzed. In the hypothetical case of 100% tautomerization of CH_3_N=CHOH to CH_3_NHCHO, the instrument response factor for *m*/*z* 60.045 should then be based on *k*_CH3NHCHO+H3O+_ = 4.12 × 10^–9^ cm^3^ molecule^–1^ s^–1^ at 100 °C. This, in turn, would bring the estimated branching ratio between H-abstraction from the CH_2_ group and C-addition to be 34:66 ± 3 (3σ-limit). In any case, there is an obvious discord between theory and experiments with respect to the initial branching in the MMI + OH reaction.

**Figure 10:**
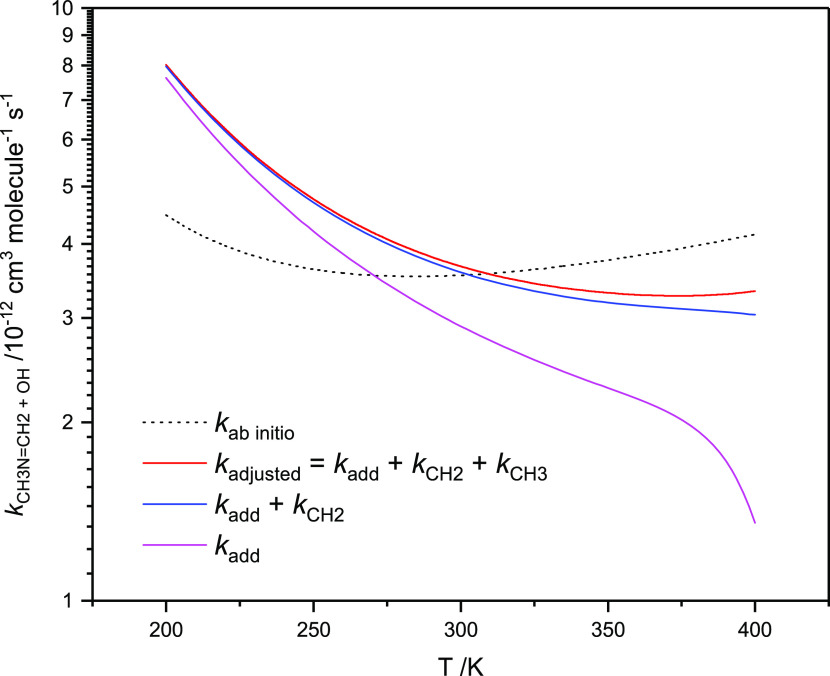
Cumulative plot of rate coefficients for the OH radical reaction with *N*-methyl methanimine calculated with Eckart tunneling, hindered internal rotations, scaled vibrational wavenumbers, and adjusted barrier heights to reproduce the observed branching in the reaction. Based on results from CCSD(T*)-F12a/aug-cc-pVTZ//M06-2X/aug-cc-pVTZ calculations.

**Figure 11:**
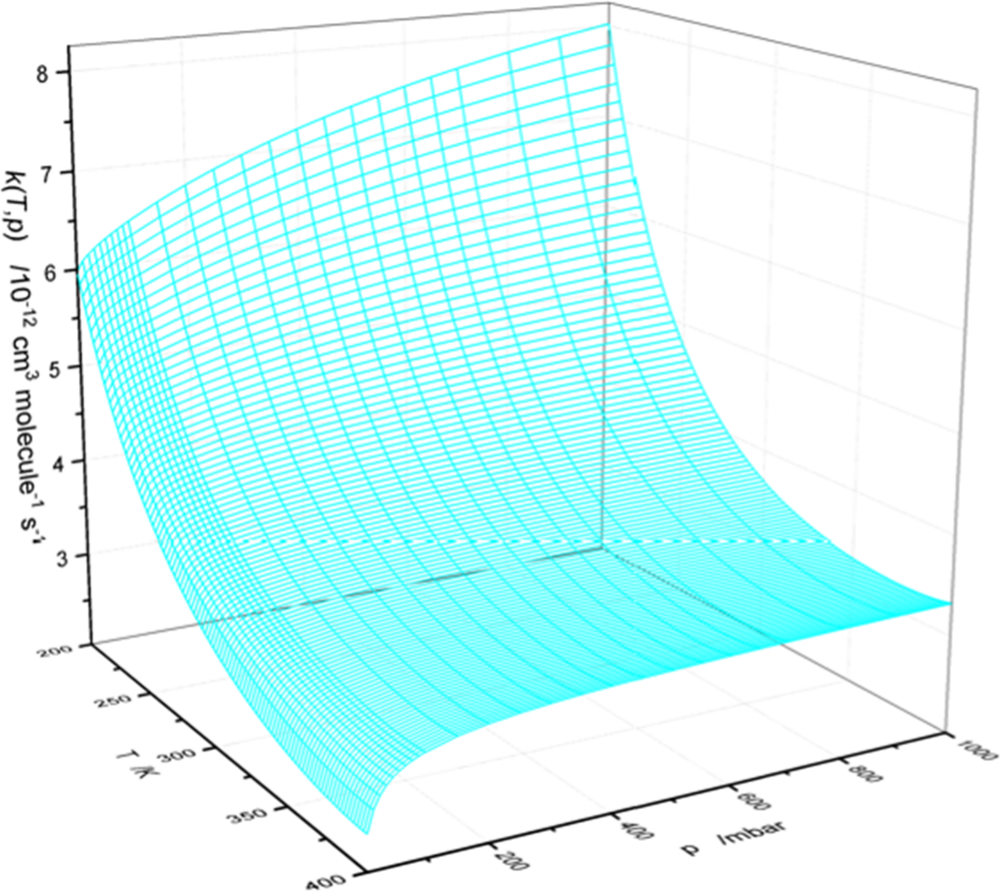
Rate coefficient for the CH_3_N=CH_2_ + OH reaction as a function of *p* and *T*. Results from MESMER calculations including Eckart tunneling and hindered internal rotations, based on CCSD(T*)-F12a/aug-cc-pVTZ//M06-2X/aug-cc-pVTZ calculations.

The sensitivity analysis of the quantum chemistry based kinetic model for the MMI + OH reaction shows that the reaction rate coefficient and the branching essentially only depend on the saddle point energies SP-1a (C-addition) and SP-1c (CH_2_-abstraction leading to *E*-CH_3_N=CH). We consider the calculated saddle point energies associated with uncertainties of ±4 kJ mol^–1^. It is, however, not possible to reproduce the observed branching by adjusting a single saddle point energy by only 4 kJ mol^–1^. As there is no unique solution to fitting the experimental branching by adjusting the saddle point energies, we therefore advocate a single correction as a first approach: –Δ*E* to the C-addition saddle point energy (SP-1a) and +Δ*E* to each of the three saddle points to H-abstraction (SP-1c, SP-1d, and SP-1e). Adjusting the saddle point energies as indicated above by Δ*E* = 3.15 kJ mol^–1^ changes the branching between reactions 1a–1e from 27:0:64:3:6 to 80:0:1:17:2 while leaving the calculated rate coefficient at 298 K essentially unchanged. Figure 10 compares the ab initio and the adjusted ab initio rate coefficients for the overall CH_3_N=CH_2_ + OH reaction as a function of temperature. The difference between the two predictions is surprisingly small—less than a factor of 2 for tropospheric conditions. The figure also illustrates the contribution from the addition and the CH_2_-abstraction routes to the total rate coefficient (the rate coefficients for the individual routes are documented in Table S12 for selected temperatures). The overall rate coefficient shows a moderate pressure dependency under tropospheric conditions (100–1000 mbar, 220–300 K) with a variation of ∼15% at 220 K, Figure 11. Discrete values of *k*(*p*,*T*) are collected in Table S13.

The temperature dependence of the rate coefficient at 1000 mbar can conveniently be parametrized according to the modified Arrhenius equation *k*(T) = 5.70 × 10^–14^ × (*T*/298 K)^3.18^ × exp(1245 K/*T*) cm^3^ molecule^–1^ s^–1^ with *k*(298 K) = 3.7 × 10^–12^ cm^3^ molecule^–1^ s^–1^. The rate coefficient at 298 K is comparable to that of the CH_2_=NH + OH reaction, calculated in a similar way (3 × 10^–12^ cm^3^ molecule^–1^ s^–1^),^20^ and it is almost an order of magnitude smaller than the recommended high-pressure value for the CH_3_CH=CH_2_ reaction with OH.^47^ In this context, it should be noted that the CH_3_CH=CH_2_ + OH reaction is entirely an addition reaction under atmospheric conditions, whereas the CH_3_N=CH_2_ + OH reaction—like the CH_2_=NH + OH reaction^20,21^—also proceeds via H–abstraction.

## 4 Conclusions

The atmospheric photo-oxidation of MMI (CH_3_N=CH_2_) has been detailed on the basis of quantum chemistry calculations showing CH_2_=NCHO and CH_3_N=CHOH and/or CH_2_=NCH_2_OH as the major products; N_2_O will not be formed in the atmospheric gas phase degradation, and there are no indications of nitrosamine and nitramine formation. The potential energy surface of the CH_3_N=CH_2_ + OH reaction was characterized in coupled cluster theory calculations, and master equation modeling reveals a minor pressure dependency and a negative temperature dependency of the reaction, with typical values of *k*_*OH*_ around 3.7 × 10^–12^ cm^3^ molecule^–1^ s^–1^ under tropospheric conditions. The MMI + Cl reaction^17^ and the MMI + O_3_ reaction as well as tropospheric photolysis are all found to be too slow to be of importance on a global scale. With a diurnal OH radical concentration of 10^6^ cm^–3^,^61^ the atmospheric lifetime of MMI with respect to reaction with OH will be around 2^1^/_2_ days. The night-time chemistry of MMI is likely dominated by the NO_3_ radical, and assuming that MMI follows the OH-NO_3_ reactivity correlation for either addition or abstraction,^49^ this places *k*_NO3+MMI_ in the range 4.4 × 10^–17^ to 1.1 × 10^–16^ cm^3^ molecule^–1^ s^–1^ at 298 K. Taking an average night-time NO_3_ concentration around 5 × 10^8^ cm^–3^,^49,62^ results in τ_NO3_ > ^1^/_2_ yr for MMI. That is, the NO_3_ radical is not expected to present any significant atmospheric sink for MMI.

Urban clouds, fog, and deliquescent particles are in general acidic, and considering the uptake coefficients for methylamines on 59–82 wt % sulfuric acid (γ ∼ 2 × 10^–2^)^63^ as the expected level for imine uptake on particles, in general, the aqueous particle uptake of MMI will be diffusion controlled under atmospheric conditions. MMI will consequently partition preferentially to the aqueous particle phase,^64^ and although atmospheric conditions are highly variable, hydrolysis to CH_2_O and CH_3_NH_2_ will be a dominating atmospheric removal of MMI.

The major MMI photo-oxidation products, CH_2_=NCHO and CH_3_N=CHOH and/or CH_2_=NCH_2_OH, are likewise expected to partition to the aqueous particle phase where hydrolysis will result in CH_2_O + NH_2_CHO and CH_3_NH_2_ + HCOOH or CH_2_O + NH_2_CH_2_OH.

## Supporting Information Available

Infrared spectrum of 1,3,5-trimethylhexahydro-1,3,5-triazine (Figure S1), infrared spectra of a trimerizing CH_3_N=CH_2_ sample (Figure S2), infrared spectrum of CH_3_N=CH_2_ (Figure S3), dipole moments and isotropic polarizabilities (Table S1), quantum chemistry calculation results for the CH_3_N=CH_2_ + OH reaction (Table S2), OH rotational potentials in the CH_3_N=CH_2_+OH reaction saddle points (Figure S4), sensitivity analysis of the CH_3_N=CH_2_ + OH reaction model (Table S3), quantum chemistry calculation results for CH_3_N^•^CH_2_OH formation and isomerization (Table S4), quantum chemistry calculation results for the CH_3_N^•^CH_2_OH + O_2_ reaction (Table S5), rotational potential of the CNOO^•^ moiety in the CH_3_N(OO^•^)CH_2_OH radical (Figure S5), quantum chemistry calculation results for the (*E*)–CH_3_NC^•^H + O_2_ reaction (Table S6), quantum chemistry calculation results for the CH_2_NC^•^H_2_ + O_2_ reaction (Table S7), relative energies of stationary points on the PES of the CH_2_=NCH_2_OO^•^ + NO reaction (Figure S6), quantum chemistry calculation results for the CH_2_NCH_2_OO^•^ + NO reaction (Table S8), G4 results for the CH_3_CH=CH_2_ + O_3_ and CH_3_N=CH_2_ + O_3_ reactions (Table S9), quantum chemistry calculation results for CH_3_NCH_2_ photolysis (Table S10), primary products in the OH initiated photo-oxidation of TMT under atmospheric conditions (Scheme S1), relative energies of stationary points on the PES of the CH_3_N=CCHOH + H_3_O^+^ reaction (Figure S7), quantum chemistry calculation results for the CH_3_N=CCHOH + H_3_O^+^ reaction (Table S11), calculated rate coefficients for the CH_3_N=CH_2_ + OH reaction (Table S12), rate coefficients for the CH_3_N=CH_2_ + OH reaction at discrete values of *p* and T (Table S13) (PDF)
