# Supplementary material for: Atmospheric Chemistry of N-Methylmethanimine (CH3N=CH2): A Theoretical and Experimental Study
Source: J Phys Chem A. 2022 May 11;126(20):3247–64. doi: 10.1021/acs.jpca.2c01925 (PMC9150125; doi:10.1021/acs.jpca.2c01925)
Supplement: Supplementary file 1 — jp2c01925_si_001.pdf [file jp2c01925_si_001.pdf]

Atmospheric Chemistry of *N*-Methylmethanimine ($CH_3N=CH_2$) – a Theoretical and Experimental Study

Supporting Information

Arne Joakim C. Bunkan,[1] Nina G. Reijrink,[1] Tomáš Mikoviny,[1] Markus Müller,[2¶] Claus J. Nielsen,[1] Liang Zhu[1] and Armin Wisthaler[1,2]

[1] Section of Environmental Sciences, Department of Chemistry, University of Oslo, P.O.Box. 1033 – Blindern 0315 Oslo, Norway.

[2] Institute for Ion Physics and Applied Physics, University of Innsbruck, 6020 Innsbruck, Austria.

Figure S1. Infrared spectrum of 1,3,5-trimethylhexahydro-1,3,5-triazine....2
Figure S2. Infrared spectra of a trimerizing $CH_3N=CH_2$ sample. 2
Figure S3. Infrared spectrum of $CH_3N=CH_2$. 3
Table S1. Dipole moments and isotropic polarizabilities. 3
Table S2. QCC results for the $CH_3N=CH_2$ + OH reaction 4
Figure S4. OH rotational potentials in the $CH_3N=CH_2$+OH reaction saddle points. 12
Table S3. Sensitivity analysis of the $CH_3N=CH_2$ + OH reaction model. 13
Table S4. QCC results for $CH_3\dot{N}CH_2OH$ formation and isomerization....13
Table S5. QCC results for $CH_3\dot{N}CH_2OH + O_2$....16
Figure S5. Rotational potential of the CNOȮ moiety in the $CH_3N(O\dot{O})CH_2OH$ radical. 19
Table S6. QCC results for (*E*)-$CH_3N\dot{C}H + O_2$. 20
Table S7. QCC results for $CH_2N\dot{C}H_2 + O_2$. 24
Figure S6. Relative energies of stationary points on the PES of the $CH_2=NCH_2O\dot{O}$ + NO reaction....29
Table S8. QCC results for $CH_2NCH_2O\dot{O}$ + NO. 29
Table S9. G4 results for $CH_3CH=CH_2 + O_3$ and $CH_3N=CH_2 + O_3$. 31
Table S10. QCC results for CH_3NCH_2 photolysis. 34
Scheme S1. Primary products in the OH initiated photo-oxidation of TMT under atmospheric conditions....36
Figure S7. Relative energies of stationary points on the PES of the $CH_3N=CCHOH + H_3O^+$ reaction. 37
Table S11. QCC results for $CH_3N=CCHOH + H_3O^+$. 37
Table S12. Calculated rate coefficients for the $CH_3N=CH_2$ + OH reaction. 38
Table S13. Rate coefficients for the $CH_3N=CH_2$ + OH reaction at discrete values of *p, T*....38

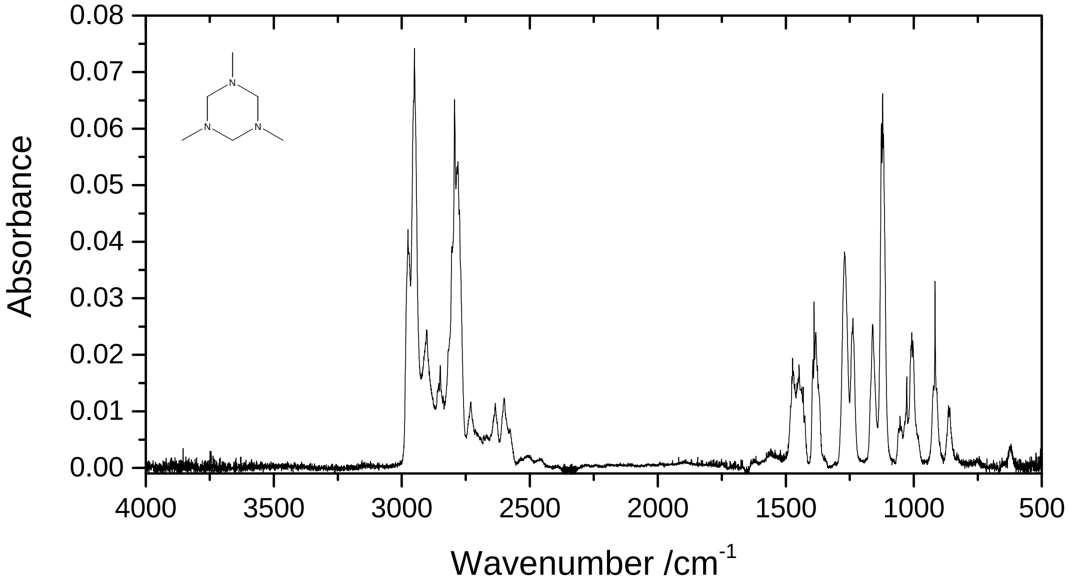

Figure S1. Infrared spectrum of 1,3,5-trimethylhexahydro-1,3,5-triazine.
Infrared absorption of 50 ppm·m 1,3,5- trimethylhexahydro-1,3,5-triazine (TMT) in the 4000 – 500 cm^{-1} region.

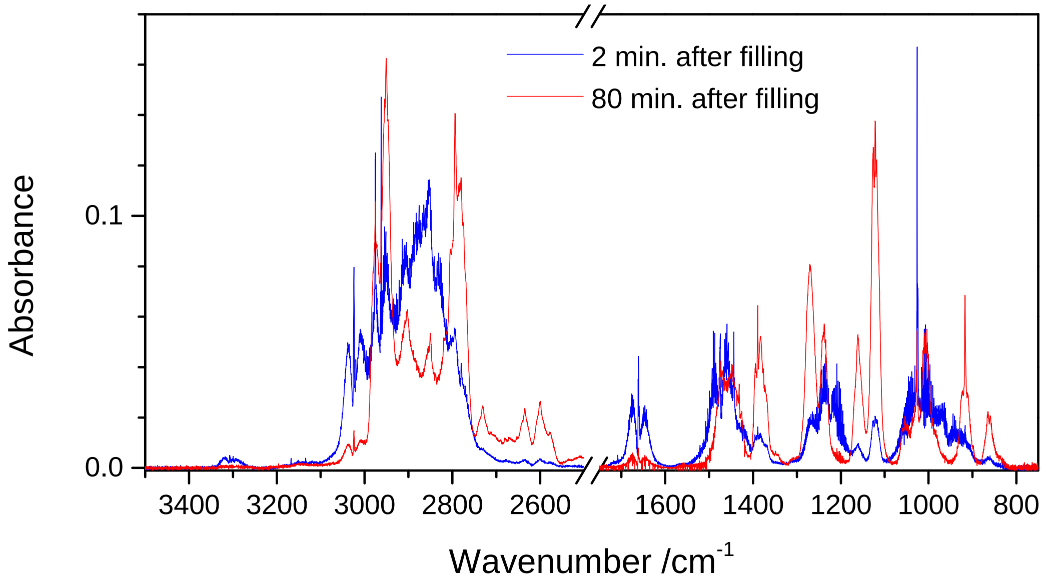

Figure S2. Infrared spectra of a trimerizing $CH_3N=CH_2$ sample.
Infrared absorption of a CH_3NCH_2 sample in the 3500 – 800 cm^{-1} region as a function of time. The partial pressure was ca. 1 mbar at start. Pathlength 10 cm, res. 0.5 cm^{-1}.

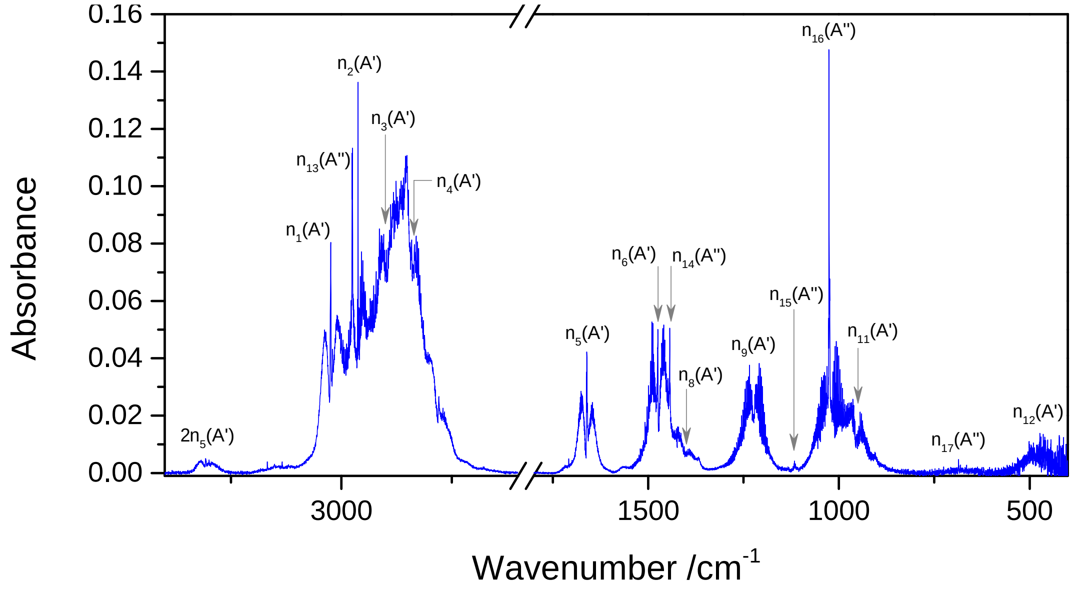

Figure S3. Infrared spectrum of $CH_3N{=}CH_2$.
Infrared absorption spectrum of a ~310 ppm·m $CH_3N{=}CH_2$ in the 3400 – 400 cm^{-1} region obtained by spectral subtraction. Pathlength 10 cm, resolution 0.5 cm^{-1}.

Table S1. Dipole moments and isotropic polarizabilities.
Dipole moments (μ /Debye) and isotropic polarizabilities (α /Å^3) obtained in B3LYP/aug-cc-pVTZ and M06-2X/aug-cc-pVTZ calculations.

Molecule	B3LYP		M06-2X	
	μ	α	μ	α
OH	1.650	1.113	1.692	1.063
$CH_3N{=}CH_2$	1.515	5.469	1.584	5.239
CH_2O	2.388	2.676	2.464	2.569
CH_3NH_2	1.287	3.904	1.319	3.756
$CH_2{=}NH$	2.012	3.425	2.050	3.425
HCN	3.041	2.536	3.078	2.480
$CH_2{=}NCH_2OH$	1.806	6.197	1.543	5.779
$CH_2{=}NCHO$	3.031	5.579	2.605	5.301
CH_3NHCHO	4.306	6.002	4.313	5.780
CH_3NCO	3.019	3.425	3.158	5.205
CH_3NCHOH	0.852	6.228	0.915	5.948
CH_3NHNO_2	4.458	6.335	4.446	6.091
$(CH_3)_2CO$	3.079	6.338	3.100	6.115

Table S2. QCC results for the $CH_3N=CH_2$ + OH reaction

A. Results from CCSD(T*)-F12a/aug-cc-pVTZ//M06-2X/aug-cc-pVTZ calculations.

Energies (/Hartree) of species included in Figure 1 in the main text and energy differences (/kJ mol^{-1}) between stationary points on the PES of the $CH_3N=CH_2$ + OH reaction.

Species	M06-2X/aTZ		CCSD(T*)-F12a/aTZ	
	E_{Elec}	E_{ZPE}	E_{Elec}	$\Delta E_{v=0}$
$CH_3N=CH_2$	-133.924870	0.068750	-133.765173	
OH	-75.733789	0.008530	-75.670902	
Sum reactants	-209.658659	0.077280	-209.436075	0.0
PRE	-209.671062	0.080322	-209.447905	-23.1
SP 1a	-209.661260	0.080402	-209.439991	-2.1
$CH_3\dot{N}CH_2OH$	-209.712877	0.083592	-209.486619	-116.1
SP 1b	-209.652606	0.080634	-209.431322	21.3
$\dot{C}H_3N(OH)CH_2$	-209.664665	0.082935	-209.439174	6.7
SP 1c	-209.659115	0.076569	-209.435570	-0.5
(E)-$CH_3N\dot{C}H$•H_2O	-209.699966	0.079143	-209.477366	-103.5
(E)-$CH_3N\dot{C}H$	-133.262196	0.055290	-133.099766	
H_2O	-76.425276	0.021593	-76.371156	
Sum products	-209.687472	0.076883	-209.470922	-92.5
SP 1d	-209.655663	0.075385	-209.431502	7.0
(Z)-CH_3NCH•H_2O	-209.694149	0.079121	-209.471756	-88.8
(Z)-$CH_3N\dot{C}H$	-133.255130	0.054793	-133.092022	
H_2O	-76.425276	0.021593	-76.371156	
Sum products	-209.680406	0.076386	-209.463177	-73.5
SP 1e	-209.655694	0.075730	-209.432214	6.1
$CH_2\dot{N}CH_2$•H_2O	-209.715437	0.078710	-209.493046	-145.8
$CH_2\dot{N}CH_2$	-133.275071	0.054059	-133.111798	
H_2O	-76.425276	0.021593	-76.371156	
Sum products	-209.700347	0.075652	-209.482954	-127.4

B. Results from CCSD(T*)-F12a/aug-cc-pVTZ//MP2/aug-cc-pVTZ calculations.

Energies (/Hartree) of species included in Figure 1 in the main text and energy differences (/kJ mol^{-1}) between stationary points on the PES of the $CH_3N{=}CH_2$ + OH reaction.

Species	MP2/aTZ		CCSD(T*)-F12a/aTZ	
	E_{Elec}	E_{ZPE}	E_{Elec}	$\Delta E_{v=0}$
$CH_3N{=}CH_2$	-133.666019	0.068684	-133.765427	
OH	-75.626337	0.008646	-75.670689	
Sum reactants	-209.292356	0.077330	-209.436116	0.0
PRE [a]	-209.304981	0.087011	-209.449052	-8.5
SP 1a	-209.281758	0.081709	-209.439254	3.3
$CH_3\dot{N}CH_2OH$	-209.343388	0.083994	-209.486707	-115.3
SP 1b	-209.269333	0.081919	-209.428453	32.2
$\dot{C}H_3N(OH)CH_2$	-209.297681	0.083098	-209.439345	6.7
SP 1c	-209.281246	0.077837	-209.436283	0.9
(E)-$CH_3N\dot{C}H{\bullet}H_2O$	-209.338470	0.081071	-209.475381	-93.3
(E)-$CH_3N\dot{C}H$	-132.999475	0.056842	-133.098039	
H_2O	-76.328992	0.021395	-76.370991	
Sum products	-209.328467	0.078237	-209.469031	-84.0
SP 1d	-209.278200	0.076817	-209.430825	12.5
(Z)-$CH_3NCH{\bullet}H_2O$	-209.328447	0.080181	-209.470279	-82.2
(Z)-$CH_3N\dot{C}H$	-132.990709	0.056143	-133.090131	
H_2O	-76.328992	0.021395	-76.370991	
Sum products	-209.319701	0.077538	-209.461123	-65.1
SP 1e	-209.280520	0.078458	-209.430249	18.4
$CH_2\dot{N}CH_2{\bullet}H_2O$	-209.341881	0.079361	-209.492699	-143.2
$CH_2\dot{N}CH_2$	-133.002106	0.054861	-133.111462	
H_2O	-76.328992	0.021395	-76.370991	
Sum products	-133.002106	0.054861	-133.111462	-124.5

[a] Erroneous vibrational frequencies.

Table S2A, continued.

T_1 and D_1 diagnostic values, vibrational frequencies (cm^{-1}), Rotational constants (GHz) and Cartesian coordinates (A) of the species listed above. Results from CCSD(T*)-F12a/aug-cc-pVTZ//M06-2X/aug-cc-pVTZ calculations.

$CH_3N{=}CH_2$

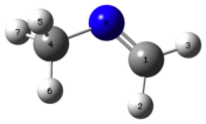

T_1= 0.0182, D_1= 0.0664

$\tilde{\nu}$: 191.0, 326.9, 393.0, 598.4, 627.1, 982.6, 1044.2, 1140.9, 1257.8, 1309.3, 1446.7, 1471.6, 1491.6, 1506.4, 1541.7, 3022.0, 3100.7, 3147.7, 3172.6, 3286.4, 3619.8

B: 42.7480952 9.9050061 8.5808449

C	-1.174295	0.182239	0.000000
H	-1.140148	1.277953	-0.000001
H	-2.152857	-0.290246	0.000000
C	1.143064	0.133755	0.000000
H	1.705616	-0.186179	0.876736
H	1.062666	1.226585	-0.000001
H	1.705616	-0.186181	-0.876735
N	-0.141930	-0.533985	0.000000

OH

T_1= 0.0080, D_1= 0.0145

$\tilde{\nu}$: 3769.6

B: 0.0000000, 564.2863408, 564.2863408

O	0.000000	0.000000	0.107992
H	0.000000	0.000000	-0.863937

PRE

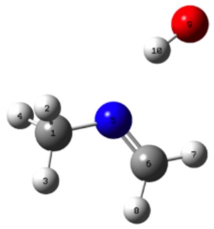

T_1= 0.0110, D_1= 0.0339

$\tilde{\nu}$: 21.0, 107.6, 169.7, 212.4, 500.1, 568.3, 673.5, 705.1, 995.7, 1093.1, 1131.3, 1151.3, 1264.2, 1442.5, 1485.1, 1487.2, 1518.3, 1783.4, 3035.9, 3049.8 3122.3, 3137.8, 3167.5, 3434.3

B: 12.7550435, 3.3817846, 2.7177143

C	-1.608355	-0.763771	0.000000
H	-1.632705	-1.408291	-0.877770
H	-2.488731	-0.114517	0.000000
H	-1.632705	-1.408292	0.877770
N	-0.364317	-0.021220	0.000000
C	-0.407994	1.235379	0.000000
H	0.522428	1.798372	0.000000
H	-1.347221	1.795867	0.000000
O	2.467743	-0.360959	0.000000
H	1.485301	-0.456579	0.000000

SP-1a

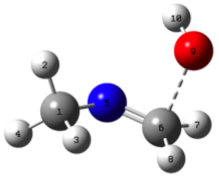

T_1= 0.0296, D_1= 0.1283

$\tilde{\nu}$: -517.8, 145.6, 151.0, 272.8, 298.8, 499.7, 748.6, 822.6, 985.0, 1062.4, 1123.5, 1125.4, 1249.1, 1428.9, 1459.0, 1475.3, 1509.7, 1620.5, 3029.9, 3073.1, 3111.0, 3119.5, 3204.6, 3776.7

B: 13.3202936 5.2457625 4.3238506

C	1.484673	-0.469363	0.145244
H	1.408573	-1.471196	-0.278427
H	1.322694	-0.524546	1.225841
H	2.494868	-0.112099	-0.059310
N	0.545790	0.397155	-0.531219
C	-0.356722	0.952248	0.193405
H	-1.038536	1.656031	-0.268766
H	-0.354127	0.892933	1.283102
O	-1.615938	-0.658616	0.080909
H	-1.494204	-0.849586	-0.863073

$CH_3\dot{N}CH_2OH$

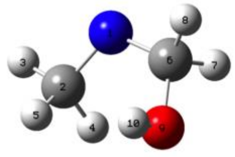

T_1= 0.0147, D_1= 0.0505

$\tilde{\nu}$: 87.5, 151.4, 302.6, 307.5, 606.2, 901.7, 937.7, 977.7, 1085.1, 1122.2, 1197.6, 1229.9, 1369.8, 1392.9, 1407.9, 1469.1, 1478.7, 1487.0, 3011.3, 3015.5, 3055.4, 3066.9, 3148.7, 3882.2

B: 15.6426794 6.1757250 4.8076529

N	0.662324	0.757629	-0.192228
C	1.436484	-0.426088	0.052882
H	2.492707	-0.205183	-0.075501
H	1.258063	-0.808082	1.065128
H	1.155540	-1.239630	-0.624317
C	-0.732941	0.616004	0.092383
H	-0.881666	0.971718	1.119872
H	-1.284026	1.289283	-0.569558
O	-1.275264	-0.687543	0.052639
H	-1.396027	-0.950659	-0.862723

SP-1b

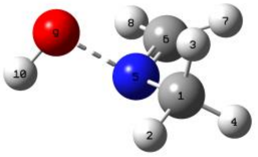

T_1= 0.0355, D_1= 0.1286

$\tilde{\nu}$: -553.1, 103.4, 244.7, 315.2, 384.4, 431.0, 629.9, 882.8, 959.2, 996.3, 1101.9, 1132.3, 1223.1, 1431.8, 1433.8, 1482.2, 1505.3, 1556.4, 3071.7, 3104.3, 3147.3, 3167.9, 3240.3, 3849.0

B: 9.8099925 7.9767273 4.7776066

C	0.808180	-1.097257	0.037672
H	0.361946	-1.927275	-0.501907
H	0.708279	-1.279082	1.106917
H	1.860899	-1.013461	-0.228887
N	0.124919	0.132457	-0.330180
C	0.612035	1.263507	0.047093
H	1.525137	1.317608	0.635173
H	0.068961	2.167963	-0.187474
O	-1.500961	-0.115880	0.204250
H	-1.913255	-0.263414	-0.655153

$CH_3N(OH)\dot{C}H_2$

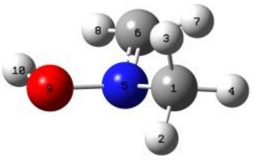

T_1= 0.0192, D_1= 0.0754

$\tilde{\nu}$: 224.0, 274.9, 332.2, 366.9, 436.9, 477.3, 588.5, 863.2, 1010.8, 1080.3, 1141.8, 1258.1, 1337.4, 1385.3, 1438.2, 1474.0, 1493.0, 1516.7, 3046.6, 3132.6, 3164.6, 3192.1, 3313.3, 3855.5

B: 9.9967591 9.4610271 5.2397316

C	-0.838462	1.114532	0.092773
H	-0.056287	0.045454	-0.292126
H	-0.718071	-1.698564	-0.626188
H	-0.396615	2.089526	-0.033251
N	-1.905297	0.976372	0.039432
C	-0.599746	-1.176001	0.172711
H	1.353499	0.108159	0.055029
H	1.880250	-0.704850	-0.438611
O	1.747955	1.057192	-0.301485
H	1.493527	0.034008	1.136480

SP-1c

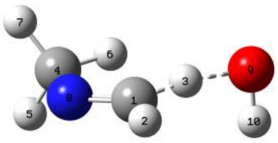

T_1= 0.0306, D_1= 0.1390

$\tilde{\nu}$: -553.1, 103.4, 244.7, 315.2, 384.4, 431.0, 629.9, 882.8, 959.2, 996.3, 1101.9, 1132.3, 1223.1, 1431.8, 1433.8, 1482.2, 1505.3, 1556.4, 3071.7, 3104.3, 3147.3, 3167.9, 3240.3, 3849.0

B: 9.8099925 7.9767273 4.7776066

C	-0.105052	1.103055	-0.034531
H	-0.040245	2.187929	0.024361
H	0.877316	0.546681	-0.192257
C	-1.242129	-0.937175	-0.016234
H	-1.697626	-1.318176	0.896840
H	-0.249342	-1.377124	-0.151021
H	-1.888562	-1.221709	-0.845153
N	-1.195433	0.509566	0.058518
O	2.153992	-0.373334	-0.089603
H	2.217635	-0.393177	0.879016

(E)-$CH_3N\dot{C}H{\bullet}H_2O$

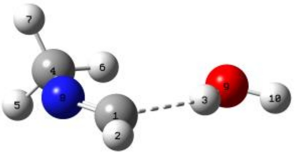

T_1= 0.0208, D_1= 0.0855

$\tilde{\nu}$: 46.5, 74.2, 123.7, 154.2, 175.1, 348.1, 363.5, 509.2, 736.2, 934.8, 1012.1, 1123.9, 1125.9, 1430.6, 1482.7, 1494.1, 1631.9, 1905.0, 3008.5, 3056.9, 3149.0, 3160.7, 3743.1, 3940.3

B: 12.9606628, 3.4746300, 2.7869660

C	-0.541443	1.265155	0.000622
H	-0.852932	2.313848	-0.002622
H	1.527824	0.478207	-0.004228
C	-1.015974	-1.110776	0.002433
H	-1.486052	-1.550967	0.879235
H	0.055975	-1.306549	0.011942
H	-1.470581	-1.551945	-0.882011
N	-1.297571	0.312446	-0.000887
O	2.201200	-0.216504	-0.009768
H	3.043664	0.236041	0.063701

(E)-$CH_3N\dot{C}H$

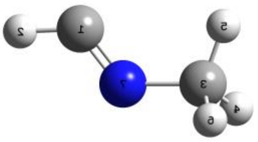

T_1= 0.0250, D_1= 0.0881

$\tilde{\nu}$: 173.7, 326.5, 723.6, 940.4, 997.1, 1115.3, 1122.4, 1427.4, 1489.2, 1498.3, 1923.2, 2979.0, 3045.9, 3135.3, 3153.9

B: 64.0448325, 11.0283982, 9.9891898

C	-1.249159	0.273251	0.000000
H	-2.263938	-0.134295	0.000003
C	1.142143	0.099718	0.000000
H	1.650168	-0.288765	0.880327
H	1.179888	1.189239	-0.000047
H	1.650199	-0.288845	-0.880273
N	-0.224889	-0.387879	-0.000001

SP-1d

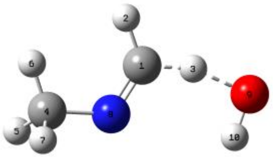

T_1= 0.0293, D_1= 0.1309

$\tilde{\nu}$: -877.6, 90.7, 91.6, 199.4, 228.7, 345.0, 666.3, 762.3, 999.7, 1045.9, 1078.8, 1135.8, 1187.8, 1296.3, 1437.8, 1485.9, 1502.4, 1617.0, 1818.4, 3023.7, 3035.7, 3124.7, 3142.5, 3773.9

B: 33.8021897, 2.7380665, 2.5729934

C	-0.118153	0.652852	0.000002
H	0.201895	1.702886	0.000005
H	-1.269682	0.491918	0.000002
C	2.068503	-0.164350	-0.000001
H	2.478337	-0.660438	0.878250
H	2.369009	0.887412	0.000002
H	2.478337	-0.660432	-0.878257
N	0.626188	-0.339786	-0.000001
O	-2.524540	-0.157656	0.000001
H	-2.146991	-1.052607	-0.000002

(Z)-CH_3NCH•H_2O

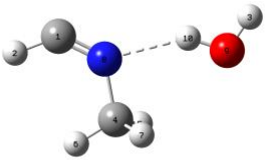

T_1= 0.0211, D_1= 0.0859

$\tilde{\nu}$: 46.5, 74.2, 123.7, 154.2, 175.1, 348.1, 363.5, 509.2, 736.2, 934.8, 1012.1, 1123.9, 1125.9, 1430.6, 1482.7, 1494.1, 1631.9, 1905.0, 3008.5, 3056.9, 3149.0, 3160.7, 3743.1, 3940.3

B: 12.9606628, 3.4746300, 2.7869660

C	-1.673503	-0.962885	-0.000004
H	-2.734209	-0.683787	-0.000041
H	2.861739	-0.912029	0.000025
C	-0.551593	1.159364	0.000000
H	0.015826	1.460497	0.877640
H	-1.535415	1.631056	-0.000051
H	0.015904	1.460469	-0.877600
N	-0.654014	-0.302419	0.000017
O	2.242971	-0.180102	-0.000010
H	1.361064	-0.577325	0.000011

(Z)-CH_3NCH

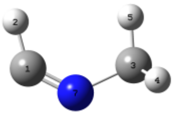

T_1= 0.0260, D_1= 0.0920

$\tilde{\nu}$: 173.7, 326.5, 723.6, 940.4, 997.1, 1115.3, 1122.4, 1427.4, 1489.2, 1498.3, 1923.2, 2979.0, 3045.9, 3135.3, 3153.9

B: 64.0448325, 11.0283982, 9.9891898

C	-1.298349	0.074703	0.000000
H	-1.581734	1.136370	0.000000
C	1.098497	0.148918	0.000000
H	1.642802	-0.186215	0.880257
H	1.029949	1.239223	0.000001
H	1.642801	-0.186214	-0.880258
N	-0.219244	-0.477841	0.000000

SP-1e

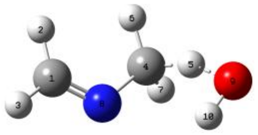

T_1= 0.0252, D_1= 0.1072

$\tilde{\nu}$: -1380.3, 298.8, 474.7, 506.8, 614.1, 645.5, 761.8, 897.6, 937.4, 1097.1, 1107.6, 1155.9, 1237.6, 1321.9, 1367.4, 1415.2, 1499.1, 1530.1, 1646.0, 2970.8, 3026.6, 3170.4, 3206.0, 3812.8

B: 14.0251559, 7.0574469, 5.3923688

C	-1.773232	-0.448214	0.211304
H	-2.055741	0.080332	1.127629
H	-2.399425	-1.275404	-0.109646
C	0.051099	0.946723	-0.013596
H	1.113717	0.504128	0.196488
H	-0.278147	1.412801	0.920570
H	0.217685	1.675173	-0.804116
N	-0.765420	-0.132893	-0.476053
O	2.172888	-0.425820	0.155209
H	1.709538	-1.051273	-0.426471

$CH_2N\dot{C}H_2$•H_2O

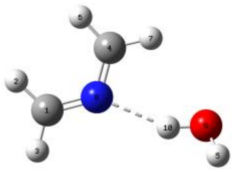

T_1= 0.0126, D_1= 0.0342

$\tilde{\nu}$: 62.9, 104.4, 111.4, 165.4, 371.9, 426.2, 496.1, 602.8, 617.0, 868.6, 901.9, 1093.0, 1182.5, 1238.1, 1311.5, 1498.7, 1559.3, 1640.5, 3085.5, 3096.7, 3243.0, 3245.3, 3686.3, 3940.

B: 14.3107295, 3.3221010, 2.7062479

C	-1.714427	-0.767758	-0.011190
H	-2.660471	-0.229118	-0.035506
H	-1.721564	-1.847508	-0.014167
C	-0.531442	1.178431	0.018757
H	2.805999	-0.793749	0.498458
H	-1.445467	1.769914	-0.008122
H	0.429807	1.671982	0.042117
N	-0.560330	-0.136446	0.019585
O	2.324371	-0.201279	-0.080403
H	1.394252	-0.470206	-0.022057

$CH_2N\dot{C}H_2$

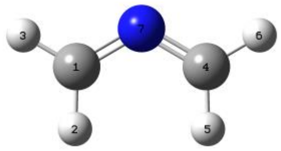

T_1= 0.0117, D_1= 0.0279
$\tilde{\nu}$: 392.2, 487.7, 598.2, 868.5, 891.8, 1089.7, 1182.3, 1235.1, 1301.0, 1499.6, 1560.3, 3069.7, 3081.2, 3234.6, 3237.2
B: 66.4471597, 12.0073210, 10.1696215

C	-1.132649	-0.155689	0.000021
H	-1.160878	-1.245939	0.000018
H	-2.063587	0.392461	0.000054
C	1.132648	-0.155689	-0.000022
H	1.160878	-1.245940	-0.000019
H	2.063588	0.392460	-0.000053
N	0.000001	0.510746	0.000000

Table S2B, continued.
T_1 and D_1 diagnostic values, vibrational frequencies (cm^{-1}), Rotational constants (GHz) and Cartesian coordinates (A) of the species listed above. Results from CCSD(T*)-F12a/aug-cc-pVTZ//MP2/aug-cc-pVTZ calculations.

$CH_3N{=}CH_2$

T_1= 0.0120, D_1= 0.0340
$\tilde{\nu}$: 209.0, 490.2, 696.9, 982.2, 1056.2, 1127.3, 1149.0, 1239.4, 1443.8, 1485.5, 1498.4, 1523.0, 1691.1, 3022.3, 3041.7, 3132.6, 3160.3, 3200.0
B: 52.3695486 10.7573322 9.4421698

C	-1.176262	0.187690	0.000000
H	-1.130026	1.281877	-0.000001
H	-2.154539	-0.280728	0.000000
C	1.142068	0.137811	0.000000
H	1.706492	-0.173257	0.877849
H	1.044750	1.229661	-0.000001
H	1.706492	-0.173258	-0.877848
N	-0.138287	-0.548186	0.000000

OH
T_1= 0.0080, D_1= 0.0145
$\tilde{\nu}$: 3795.1, B: 0.0000000, 0.0000005 67.1986547

O	0.000000	0.000000	0.107715
H	0.000000	0.000000	-0.861716

PRE

T_1= 0.0110, D_1= 0.0339
$\tilde{\nu}$: 39.8, 87.2, 176.3, 221.9, 501.1, 553.3, 724.5, 751.1, 1005.2, 1132.8,
1134.7, 1165.3, 1262.6, 1449.9, 1499.5, 1509.0, 1530.2, 2992.1, 3038.5, 3143.6, 3167.8, 3195.7, 3466.7, 4071.4
B: 11.3482038 3.4241286 2.6737401

C	-1.402383	-0.948646	0.000000
H	-1.286321	-1.580795	-0.878548
H	-2.402725	-0.504570	0.000001
H	-1.286320	-1.580796	0.878547
N	-0.348679	0.052079	0.000000
C	-0.688844	1.264474	0.000000
H	0.081674	2.028667	-0.000001
H	-1.734523	1.585687	0.000001
O	2.511278	-0.247948	0.000000
H	1.526107	-0.224130	-0.000002

SP-1a

T_1= 0.0302, D_1= 0.1309
$\tilde{\nu}$: -617.2, 162.5, 177.1, 308.8, 332.2, 500.0, 775.6, 876.4, 991.1, 1133.9, 1141.3, 1161.6, 1257.9, 1444.4, 1489.1, 1493.3, 1525.3, 1703.2, 3031.9, 3082.9, 3133.0, 3152.1, 3234.2, 3758.4
B: 13.6780166 5.3864603 4.4567400

C	1.482186	-0.444282	0.145739
H	1.434728	-1.454126	-0.259745
H	1.321545	-0.486447	1.227868
H	2.480411	-0.058442	-0.060932
N	0.513729	0.391876	-0.535921
C	-0.375323	0.930646	0.188151
H	-1.076343	1.619484	-0.265298
H	-0.360665	0.880111	1.278897
O	-1.578329	-0.656199	0.083240
H	-1.410330	-0.912305	-0.838609

$CH_3\dot{N}CH_2OH$

T_1= 0.0146, D_1= 0.0497
$\tilde{\nu}$: 93.8, 153.3, 302.1, 313.2, 599.0, 900.9, 941.0, 982.3, 1054.8, 1122.8,
1203.7, 1236.5, 1373.5, 1396.4, 1420.2, 1485.1, 1492.1, 1506.2, 3030.1, 3040.0, 3097.5, 3107.6, 3176.4, 3840.4
B: 15.5938120 6.1450081 4.7964992

N	0.668481	0.755009	-0.206568
C	1.435108	-0.433999	0.052825
H	2.488230	-0.230800	-0.117481
H	1.287706	-0.767439	1.086046
H	1.117984	-1.266457	-0.580433
C	-0.723791	0.619276	0.104433
H	-0.842582	0.946946	1.143540
H	-1.282251	1.308541	-0.531442
O	-1.282593	-0.686479	0.047311
H	-1.455609	-0.895689	-0.876284

SP-1b

T_1= 0.0327, D_1= 0.1223
$\tilde{\nu}$: -997.4, 109.6, 247.7, 331.1, 397.1, 467.1, 721.8, 947.3, 981.6, 1062.4, 1121.6, 1167.0, 1231.1, 1434.6, 1469.4, 1497.3, 1523.7, 1622.2, 3064.7, 3112.2, 3163.9, 3204.7, 3263.8, 3816.3
B: 10.0700000 0.0004507 7.9463027

C	0.496825	1.293498	0.030371
N	0.099910	0.140281	-0.273256
H	-1.870788	-0.480176	-0.661045
H	1.439610	1.447916	0.550747
H	-0.135293	2.140907	-0.192521
O	-1.497686	-0.223394	0.191925
C	0.920130	-1.020757	0.031067
H	1.058313	-1.606389	-0.874875
H	1.891787	-0.710595	0.420179
H	0.396761	-1.622924	0.766276

$CH_3N(OH)\dot{C}H_2$

T_1= 0.0192, D_1= 0.0747
$\tilde{\nu}$: 236.5, 283.4, 349.0, 377.4, 427.2, 471.7, 618.8, 832.8, 994.8, 1073.4, 1144.8, 1241.6, 1328.5, 1372.6, 1442.5, 1484.8, 1504.2, 1528.3, 3059.0, 3157.1, 3189.9, 3211.7, 3343.8, 3801.9
B: 9.9600000 0.0014040 9.4193702

C	-0.784739	1.148327	0.099661
N	-0.051662	0.052363	-0.313005
H	-0.801807	-1.656457	-0.633692
H	-0.308997	2.104330	-0.044327
H	-1.855893	1.050516	0.063716
O	-0.651874	-1.153665	0.176537
C	1.355231	0.050804	0.060908
H	1.857004	-0.771076	-0.442445
H	1.789963	0.991590	-0.269821
H	1.473410	-0.050904	1.141891

SP-1c

T_1= 0.0415, D_1= 0.1938
$\tilde{\nu}$: -1738.1, 110.6, 116.0, 149.3, 236.5, 485.3, 597.1, 824.9, 845.4, 987.4, 1123.5, 1168.2, 1208.5, 1329.0, 1427.5, 1449.3, 1508.4, 1518.6, 2725.8, 3048.3, 3153.4, 3172.9, 3197.8, 3782.8
B: 12.2524775 4.0410465 3.1341470

C	-0.002071	1.043910	-0.036815
H	0.201580	2.111238	0.031478
H	0.957232	0.376035	-0.198530
C	-1.324513	-0.869725	-0.014806
H	-1.806279	-1.199616	0.903463
H	-0.380849	-1.403881	-0.154866
H	-1.999782	-1.088269	-0.839314
N	-1.131078	0.570047	0.056140
O	2.095669	-0.421113	-0.088172
H	2.139795	-0.462040	0.879886

(E)-$CH_3N\dot{C}H{\bullet}H_2O$

T_1= 0.0182, D_1= 0.0698
$\tilde{\nu}$: 64.7, 110.9, 112.8, 169.3, 175.5, 395.6, 503.5, 509.0, 654.1, 932.0,
958.3, 1166.4, 1201.0, 1455.9, 1509.1, 1513.9, 1651.3, 2479.0, 3068.9, 3084.5,
3183.4, 3192.1, 3598.2, 3897.0
B: 9.8336956 4.0355853 2.9134131

C	-0.461734	1.309103	-0.000365
H	-0.917401	2.309068	0.000699
H	1.454636	0.463299	0.000241
C	-1.042699	-1.110378	-0.000392
H	-1.525195	-1.513892	0.885650
H	0.015665	-1.368026	-0.003209
H	-1.529671	-1.513501	-0.884159
N	-1.191760	0.326445	0.000405
O	2.113463	-0.255963	0.001296
H	2.963182	0.193289	-0.007880

(E)-$CH_3N\dot{C}H$

T_1= 0.0237, D_1= 0.0818
$\tilde{\nu}$: 155.6, 349.4, 609.4, 940.0, 1020.7, 1163.4, 1166.0, 1445.9, 1511.0, 1516.0,
2558.1, 3074.4, 3079.2, 3176.8, 3185.0
B: 88.9392821 10.1338123 9.6419188

C	-1.274006	0.263114	0.000000
H	-2.249800	-0.238007	0.000020
C	1.158303	0.081272	0.000001
H	1.649551	-0.320388	0.882476
H	1.229269	1.168081	-0.000352
H	1.649741	-0.320976	-0.882099
N	-0.226363	-0.336432	-0.000007

SP-1d

T_1= 0.0348, D_1= 0.1584
$\tilde{\nu}$: -2168.9, 32.3, 96.0, 205.2, 234.4, 351.8, 675.7, 802.0, 859.0, 1020.0, 1136.3, 1141.1, 1161.7, 1226.9, 1445.5, 1501.7, 1516.5, 1542.1, 2573.6, 3037.9, 3061.2, 3148.5, 3176.3, 3773.2
B: 36.6019505 2.7616462 2.6092367

C	-0.128782	0.596980	-0.000019
H	0.137546	1.663890	0.000072
H	-1.302890	0.408629	-0.000078
C	2.068541	-0.129276	0.000024
H	2.497561	-0.604507	0.879500
H	2.320733	0.935772	0.000092
H	2.497635	-0.604412	-0.879466
N	0.629305	-0.363849	-0.000049
O	-2.500997	-0.126701	0.000012
H	-2.186302	-1.045043	0.000091

(Z)-$CH_3NCH{\bullet}H_2O$

T_1= 0.020, D_1= 0.0839
$\tilde{\nu}$: 34.1, 52.8, 95.8, 144.4, 173.6, 278.9, 348.7, 511.3, 708.3, 935.3, 979.6, 1116.6, 1151.0, 1444.5, 1503.5, 1514.7, 1642.4, 2502.7, 3019.6, 3070.0, 3176.1, 3196.0, 3687.7, 3907.5
B: 11.3900000 0.0074952 3.5594693

C	-1.479685	-1.111745	0.000008
H	-2.580663	-1.092058	0.000134
H	2.830485	-0.903164	-0.000009
C	-0.707978	1.203175	0.000008
H	-0.188743	1.572041	0.880107
H	-1.743131	1.546622	0.000149
H	-0.188964	1.572107	-0.880195
N	-0.645854	-0.254936	-0.000055
O	2.269164	-0.123286	0.000019
H	1.364659	-0.473284	-0.000046

(Z)-CH_3NCH

T_1= 0.0252, D_1= 0.0880
$\tilde{\nu}$: 165.2, 259.2, 663.6, 899.6, 976.6, 1112.6, 1151.6, 1444.2, 1507.7, 1516.8, 2550.0, 2985.6, 3060.1, 3163.0, 3188.3
B: 74.6218119 10.4907823 9.7539061

C	-1.326590	0.036117	-0.000030
H	-1.706105	1.073460	-0.000226
C	1.128463	0.131340	0.000000
H	1.653885	-0.225684	0.881740
H	1.109663	1.223075	-0.000319
H	1.654015	-0.226199	-0.881453
N	-0.217529	-0.407056	0.000062

SP-1e

T_1= 0.0272, D_1= 0.1166
$\tilde{\nu}$: -2012.0, 81.0, 138.3, 259.6, 361.1, 451.6, 663.4, 814.4, 917.8, 1012.6, 1131.5, 1223.2, 1231.6, 1255.9, 1297.6, 1474.3, 1530.9, 1559.5, 2723.9, 3057.0, 3097.0, 3186.5, 3207.7, 3763.0
B: 17.4812585 3.6212835 3.2346800

C	-1.784483	-0.386945	0.200401
H	-2.113024	0.261022	1.021808
H	-2.426170	-1.223847	-0.060472
C	0.083653	0.920372	-0.044160
H	1.181453	0.490051	0.138858
H	-0.208968	1.405219	0.891936
H	0.190352	1.623747	-0.865825
N	-0.734196	-0.205630	-0.428997
O	2.135856	-0.403370	0.156381
H	1.633863	-1.090388	-0.311822

$CH_2N\dot{C}H_2 \bullet H_2O$	C	-1.580256	-0.883601	-0.012689
	H	-2.593326	-0.487269	-0.047788
T_1= 0.0127, D_1= 0.0348	H	-1.429791	-1.951325	-0.011071
$\tilde{\nu}$: 41.9, 104.4, 106.6, 163.8, 397.1, 496.4, 496.5, 635.7, 674.0,	C	-0.699023	1.200570	0.017745
914.9, 955.3, 1030.9, 1189.9, 1210.0, 1347.4, 1515.8, 1587.3,	H	2.751433	-0.648726	0.626376
1657.8, 3111.0, 3120.0, 3281.6, 3285.9, 3605.3, 3905.8	H	-1.689064	1.651760	-0.018665
B: 13.1300000 0.0001445 3.3673212	H	0.175998	1.831459	0.045130
	N	-0.534474	-0.095748	0.024555
	O	2.350659	-0.161020	-0.097570
	H	1.396471	-0.339318	-0.015647
$CH_2N\dot{C}H_2$	C	-1.125332	-0.155193	0.000025
	H	-1.150511	-1.245020	0.000024
T_1= 0.0116, D_1= 0.0274	H	-2.055779	0.390661	0.000049
$\tilde{\nu}$: 478.6, 488.2, 631.3, 914.5, 942.1, 1037.1, 1191.2, 1209.0,	C	1.125330	-0.155194	-0.000025
1337.1, 1516.3,	H	1.150506	-1.245022	-0.000024
1587.0, 3094.7, 3103.0, 3274.0, 3276.9	H	2.055779	0.390657	-0.000048
B: 66.6208389 12.1545155 10.2791542	N	0.000003	0.510149	0.000000

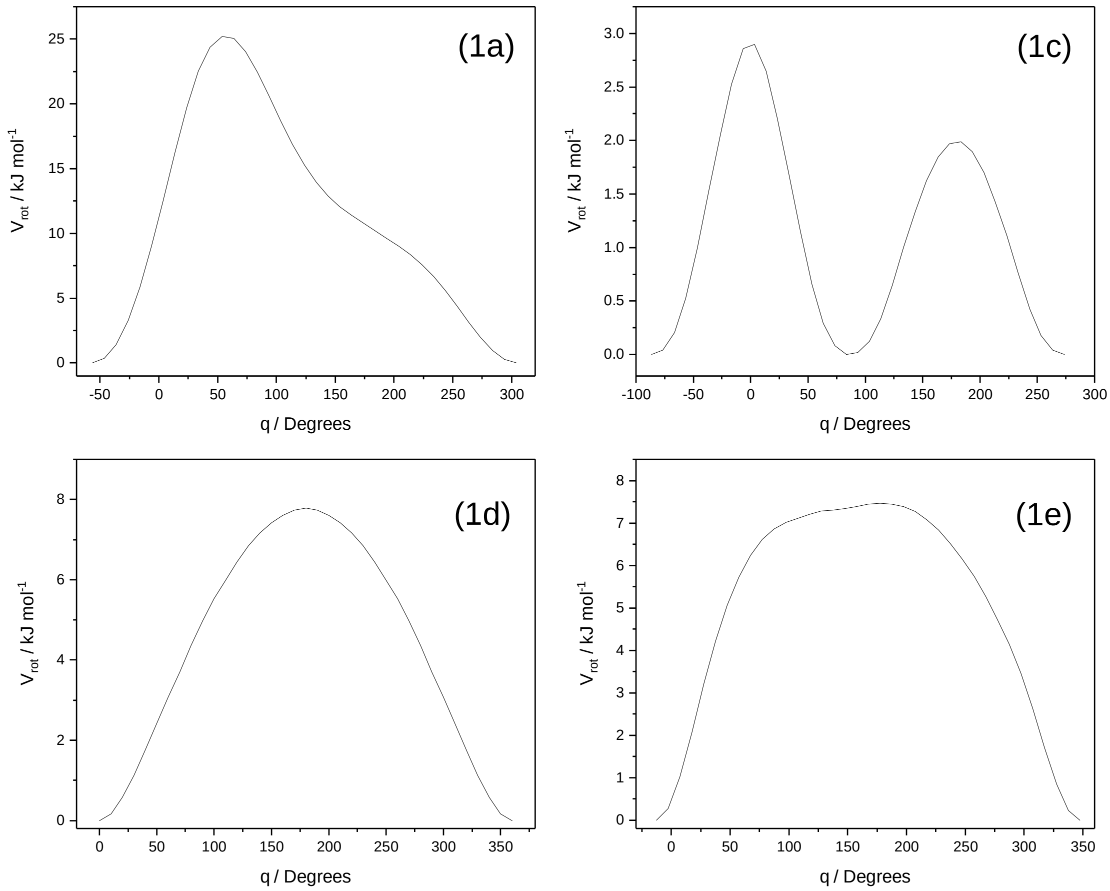

Figure S4. OH rotational potentials in the $CH_3N{=}CH_2$+OH reaction saddle points.
V_{rot} (/kJ mol^{-1}) for the OH fragment in the saddle points (1a), (1c), (1d) and (1e). Results from M06-2X/aug-cc-pVTZ calculations.

Table S3. Sensitivity analysis of the $CH_3N{=}CH_2$ + OH reaction model.
Sensitivity of the rate coefficient (k_{tot} / 10^{-12} cm^3 $molecule^{-1}$ s^{-1}) at 298 K and the branching ratios (Γ_i / %) to variation in the energies of stationary points (ΔE_{SP} /kJ mol^{-1}) on the potential energy surface of the $CH_3N{=}CH_2$ + OH reaction.

	Δk	$\Delta\Gamma_{1a}$	$\Delta\Gamma_{1b}$	$\Delta\Gamma_{1c}$	$\Delta\Gamma_{1d}$	$\Delta\Gamma_{1e}$
ΔE_{PRE}	0.00	0.0	0.0	0.0	0.0	0.0
ΔE_{SP1a}	-0.34	-7.6	0.0	6.7	0.3	0.6
ΔE_{SP1b}	0.00	0.0	0.0	0.0	0.0	0.0
ΔE_{SP1c}	-0.84	6.8	0.0	-9.2	0.8	1.6
ΔE_{SP1d}	-0.04	0.3	0.0	0.8	-1.2	0.1
ΔE_{SP1e}	-0.08	0.7	0.0	1.6	0.1	-2.3

Table S4. QCC results for $CH_3\dot{N}CH_2OH$ formation and isomerization.
Energies (/Hartree) of species involved in the $CH_3\dot{N}CH_2OH$ radical formation and isomerization, and energy differences (/kJ mol^{-1}) between stationary points on the PES.
Results from CCSD(T*)-F12a/aug-cc-pVTZ//M06-2X /aug-cc-pVTZ calculations.

Species	M06-2X/aTZ		CCSD(T*)-F12a/aTZ	
	E_{Elec}	E_{ZPE}	E_{Elec}	$\Delta E_{v=0}$
$CH_3N{=}CH_2$	-133.924870	0.068750	-133.765120	
OH	-75.733810	0.008588	-75.670688	
Sum reactants	-209.658680	0.077338	-209.435808	0.0
PRE	-209.671063	0.080322	-209.448849	-26.4
SP-1a	-209.661260	0.080402	-209.440006	-3.0
$CH_3\dot{N}CH_2OH$	-209.712877	0.083592	-209.486602	-116.9
SP-2	-209.662170	0.080186	-209.438081	1.5
$CH_3NHCH_2\dot{O}$	-209.698964	0.083731	-209.471438	-76.8
SP-3	-209.676217	0.081192	-209.452805	-34.5
$CH_3\dot{N}H$	-95.177174	0.049277	-95.070095	
CH_2O	-114.498984	0.027101	-114.383833	
Sum products	-209.676158	0.076378	-209.453928	-50.1

Table S4, continued.

T_1 and D_1 diagnostic values, vibrational frequencies (cm^{-1}), Rotational constants (GHz) and Cartesian coordinates (A) of the species listed above.

Species	
$CH_3N{=}CH_2$	See Table S2
OH	
PRE	
SP-1a	
$CH_3\dot{N}CH_2OH$	

SP-2

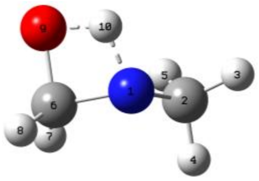

T_1= 0.0223, D_1= 0.0953

$\tilde{\nu}$: -1836.2, 199.8, 228.2, 422.9, 759.4, 888.5, 1005.0, 1020.8, 1099.9, 1126.4, 1167.4, 1207.9, 1236.5, 1323.7, 1424.0, 1488.2, 1495.1, 1547.7, 2173.4, 3034.3, 3058.4, 3096.7, 3118.9, 3155.2

B: 18.4222914 5.7528136 5.3019371

N	0.378046	0.141533	-0.630721
C	1.534715	-0.128863	0.206425
H	2.242169	-0.750039	-0.336688
H	2.014686	0.818045	0.463157
H	1.250200	-0.631077	1.135824
C	-0.770168	0.651922	0.126410
H	-0.515592	1.034215	1.117196
H	-1.358977	1.368853	-0.445428
O	-1.310174	-0.640800	0.147077
H	-0.384701	-0.842686	-0.692644

$CH_3NHCH_2\dot{O}$

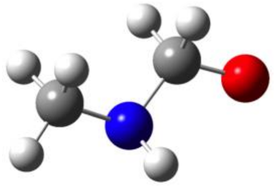

T_1= 0.0179, D_1= 0.0710

$\tilde{\nu}$: 153.7, 210.8, 340.5, 569.2, 689.0, 801.7, 979.6, 1020.4, 1070.4, 1154.1, 1200.8, 1282.8, 1344.8, 1393.9, 1458.9, 1487.9, 1498.2, 1526.6, 2970.4, 3006.2, 3031.8, 3099.3, 3145.5, 3564.5

B: 17.3090599 5.5332119 4.7655103

C	0.653249	0.401523	0.013770
H	0.581277	1.191653	-0.752673
H	0.625909	0.957154	0.983371
O	1.875691	-0.158273	-0.007106
N	-0.444542	-0.517141	-0.145742
H	-0.328519	-1.291390	0.495815
C	-1.738075	0.123407	0.021308
H	-1.874612	0.868141	-0.763934
H	-2.527493	-0.616752	-0.085278
H	-1.861341	0.627783	0.989275

SP-3

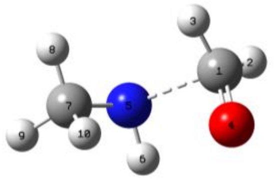

T_1= 0.0253, D_1= 0.1034

$\tilde{\nu}$: -365.8, 104.3, 158.3, 243.7, 381.6, 733.5, 808.5, 1010.6, 1024.0, 1087.5, 1197.4, 1245.1, 1339.3, 1407.6, 1483.7, 1485.4, 1500.3, 1650.0, 2967.9, 3036.9, 3040.3, 3093.5, 3141.4, 3453.7

B: 16.9634112 4.7082125 4.3209005

C	-1.127620	-0.378981	0.365597
H	-1.531081	-1.349755	0.049654
H	-0.779215	-0.342013	1.409450
O	-1.394248	0.655762	-0.250121
N	0.669915	-0.652894	-0.339905
H	0.511075	-0.459406	-1.330990
C	1.587751	0.331722	0.185578
H	1.782736	0.129202	1.236654
H	2.528923	0.285323	-0.368638
H	1.191352	1.344367	0.077121

$CH_3\dot{N}H$

T_1= 0.0145, D_1= 0.0403

$\tilde{\nu}$: 259.9, 960.2, 1009.3, 1070.7, 1333.4, 1400.3, 1488.6, 1489.6, 3000.3, 3043.0, 3137.0, 3439.6

B: 125.6400000 0.0027852 25.5126022

C	-0.628935	-0.013638	0.000000
H	-0.961760	-0.581323	0.877486
H	-0.961760	-0.581323	-0.877486
H	-1.123283	0.955157	0.000000
N	0.800017	0.150782	0.000000
H	1.210735	-0.785552	0.000000

CH_2O

T_1= 0.0154, D_1= 0.0447
$\tilde{\nu}$: 1213.6, 1273.5, 1539.9, 1869.1, 2945.5, 3015.7
B: 284.7518606 39.4556659 34.6539588

C	0.000000	0.000000	-0.525505
H	0.000000	0.938356	-1.105549
H	0.000000	-0.938356	-1.105549
O	0.000000	0.000000	0.670516

Table S5. QCC results for $CH_3\dot{N}CH_2OH + O_2$.
Energies (/Hartree) of species included in Figure 2 in the main text and energy differences (/kJ mol^{-1}) between stationary points on the PES of the reactions of the $CH_3\dot{N}CH_2OH + O_2$ reaction. Results from CCSD(T*)-F12a/aug-cc-pVTZ//M06-2X /aug-cc-pVTZ calculations.

Species	M06-2X/aTZ		CCSD(T*)-F12a/aTZ	
	E_{Elec}	E_{ZPE}	E_{Elec}	$\Delta E_{v=0}$
$CH_3\dot{N}CH_2OH$	-209.712877	0.083592	-209.486619	
O_2	-150.324795	0.003997	-150.191373	
Sum reactants	-360.037672	0.087589	-359.677991	0.0
$CH_3N(O\dot{O})CH_2OH$	-360.056895	0.094321	-359.698536	-36.3
SP-5a	-360.018495	0.086543	-359.671940	13.1
$CH_2{=}NCH_2OH{\bullet}HO_2$	-360.086844	0.092347	-359.730708	-125.9
$CH_2{=}NCH_2OH$	-209.155531	0.074719	-208.928768	
HO_2	-150.908095	0.0145781	-150.778910	
Sum products	-360.063626	0.089297	-359.707678	-73.5
SP-5b	-360.020658	0.085508	-359.672167	9.8
$CH_3N{=}CHOH{\bullet}HO_2$	-360.103782	0.091641	-359.746463	-169.1
$CH_3N{=}CHOH$	-209.177289	0.074670	-208.949441	
HO_2	-150.908095	0.014578	-150.778910	
Sum products	-360.085384	0.089248	-359.728351	-127.7
SP-6	-209.119566	0.069567	-208.893182	
HO_2	-150.908095	0.014578	-150.778910	
Sum species	-360.027661	0.084145	-359.672092	6.4
CH_3NHCHO	-209.193145	0.074761	-208.965593	
HO_2	-150.908095	0.014578	-150.778910	
Sum species	-360.101240	0.089339	-359.744503	-170.0

Table S5, continued.

T_1 and D_1 diagnostic values, vibrational frequencies (cm^{-1}), Rotational constants (GHz) and Cartesian coordinates (A) of the species listed above.

Species

$CH_3\dot{N}CH_2OH$ — See Table S3

O_2

T_1= 0.0078, D_1= 0.0144

$\tilde{\nu}$: 1754.5, B: 0.0000000 44.6355338 44.6355338

O	0.000000	0.000000	0.594925
O	0.000000	0.000000	-0.594925

$CH_3N(O\dot{O})CH_2OH$

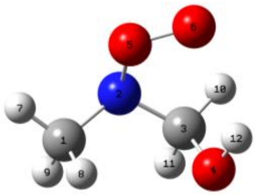

T_1= 0.0336, D_1= 0.1838

$\tilde{\nu}$: 106.7, 172.0, 229.9, 273.8, 357.1, 411.2, 449.8, 517.9, 611.3, 802.7, 937.3, 1001.3, 1125.1, 1154.6, 1176.0, 1231.6, 1304.4, 1330.3, 1397.2, 1435.8, 1461.0, 1488.9, 1494.5, 1509.5, 3018.9, 3035.7, 3137.5, 3141.5, 3177.4, 3858.1

B: 4.5749633 3.6597509 2.1878057

C	1.625968	0.728324	-0.301974
N	0.465417	0.421011	0.524961
C	0.158798	-0.990266	0.650634
O	-0.035866	-1.654136	-0.567637
O	-0.617668	1.142190	-0.086485
O	-1.741117	0.528951	-0.078445
H	1.708582	1.808333	-0.391454
H	1.561002	0.271635	-1.290757
H	2.502733	0.354380	0.221700
H	-0.699662	-1.091689	1.309657
H	1.035582	-1.423188	1.127740
H	-0.917544	-1.430934	-0.883038

SP-5a

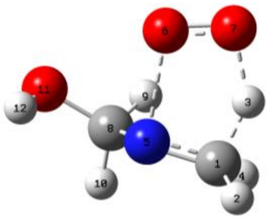

T_1= 0.0324, D_1= 0.1705

$\tilde{\nu}$: -1692.4, 46.2, 127.5, 150.8, 202.2, 305.4, 384.6, 495.9, 570.4, 601.6, 656.1, 942.8, 1024.1, 1153.2, 1166.3, 1196.5, 1229.8, 1301.4, 1371.8, 1391.0, 1431.7, 1486.0, 1501.1, 1556.0, 1594.1, 2979.3, 3031.5, 3064.1, 3154.6, 3872.0

B: 5.1300000 0.0047904 2.7419134

C	0.786960	1.468156	-0.056250
H	1.298845	2.062612	-0.808673
H	1.656394	0.587325	0.211725
H	0.651724	1.950515	0.915695
N	-0.173673	0.655624	-0.516042
O	0.790698	-1.138808	-0.188145
O	1.876617	-0.767031	0.246092
C	-1.249020	0.367330	0.404664
H	-0.886870	0.136697	1.411745
H	-1.847513	1.287642	0.454971
O	-2.014388	-0.715792	-0.019063
H	-2.107931	-0.654028	-0.974726

CH_2=NCH_2OH•HO_2

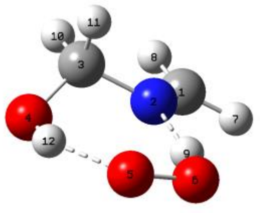

T_1= 0.0257, D_1= 0.1366

$\tilde{\nu}$: 57.4, 93.3, 135.5, 146.2, 194.9, 282.5, 442.0, 518.5, 631.0, 729.1, 873.8, 971.6, 1070.9, 1109.5, 1166.4, 1224.9, 1297.6, 1306.4, 1410.6, 1452.5, 1505.0, 1517.1, 1662.8, 1788.6, 2856.6, 3037.0, 3071.9, 3092.3, 3186.0, 3703.6

B: 4.4462311 2.3505580 1.6180418

C	1.886433	-1.219418	-0.165098
N	0.852515	-0.539495	0.057860
C	0.996342	0.870595	0.431498
O	0.263874	1.674034	-0.429139
O	-2.151959	0.183806	0.058007
O	-1.764607	-1.060354	0.003213
H	1.795183	-2.265752	-0.441029
H	2.889853	-0.794271	-0.093396
H	-0.749434	-1.009647	-0.021519
H	2.040938	1.184427	0.371129
H	0.649883	0.952619	1.466577
H	-0.669144	1.422133	-0.341827

CH_2=NCH_2OH

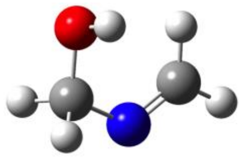

T_1= 0.0122, D_1= 0.0337

$\tilde{\nu}$: 225.4, 340.1, 348.5, 644.0, 690.4, 967.8, 1022.7, 1096.0, 1101.2, 1234.9, 1252.6, 1382.1, 1406.5, 1487.7, 1501.6, 1774.8, 3064.4, 3080.0, 3121.4, 3178.3, 3877.2

B: 18.5080192   6.4959234   5.0313707

N	0.796151	0.628577	-0.047277
C	1.411813	-0.466334	0.020695
H	2.497208	-0.457344	-0.019689
H	0.910776	-1.429409	0.129793
C	-0.647912	0.635770	0.012708
H	-0.926742	1.185670	0.911043
H	-0.997480	1.204767	-0.851870
O	-1.291493	-0.612600	0.092326
H	-1.308285	-1.019537	-0.777362

HO_2

T_1= 0.0358, D_1= 0.1228

$\tilde{\nu}$: 1252.8, 1459.4, 3686.8

B: 628.5431720 34.6992321 32.8838526

H	-0.880747	-0.865418	0.000000
O	0.055047	0.708193	0.000000
O	0.055047	-0.600015	0.000000

SP-5b

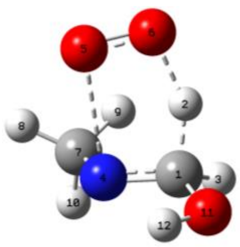

T_1= 0.0342, D_1= 0.1716

$\tilde{\nu}$: -2013.6, 114.2, 134.5, 149.0, 175.8, 264.3, 297.6, 350.9, 493.0, 562.0, 595.2, 1003.7, 1049.1, 1115.2, 1144.7, 1152.9, 1232.6, 1308.4, 1332.5, 1406.7, 1426.1, 1469.8, 1503.1, 1526.8, 1607.5, 3019.0, 3036.0, 3077.1, 3146.4, 3839.2

B: 3.9612754   2.9581231   1.9264630

C	0.991608	-0.058659	0.376158
H	-0.011026	-0.718295	0.722131
H	1.315975	0.305599	1.358455
N	0.404829	0.781636	-0.474642
O	-1.645952	-0.535187	-0.512662
O	-1.340072	-1.209835	0.455993
C	-0.306091	1.879523	0.136990
H	-1.163831	2.146864	-0.475098
H	-0.641462	1.666150	1.157524
H	0.366009	2.742261	0.172284
O	1.909056	-0.954298	-0.142241
H	1.803178	-0.944656	-1.100415

CH_3N=CHOH•HO_2

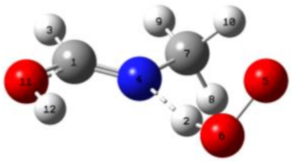

T_1= 0.0245, D_1= 0.1267

$\tilde{\nu}$: 19.7, 50.7, 81.9, 129.3, 203.7, 255.4, 287.0, 380.1, 580.1, 606.1, 839.8, 1022.4, 1039.9, 1151.2, 1163.1, 1219.4, 1282.0, 1357.5, 1409.8, 1452.4, 1485.6, 1516.9, 1621.0, 1802.0, 2999.6, 3049.4, 3126.9, 3139.7, 3143.0, 3809.8

B: 4.5921085   1.8478502   1.3940465

C	1.663979	-0.018339	-0.118128
H	-0.918462	-0.451182	0.454998
H	2.502922	0.616473	-0.400024
N	0.512980	0.402022	0.156909
O	-2.445167	-0.193753	-0.522846
O	-1.855534	-0.820622	0.463422
C	0.246518	1.828969	0.082332
H	-0.098125	2.180268	1.053767
H	1.128075	2.403432	-0.212011
H	-0.554395	1.997569	-0.635323
O	2.007211	-1.308599	-0.099986
H	1.234055	-1.840700	0.130289

CH_3N=CHOH

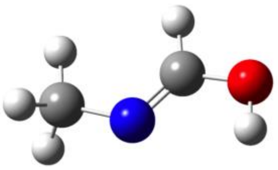

T_1= 0.0131, D_1= 0.0405

$\tilde{\nu}$: 197.3, 293.1, 334.9, 600.6, 609.5, 1013.7, 1031.6, 1145.4, 1155.3, 1208.2, 1344.1, 1401.9, 1451.2, 1489.0, 1514.5, 1805.3, 3024.8, 3106.9, 3115.8, 3125.0, 3808.2

N	-0.417080	-0.467527	-0.000002
C	-1.770316	0.047815	0.000001
H	-2.298450	-0.323019	-0.877860
H	-2.298434	-0.322982	0.877887
H	-1.815611	1.142152	-0.000022
C	0.525214	0.356344	-0.000001
H	0.423664	1.442809	-0.000002
O	1.817758	-0.013894	0.000001
H	1.836940	-0.980075	0.000002

B: 47.1700000 0.0016778 4.3915595

SP-6

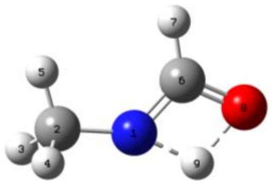

T_1= 0.0166, D_1= 0.0621
$\tilde{\nu}$: -1959.1, 152.0, 200.0, 341.5, 735.5, 1047.0, 1078.3, 1130.3, 1143.5, 1197.7, 1230.1, 1449.4, 1487.5, 1489.1, 1527.9, 1698.5, 2153.2, 3055.4, 3124.3, 3125.8, 3169.0
B: 43.5847891 4.6530078 4.3168672

N	0.347069	-0.311448	0.000000
C	1.755214	0.002632	0.000000
H	2.237227	-0.417074	0.882264
H	2.237227	-0.417074	-0.882264
H	1.910794	1.082494	0.000000
C	-0.664402	0.491408	0.000000
H	-0.643690	1.579240	0.000000
O	-1.745887	-0.193883	0.000000
H	-0.748818	-1.060621	0.000000

CH_3NHCHO

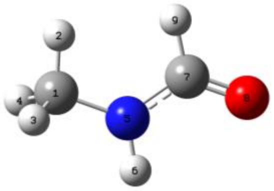

T_1= 0.0146, D_1= 0.0524
$\tilde{\nu}$: 97.4, 198.1, 352.8, 607.6, 622.1, 1022.5, 1056.9, 1155.9, 1181.0, 1312.2, 1404.0, 1472.9, 1492.5, 1497.4, 1543.3, 1822.4, 2993.1, 3063.4, 3121.0, 3148.6, 3624.6
B: 44.9521223 4.4096249 4.1194372

6	1.335159	1.120859	0.000000
1	2.050026	0.300045	0.000000
1	1.515813	1.728224	0.886651
1	1.515813	1.728224	-0.886651
7	0.000000	0.569313	0.000000
1	-0.795602	1.188740	0.000000
6	-0.286058	-0.753121	0.000000
8	-1.400230	-1.219506	0.000000
1	0.621183	-1.380803	0.000000

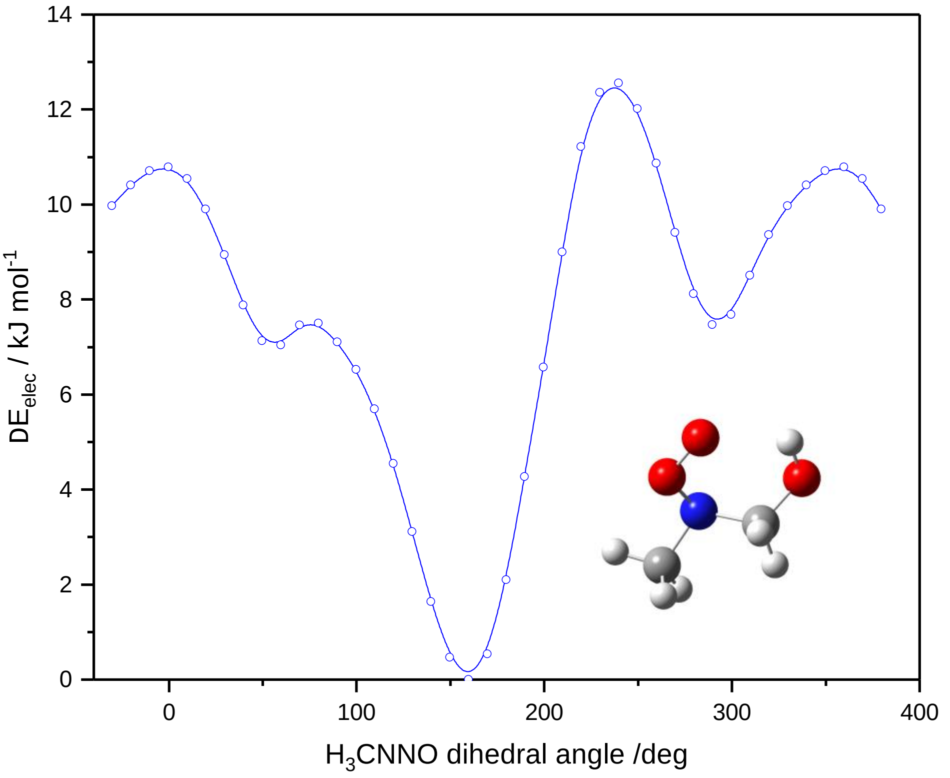

Figure S5. Rotational potential of the CNOȮ moiety in the $CH_3N(O\dot{O})CH_2OH$ radical. Results from M06-2X/aug-cc-pVTZ calculations.

Table S6. QCC results for (*E*)-$CH_3N\dot{C}H$ + O_2.

Energies (/Hartree) of species included in Figure 4 in the main text and energy differences (/kJ mol^{-1}) between stationary points on the PES of the $CH_3N\dot{C}H$ + O_2 reaction. Results from CCSD(T*)-F12a/aug-cc-pVTZ//M06-2X /aug-cc-pVTZ calculations.

	M06-2X/aTZ		CCSD(T*)-F12a/aTZ	
Species	E_{Elec}	E_{ZPE}	E_{Elec}	$\Delta E_{v=0}$
(*E*)-$CH_3N\dot{C}H$	-133.262196	0.055290	-133.099757	
O_2	-150.324815	0.004005	-150.191136	
Sum reactants	-283.587011	0.059295	-283.290894	0.0
PRE-10a	-283.589887	0.060565	-283.293178	-2.3
SP-10a	-283.581641	0.058797	-283.2919954	-3.8
$CH_3N{\equiv}C{\bullet}HO_2$	-283.634505	0.062492	-283.337893	-114.6
$CH_3N{\equiv}C$	-132.711702	0.045666	-132.544192	
HO_2	-150.908095	0.014578	-150.778909	
Sum products	-283.619796	0.060244	-283.323101	-81.7
$CH_3NCHO\dot{O}_{syn}$	-283.650514	0.065848	-283.353681	-147.3
SP-sa	-283.641539	0.065066	-283.345061	-126.7
$CH_3NCHO\dot{O}_{anti}$	-283.644784	0.065559	-283.347758	-132.5
SP-11b	-283.617421	0.060360	-283.322190	-79.2
$\dot{C}H_2N{=}CHOOH$	-283.641141	0.063108	-283.343974	-129.0
SP-12	-283.638296	0.061049	-283.344908	-136.9
$CH_2{=}NCHO$	-207.949556	0.050390	-207.719699	
OH	-75.733810	0.008588	-75.670688	
Sum products	-283.683367	0.058978	-283.390387	-261.7

Table S6, continued.

T_1 and D_1 diagnostic values, vibrational frequencies (cm^{-1}), Rotational constants (GHz) and Cartesian coordinates (A) of the species listed above.

Species
(*E*)-$CH_3N{=}\dot{C}H$ and OH, see Table S3. O_2 and HO_2, see Table S5.

PRE-10a

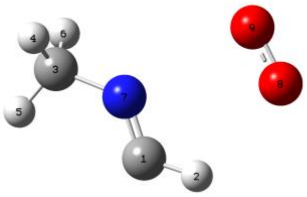

T_1= 0.0584, D_1= 0.3064

$\tilde{\nu}$: 63.6, 67.4, 75.3, 122.2, 155.8, 165.3, 394.7, 695.6, 941.0, 1060.9, 1142.7, 1153.8, 1433.4, 1491.6, 1496.7, 1728.5, 1906.3, 3034.0, 3063.8, 3145.2, 3152.6

B: 11.2343102 2.2520569 1.8980088

C	-1.213416	0.873880	0.000000
H	-1.792750	-0.056259	0.000000
C	0.838713	2.132474	0.000000
H	1.477245	2.105730	0.880454
H	0.243016	3.045305	0.000000
H	1.477245	2.105730	-0.880454
N	0.000000	0.950014	0.000000
O	-0.527988	-2.126069	0.000000
O	0.633421	-1.860022	0.000000

SP-10a

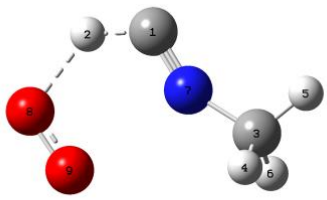

T_1= 0.0388, D_1= 0.1807

$\tilde{\nu}$: -745.2, 74.1, 85.1, 165.5, 276.5, 284.3, 422.6, 768.0, 827.7, 983.8, 1131.0, 1156.8, 1390.8, 1456.3, 1485.3, 1493.9, 2096.2, 2323.8, 3069.7, 3150.3, 3167.4

B: 11.0809720 3.0411666 2.4222699

C	-1.166266	0.723375	0.000000
H	-1.582619	-0.351806	0.000000
C	1.157277	1.732943	0.000000
H	1.748658	1.507065	0.883755
H	0.872662	2.785336	0.000000
H	1.748658	1.507065	-0.883755
N	0.000000	0.906070	0.000000
O	-0.772047	-1.784897	0.000000
O	0.430369	-1.531111	0.000000

$CH_3N{\equiv}C{\bullet}HO_2$

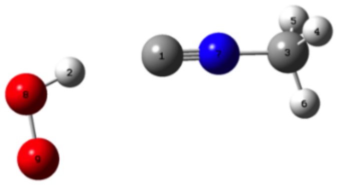

T_1= 0.0273, D_1= 0.1289

$\tilde{\nu}$: 35.7, 37.7, 62.7, 120.6, 220.9, 314.2, 326.3, 635.1, 979.3, 1152.8, 1153.4, 1279.8, 1454.3, 1496.9, 1497.6, 1555.2, 2341.0, 3091.1, 3172.4, 3173.2, 3330.7

B: 23.8157432 1.3499278 1.2878775

C	-0.456442	0.445403	-0.000532
H	1.448093	0.686184	0.000012
C	-2.939932	-0.241839	0.000312
H	-3.409236	0.096676	-0.919679
H	-3.440177	0.208475	0.853659
H	-3.004505	-1.324699	0.067363
N	-1.572484	0.145320	-0.000284
O	2.425962	0.531652	0.000369
O	2.547970	-0.769810	-0.000124

$CH_3N{\equiv}C$

T_1= 0.0141, D_1= 0.0320

$\tilde{\nu}$: 296.1, 296.3, 978.0, 1152.2, 1152.5, 1455.2, 1499.1, 1499.5, 2293.4, 3086.4, 3163.8, 3165.0

B: 159.2117115 10.1401261 10.1401007

N	0.313679	0.000015	0.000013
C	1.475375	-0.000006	-0.000006
C	-1.106295	-0.000008	-0.000004
H	-1.470118	0.516788	0.884734
H	-1.470067	0.507837	-0.889923
H	-1.470039	-1.024646	0.005157

$CH_3NCHO\dot{O}_{syn}$

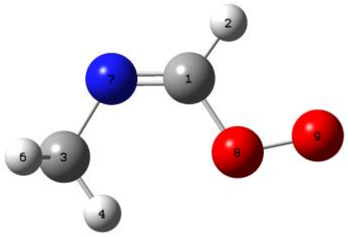

T_1= 0.0287, D_1= 0.1357

$\tilde{\nu}$: 137.5, 144.0, 240.3, 459.2, 462.8, 662.8, 961.5, 985.3, 1035.3, 1146.7, 1175.9, 1279.1, 1354.8, 1444.0, 1506.7, 1508.4, 1816.8, 3073.0, 3143.1, 3153.7, 3213.2

B: 18.6180871 3.0792812 2.6857578

C	0.104620	0.720508	-0.000005
H	0.733590	1.603836	-0.000011
C	-1.889130	-0.532063	0.000003
H	-1.277648	-1.432994	0.000098
H	-2.535140	-0.530956	-0.876537
H	-2.535295	-0.530870	0.876427
N	-1.134822	0.709392	0.000006
O	0.878112	-0.457219	-0.000011
O	2.155052	-0.193459	0.000009

SP-11a

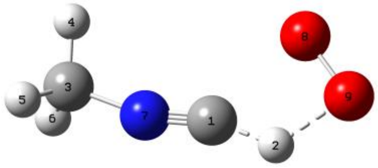

T_1= 0.0218, D_1= 0.0855

$\tilde{\nu}$: -736.6, 68.5, 105.0, 110.0, 291.2, 346.6, 482.4, 905.5, 1049.5, 1102.8, 1147.2, 1159.5, 1319.1, 1453.9, 1494.4, 1498.5, 1883.1, 2404.6, 3086.4, 3167.7, 3171.9

B: 24.3699556 2.0534349 1.9165668

C	-0.054309	-0.514309	0.000002
H	1.031162	-1.013849	0.000001
C	-2.493637	0.245461	0.000000
H	-2.406839	1.329750	0.000124
H	-3.029945	-0.085374	0.885578
H	-3.029849	-0.085176	-0.885711
N	-1.197503	-0.338484	0.000000
O	1.584067	0.771536	0.000001
O	2.304141	-0.291895	-0.000001

SP-sa

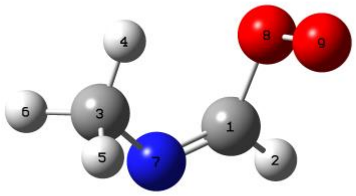

T_1= 0.0287, D_1= 0.1364

$\tilde{\nu}$: -118.2, 112.4, 286.4, 326.8, 522.9, 684.8, 909.2, 965.4, 1013.2, 1139.2, 1170.1, 1242.7, 1360.0, 1438.5, 1494.9, 1506.7, 1814.7, 3071.5, 3143.9, 3152.6, 3204.9

B: 11.1236559 3.9450790 3.2701075

C	0.091367	0.941994	-0.044560
H	0.519107	1.910946	-0.272854
C	-1.599034	-0.689950	0.076832
H	-0.888450	-1.332130	0.595135
H	-1.891787	-1.160585	-0.860681
H	-2.499318	-0.570898	0.676115
N	-1.091666	0.633292	-0.236403
O	1.057290	0.068973	0.543291
O	1.623723	-0.668053	-0.377857

$CH_3N{=}CHO\dot{O}_{anti}$

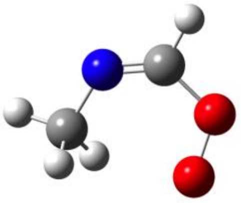

T_1= 0.0146, D_1= 0.0524

$\tilde{\nu}$: 97.4, 198.1, 352.8, 607.6, 622.1, 1022.5, 1056.9, 1155.9, 1181.0, 1312.2, 1404.0, 1472.9, 1492.5, 1497.4, 1543.3, 1822.4, 2993.1, 3063.4, 3121.0, 3148.6, 3624.6

B: 44.9521223 4.4096249 4.1194372

C	-0.183470	1.031747	-0.043970
H	-0.538276	2.053323	-0.123297
C	1.544451	-0.625391	0.074686
H	1.458095	-1.178542	-0.859313
H	1.020437	-1.191600	0.844816
H	2.596049	-0.534517	0.332553
N	1.011047	0.712753	-0.098004
O	-1.317463	0.216374	0.177827
O	-1.154977	-1.038383	-0.139455

SP-11b

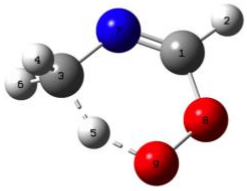

T_1= 0.0325, D_1= 0.1705

$\tilde{\nu}$: -1286.7, 153.3, 312.5, 484.5, 515.1, 629.5, 843.8, 937.3, 977.9, 1061.8, 1134.7, 1165.8, 1210.7, 1262.3, 1387.5, 1474.4, 1667.5, 1759.6, 3094.8, 3170.2, 3222.3

B: 9.1983570 5.5972332 3.5743869

C	-0.173180	-1.069021	0.036287
H	-0.494295	-2.102527	0.070682
C	1.394900	0.636019	-0.041583
H	1.820620	1.030104	0.879231
H	0.286570	1.195035	-0.130335
H	2.003512	0.857150	-0.915212
N	1.028188	-0.718407	0.044952
O	-1.253753	-0.263081	-0.092617
O	-1.014252	1.093968	0.069210

$\dot{C}H_2NCH_2OOH$

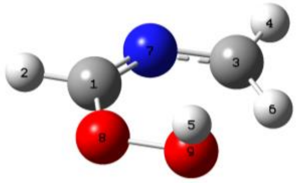

T_1= 0.0222, D_1= 0.0693

$\tilde{\nu}$: 64.1, 111.5, 231.8, 298.6, 361.5, 537.1, 773.3, 792.5, 805.6, 927.6, 1111.9, 1171.5, 1195.3, 1410.0, 1419.1, 1488.2, 1558.8, 3157.1, 3197.8, 3265.4, 3822.6

B: 9.9834435 4.5942091 3.1866187

C	-0.005965	1.023447	0.019065
H	-0.171500	2.094201	0.041410
C	1.603637	-0.717778	-0.049015
H	2.660011	-0.881026	0.104506
H	-1.724373	-1.112432	0.781355
H	0.971084	-1.548218	-0.324133
N	1.172982	0.536151	0.079510
O	-1.240668	0.447117	-0.106179
O	-1.200848	-0.964567	-0.016322

SP-12

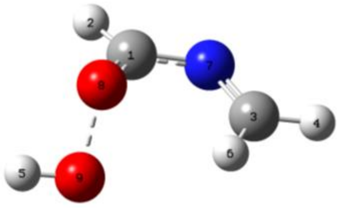

T_1= 0.0207, D_1= 0.0717

$\tilde{\nu}$: -731.5, 55.7, 186.7, 194.4, 328.1, 370.2, 583.9, 734.0, 825.9, 1041.6, 1110.7, 1150.0, 1207.8, 1246.6, 1359.5, 1452.4, 1588.0, 3111.2, 3191.4, 3226.3, 3832.9

B: 10.6444392 4.0643795 3.2945026

C	0.076711	0.948099	-0.096581
H	-0.154087	1.879134	-0.600382
C	1.513949	-0.792106	0.116933
H	2.514565	-1.194682	0.021227
H	-2.441180	-0.347186	-0.318196
H	0.724522	-1.426715	0.513379
N	1.308981	0.425293	-0.255994
O	-0.870345	0.363036	0.592354
O	-1.548485	-0.715981	-0.335627

CH_2NCHO

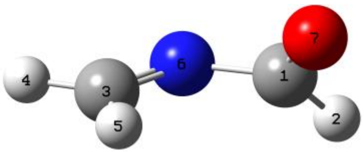

T_1= 0.0163, D_1= 0.0531

$\tilde{\nu}$: 127.9, 386.9, 520.8, 820.1, 1028.5, 1077.6, 1122.2, 1229.0, 1404.2, 1509.3, 1762.6, 1833.1, 3037.9, 3072.9, 3185.9

B: 28.2492551 6.4195238 5.4927889

C	-0.717413	0.420766	0.154189
H	-1.104280	1.297468	0.692244
C	1.432976	-0.351898	0.089119
H	2.469137	-0.283833	-0.228954
H	1.127766	-1.224207	0.670895
N	0.629391	0.568192	-0.222443
O	-1.398967	-0.522497	-0.129617

Table S7. QCC results for $CH_2N\dot{C}H_2 + O_2$.
Energies (/Hartree) of species included in Figure 4 in the main text and energy differences (/kJ mol^{-1}) between stationary points on the PES of the $CH_2NCH_2 + O_2$ reaction. Results from CCSD(T*)-F12a/aug-cc-pVTZ//M06-2X /aug-cc-pVTZ calculations.

	M06-2X/aTZ		CCSD(T*)-F12a/aTZ	
Species	E_{Elec}	E_{ZPE}	E_{Elec}	$\Delta E_{v=0}$
$CH_2N\dot{C}H_2$	-133.275074	0.054060	-133.111712	
2 O_2	-300.649630	0.007994	-300.382273	
Sum reactants	-433.924704	0.062054	-433.493984	0.0
$CH_2NCH_2O\dot{O}$	-283.624301	0.064639	-283.329329	
O_2	-150.324815	0.003997	-150.191136	
Sum	-433.964651	0.070118	-433.535267	-87.2
SP-17	-283.608623	0.060749	-283.314257	
O_2	-150.324815	0.004005	-150.191136	
Sum	-433.933438	0.064754	-433.505394	-22.9
$H\dot{C}NCH_2OOH$	-283.624301	0.064639	-283.3293288	
O_2	-150.324815	0.004005	-150.191136	
Sum	-433.949116	0.068644	-433.520465	-52.3
SP-18b	-283.590341	0.060415	-283.296172	
O_2	-150.324815	0.003997	-150.191136	
Sum	-283.590341	0.060415	-283.296172	23.7
HCN	-93.423992	0.016574	-93.312498	
CH_2O	-75.733810	0.008588	-75.670688	
OH	-114.498970	0.027013	-114.383833	
O_2	-283.656773	0.052175	-283.367019	
Sum products	-433.981588	0.056180	-433.558156	-183.9
$\dot{O}O\bullet HCNCH_2OOH$	-433.952551	0.069614	-433.525028	-61.7
SP-19a	-433.943532	0.068159	-433.520879	-54.6
$HOO\bullet CNCH_2OOH$	-433.994691	0.072451	-433.565953	-161.7
$CNCH_2OOH$	-283.068530	0.054889	-282.768898	
HO_2	-150.908108	0.014608	-150.778909	
Sum products	-433.976637	0.069497	-433.547807	-121.8
$\dot{O}$OCHNCH2OOH	-434.014900	0.074838	-433.585354	-206.3
SP-20a	-433.951467	0.068300	-433.524685	-64.2
SP-20b	-433.983603	0.069732	-433.555800	-142.1

CHONCO	-282.012252	0.033028	-281.708628	
H_2O	-76.430107	0.021551	-76.370982	
OH	-75.733810	0.008588	-75.670688	
Sum products	-434.176169	0.063167	-433.750298	-670.0

Table S7, continued.

T_1 and D_1 diagnostic values, vibrational frequencies (cm^{-1}), Rotational constants (GHz) and Cartesian coordinates (A) of the species listed above.

Species

$CH_2N\dot{C}H_2$ and OH, see Table S3. O_2, see Table S5.

$CH_2NCH_2O\dot{O}$

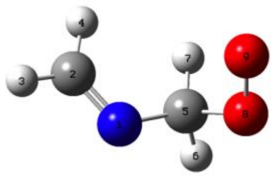

T_1= 0.0283, D_1= 0.1347

$\tilde{\nu}$: 61.2, 131.6, 383.8, 488.8, 554.1, 741.1, 978.1, 1029.9, 1109.1, 1126.5, 1230.1, 1252.3, 1311.4, 1405.9, 1490.3, 1510.9, 1777.5, 3047.1, 3049.6, 3149.2, 3180.9

B: 12.6500000 0.0026411 3.6363211

N	-1.017596	0.256260	-0.491863
C	-1.864838	-0.462060	0.099876
H	-2.686378	-0.888067	-0.468420
H	-1.816731	-0.684250	1.170360
C	0.045360	0.789397	0.312937
H	0.076199	1.872488	0.214997
H	0.009623	0.480763	1.361960
O	1.307441	0.343659	-0.218327
O	1.499725	-0.911006	0.054235

SP-17

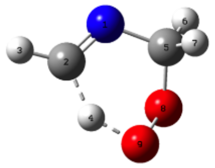

T_1= 0.0245, D_1= 0.1088

$\tilde{\nu}$: -1880.1, 321.5, 374.8, 521.5, 584.1, 674.8, 748.9, 911.1, 994.6, 1099.0, 1102.5, 1133.6, 1174.5, 1288.6, 1366.4, 1479.0, 1679.6, 1830.2, 3083.9, 3128.7, 3175.1

B: 9.3667043 5.6086781 3.8045297

N	-1.215092	0.473799	0.010316
C	-1.298118	-0.752034	-0.090723
H	-2.227071	-1.311307	-0.190004
H	-0.076920	-1.285029	0.006476
C	0.118542	1.065128	0.179767
H	0.167805	2.013513	-0.347087
H	0.285088	1.191715	1.251032
O	1.093969	0.250307	-0.399641
O	1.085305	-0.975814	0.233779

$H\dot{C}NCH_2OOH$

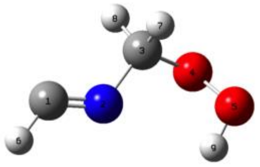

T_1= 0.0202, D_1= 0.0809

$\tilde{\nu}$: 75.3, 162.9, 326.7, 331.6, 414.7, 605.9, 684.0, 916.0, 966.2, 1078.8, 1116.0, 1143.6, 1300.1, 1387.3, 1425.1, 1471.3, 1887.9, 3073.1, 3080.7, 3144.1, 3781.7

B: 15.9960723 3.1677512 2.8142113

C	-2.238512	-0.181729	0.013423
N	-1.027794	-0.281629	-0.094905
C	-0.050155	0.746331	0.241901
O	1.160724	0.484283	-0.395606
O	1.708745	-0.679691	0.199376
H	-2.977883	-0.947800	-0.229520
H	0.083480	0.758060	1.323959
H	-0.366141	1.720490	-0.129749
H	1.231351	-1.383690	-0.262455

SP-18b

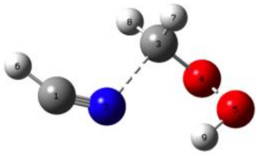

T_1= 0.0205, D_1= 0.0628

$\tilde{\nu}$: -631.7, 60.7, 153.4, 264.6, 333.1, 438.5, 518.5, 611.9, 660.0, 813.7, 957.1, 1001.7, 1188.6, 1235.9, 1448.7, 1462.4, 1946.8, 3123.0, 3255.1, 3316.9, 3728.4

B: 12.9088948 2.9807961 2.5746902

C	-2.317911	-0.243446	-0.021998
N	-1.170489	-0.451970	-0.150700
C	0.189007	0.930482	0.244489
O	1.268326	0.444897	-0.405120
O	1.631790	-0.781590	0.209993
H	-3.164748	0.366572	0.234128
H	0.220636	0.876934	1.326628
H	-0.169179	1.832125	-0.234253
H	0.879214	-1.340506	-0.045537

HCN

T_1= 0.0147, D_1= 0.0297

$\tilde{\nu}$: 785.4, 785.4, 2254.5, 3466.4

B: 0.0000000 45.2323391 45.2323391

N	0.000000	0.000000	0.647246
C	0.000000	0.000000	-0.494926
H	0.000000	0.000000	-1.561168

CH_2O

T_1= 0.0154, D_1= 0.0447

$\tilde{\nu}$: 1213.6, 1273.5, 1539.9, 1869.1, 2945.5, 3015.7

B: 284.7518606 39.4556659 34.6539588

C	0.000000	0.000000	-0.525505
H	0.000000	0.938356	-1.105549
H	0.000000	-0.938356	-1.105549
O	0.000000	0.000000	0.670516

$\dot{O}O\bullet HCNCH_2OOH$

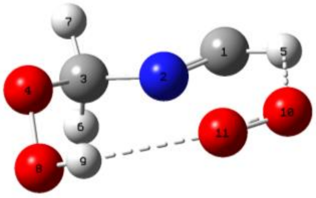

T_1= 0.05210, D_1= 0.3225

$\tilde{\nu}$: 26.9, 49.4, 55.2, 97.1, 108.0, 155.9, 187.4, 336.3, 352.8, 416.1, 610.2, 690.3, 914.8, 965.7, 1072.8, 1106.3, 1141.8, 1298.2, 1385.2, 1432.3, 1469.3, 1734.6, 1899.9, 3051.5, 3079.7, 3145.8, 3773.5

B: 3.6500000 0.0057684 1.5296492

C	-0.549072	1.925334	0.143189
N	0.118080	0.915137	0.027286
C	1.567767	0.790851	0.016050
O	1.934300	-0.447188	-0.506773
H	-1.641903	1.985673	0.154760
H	1.934373	0.899549	1.037148
H	2.015198	1.529228	-0.648336
O	1.518755	-1.435348	0.420790
H	0.580187	-1.532010	0.202002
O	-2.743249	-0.222775	-0.020336
O	-1.938130	-1.092877	-0.130183

SP-19a

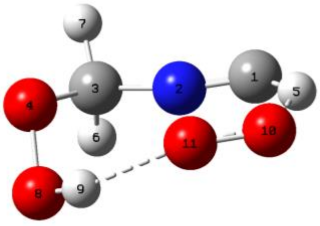

T_1= 0.03485, D_1= 0.19401

$\tilde{\nu}$: -805.1, 52.0, 106.3, 135.9, 167.7, 232.7, 288.9, 395.5, 416.8, 459.3, 603.2, 748.4, 810.9, 938.4, 963.2, 1103.6, 1134.8, 1300.3, 1336.6, 1432.6, 1452.7, 1470.0, 2080.9, 2310.7, 3088.2, 3153.2, 3735.4

B: 4.0200000 0.0048375 1.8418197

C	-0.979911	1.648053	0.224142
N	0.010572	1.032222	0.048055
C	1.438591	0.903069	-0.053603
O	1.788636	-0.356606	-0.516156
H	-2.022065	1.156909	0.207117
H	1.862779	1.092993	0.932277
H	1.810640	1.608601	-0.795327
O	1.476504	-1.282753	0.510084
H	0.539686	-1.465996	0.330876
O	-2.448940	-0.398917	-0.026697
O	-1.443340	-1.077324	-0.221550

HOO•$CNCH_2OOH$

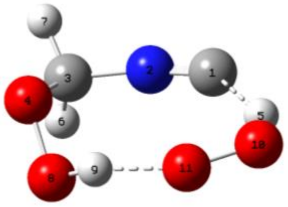

T_1= 0.03485, D_1= 0.19401

$\tilde{\nu}$: 60.2, 77.6, 107.8, 125.1, 186.2, 229.8, 247.3, 308.0, 434.4, 555.2, 596.6, 636.4, 928.3, 963.4, 1122.0, 1147.7, 1304.2, 1314.3, 1404.0, 1483.3, 1491.1, 1543.4, 2306.9, 3103.3, 3170.0, 3310.6, 3645.1

B: 3.9200000 0.0040781 1.5925798

C	-0.327303	1.694655	0.128512
N	0.751630	1.280391	0.069559
C	1.942640	0.478059	-0.004775
O	1.632066	-0.773139	-0.512326
H	-2.103125	0.960114	0.044278
H	2.365080	0.413299	0.996955
H	2.640237	0.932472	-0.704415
O	0.852833	-1.441350	0.464561
H	-0.058944	-1.224272	0.192884
O	-2.727960	0.196795	-0.056894
O	-1.981524	-0.867384	-0.115221

$CNCH_2OOH$

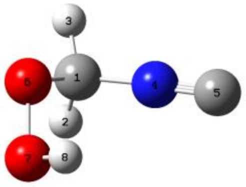

T_1= 0.01398, D_1= 0.03451

$\tilde{\nu}$: 119.2, 205.5, 283.9, 302.3, 428.9, 623.1, 952.6, 962.1, 1115.6, 1141.1, 1318.1, 1409.0, 1425.4, 1483.4, 2265.0, 3102.0, 3163.0, 3793.5

B: 12.5500000 0.0005690 3.7845509

C	-0.053703	0.891324	0.221230
H	0.142382	0.976329	1.288978
H	-0.248611	1.865884	-0.221775
N	-1.194176	0.051354	0.035271
C	-2.095502	-0.670149	-0.111960
O	1.058720	0.391550	-0.450676
O	1.480734	-0.773065	0.237427
H	1.045063	-1.476621	-0.263728

ȮOCHNCH2OOH

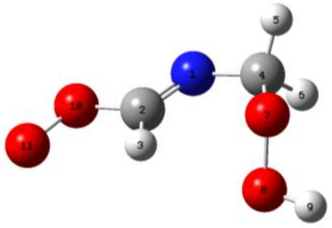

T_1= 0.02527, D_1= 0.13558

$\tilde{\nu}$: 97.4, 129.3, 185.4, 220.0, 242.5, 283.3, 386.3, 467.3, 556.5, 688.8, 935.6, 988.4, 1062.1, 1101.0, 1120.4, 1133.3, 1278.0, 1321.7, 1350.3, 1389.4, 1409.0, 1465.7, 1814.8, 3080.8, 3131.9, 3179.7, 3831.1

B: 6.0500000 0.0023815 1.4261778

N	0.010114	1.097045	-0.230181
C	-0.732222	0.144382	0.055330
H	-0.500734	-0.836580	0.461720
C	1.423945	0.984122	-0.015332
H	1.731768	1.767530	0.677887
H	1.925253	1.125923	-0.973842
O	1.869173	-0.206773	0.589793
O	1.747775	-1.244892	-0.380904
H	2.665339	-1.529934	-0.467224
O	-2.096299	0.319781	-0.169412
O	-2.775994	-0.740276	0.169615

SP-20a

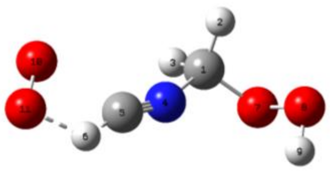

T_1= 0.02193, D_1=0.0866

$\tilde{\nu}$: -1069.5, 36.2, 56.4, 101.9, 143.4, 220.6, 246.7, 335.2, 419.6, 491.5, 614.2, 838.2, 957.7, 989.3, 1104.6, 1132.1, 1146.0, 1314.1, 1318.0, 1404.2, 1420.5, 1471.4, 1816.9, 2323.9, 3101.8, 3165.2, 3810.4

B: 10.3800000 0.0045882 0.8868757

C	1.285244	0.854805	0.086083
H	1.347986	1.006447	1.162677
H	1.148733	1.798132	-0.439543
N	0.152450	0.016691	-0.172492
C	-0.954616	-0.333967	-0.220377
H	-1.990042	-0.967256	-0.398693
O	2.460372	0.311370	-0.413426
O	2.798266	-0.786880	0.419175
H	2.578676	-1.540520	-0.144502
O	-2.736646	0.557221	0.318395
O	-3.289027	-0.524044	-0.094985

SP-20b

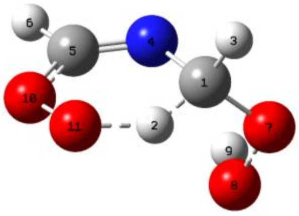

T_1= 0.02527, D_1= 0.13558

$\tilde{\nu}$: -1145.5, 74.4, 130.2, 227.6, 278.3, 374.4, 437.7, 506.9, 522.8, 688.1, 859.6, 948.0, 959.9, 995.6, 1037.6, 1101.1, 1174.1, 1218.9, 1331.1, 1365.6, 1399.2, 1428.3, 1701.6, 1728.7, 3121.7, 3212.5, 3785.2

B: 5.1100000 0.0007886 1.8442604

C	0.571879	-0.317142	0.779422
H	-0.195895	-1.075885	0.174402
H	0.578832	-0.690946	1.803401
N	-0.048228	0.942842	0.621575
C	-1.169485	1.023557	0.052237
H	-1.662255	1.971720	-0.128028
O	1.872404	-0.362152	0.338760
O	1.917985	0.068410	-1.009039
H	1.968796	1.030139	-0.911525
O	-1.894495	0.031624	-0.480277
O	-1.491674	-1.247058	-0.134347

CHONCO

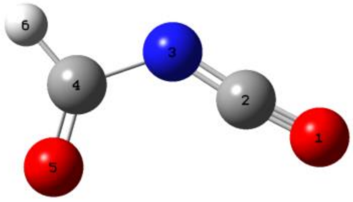

T_1= 0.01829, D_1= 0.0519

$\tilde{\nu}$: 122.9, 168.2, 583.1, 657.5, 771.9, 957.4, 1051.7, 1412.5, 1487.8, 1830.8, 2345.4, 3108.2

B: 20.4400000 0.0073709 3.3979157

O	2.066541	-0.391551	0.000004
C	1.042878	0.132525	-0.000005
N	0.022093	0.797803	-0.000004
C	-1.309369	0.339747	0.000004
O	-1.634081	-0.807514	-0.000002
H	-2.015390	1.174264	0.000016

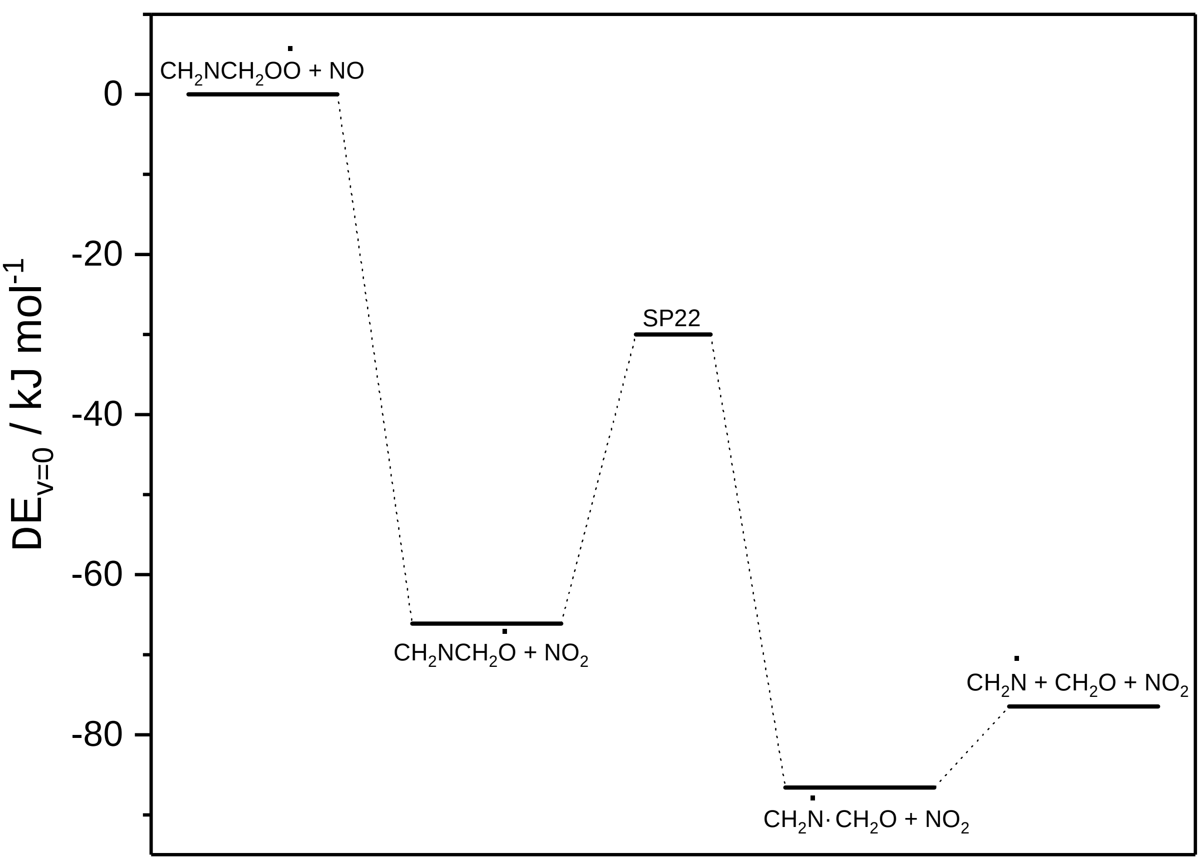

Figure S6. Relative energies of stationary points on the PES of the CH_2=$NCH_2O\dot{O}$ + NO reaction.
Results from CCSD(T*)-F12a/aug-cc-pVTZ//M06-2X/aug-cc-pVTZ calculations.

Table S8. QCC results for $CH_2NCH_2O\dot{O}$ + NO.
Energies (/Hartree) of species included in Figure S6 in the main text and energy differences (/kJ mol^{-1}) between stationary points on the PES of the $CH_2NCH_2O\dot{O}$ + NO reaction. Results from CCSD(T*)-F12a/aug-cc-pVTZ//M06-2X /aug-cc-pVTZ calculations.

	M06-2X/aTZ		CCSD(T*)-F12a/aTZ	
Species	E_{Elec}	E_{ZPE}	E_{Elec}	$\Delta E_{v=0}$
$CH_2NCH_2O\dot{O}$	-283.639836	0.066113	-283.344130	
NO	-129.893351	0.004707	-129.769607	
Sum reactants	-413.533187	0.070820	-413.113737	0.0
$CH_2NCH_2\dot{O}$	-208.479791	0.059932	-208.2484515	
NO_2	-205.074635	0.009168	-204.888741	
Sum products	-413.554426	0.069100	-413.137192	-66.1
SP-20	-208.460012	0.057665	-208.2324172	
NO_2	-205.074635	0.009168	-204.888741	
Sum products	-413.534647	0.066833	-413.121158	-30.0
CH_2N•CH_2O	-208.477150	0.054480	-208.2507993	

NO_2	-205.074635	0.009168	-204.888741	
Sum products	-413.551785	0.063648	-413.139540	-86.6
CH_2N	-93.971161	0.025414	-93.861079	
CH_2O	-114.498970	0.027013	-114.383833	
NO_2	-205.074635	0.009168	-204.888741	
Sum products	-413.544767	0.061594	-413.133653	-76.5

Table S8, continued.

T_1 and D_1 diagnostic values, vibrational frequencies (cm^{-1}), Rotational constants (GHz) and Cartesian coordinates (A) of the species listed above.

Species

$CH_2NCH_2O\dot{O}$ and CH_2O, see Table S9

NO

T_1= 0.0203, D_1= 0.0470

$\tilde{\nu}$: 2066.0

B: 0.0000000 52.3561936 52.3561936

N	0.000000	0.000000	-0.606410
O	0.000000	0.000000	0.530609

$CH_2NCH_2\dot{O}$

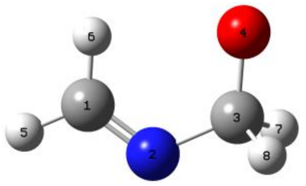

T_1= 0.0208, D_1= 0.0793

$\tilde{\nu}$: 163.6, 346.1, 613.1, 664.0, 775.1, 960.8, 1066.9, 1110.8, 1189.6, 1233.8, 1338.0, 1370.4, 1492.9, 1771.4, 2966.0, 2981.0, 3081.5, 3182.4

B: 20.5631648 6.6890180 5.2062444

C	-1.401418	0.420526	0.000000
N	-0.723968	-0.640296	0.000001
C	0.721250	-0.530964	0.000000
O	1.296237	0.694159	0.000000
H	-2.484896	0.345326	0.000000
H	-0.952575	1.414986	-0.000001
H	1.108179	-1.084441	-0.871063
H	1.108180	-1.084442	0.871061

NO_2

T_1= 0.0251, D_1= 0.0650

$\tilde{\nu}$: 783.5, 1465.2, 1775.4

B: 253.9654019 13.2904639 12.6295376

N	0.000000	0.000000	0.314442
O	0.000000	1.090266	-0.137569
O	0.000000	-1.090266	-0.137569

SP-20

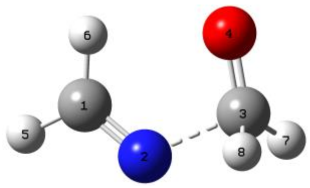

T_1= 0.0355, D_1= 0.1484

$\tilde{\nu}$: -506.6, 88.3, 316.8, 475.9, 560.6, 919.0, 1061.7, 1061.8, 1163.1, 1236.1, 1397.0, 1433.8, 1575.2, 1733.5, 2983.2, 3045.4, 3079.0, 3181.6

B: 19.9937248 6.1974826 4.8874080

C	1.476866	0.358152	0.000125
N	0.766513	-0.667497	-0.000319
C	-1.012080	-0.490358	0.000254
O	-1.178416	0.752313	-0.000234
H	2.556634	0.201969	-0.000062
H	1.085148	1.371294	0.000669
H	-1.183935	-1.062591	0.921466
H	-1.184831	-1.063457	-0.920240

$CH_2N{\bullet}CH_2O$

C	1.687111	0.452871	0.000071
N	1.335274	-0.732941	-0.000183
C	-1.428272	-0.499964	0.000166
O	-1.472187	0.698965	-0.000143

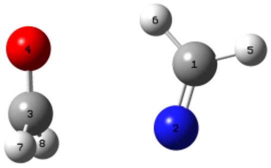

H	2.748285	0.722671	0.000202
H	0.944279	1.258368	0.000171
H	-1.407029	-1.079547	0.936434
H	-1.407986	-1.080068	-0.935804

T_1= 0.0189, D_1= 0.0559
$\tilde{\nu}$: 47.6, 118.1, 146.5, 166.7, 177.7, 195.8, 945.0, 1022.0, 1208.7, 1271.3, 1379.3, 1540.0, 1748.6, 1852.0, 2964.8, 3007.5, 3039.7, 3082.7
B: 18.9442097 3.8031290 3.2389950

CH_2N
T_1= 0.0214, D_1= 0.0543
$\tilde{\nu}$: 939.1, 1005.0, 1380.3, 1752.1, 3005.4, 3073.3
B: 285.4744759 39.7092073 34.8601905

C	0.000000	0.000000	-0.501518
N	0.000000	0.000000	0.735165
H	0.000000	0.937168	-1.068522
H	0.000000	-0.937168	-1.068522

Table S9. G4 results for $CH_3CH{=}CH_2 + O_3$ and $CH_3N{=}CH_2 + O_3$.
Energies (/Hartree) and energy differences (/kJ mol^{-1}) between stationary points on the PES of the $CH_3CH{=}CH_2 + O_3$ and $CH_3N{=}CH_2 + O_3$ reactions.

Species	E_{Elec}	E_{ZPE}	H	G	$\Delta E_{v=0}$
$CH_3CH{=}CH_2$	-117.882260	0.078381	-117.798824	-117.828891	
O_3	-225.370312	0.007353	-225.359058	-225.386725	
Sum reactants	-343.252572	0.085734	-343.157882	-343.215616	0.0
$CH_3CH{=}CH_2{\bullet}O_3$	-343.256123	0.087259	-343.159497	-343.202906	-5.3
SP-24	-343.249039	0.087976	-343.153240	-343.192024	15.2
CH_3-CH-CH_2-OOO (ring)	-343.343750	0.092783	-343.244145	-343.280536	-220.9
$CH_3N{=}CH_2$	-225.370312	0.007353	-225.359058	-225.386725	
O_3	-133.912903	0.067018	-133.841050	-133.870629	
Sum reactants	-359.283215	0.074371	-359.200108	-359.257354	0.0
$CH_3N{=}CH_2{\bullet}O_3$	-359.286822	0.075658	-359.201843	-359.245466	-6.1
SP-25	-359.271361	0.077104	-359.187108	-359.224000	38.3
CH_3-N-CH_2-OOO (ring)	-359.340952	0.080465	-359.253483	-359.289927	-135.6

Table S9, continued.
Vibrational frequencies (cm^{-1}), Rotational constants (GHz) and Cartesian coordinates (A) of the species listed above. Results from B3LYP/6-31G(2df,p) calculations as part of G4.

Species				
$CH_3CH{=}CH_2$	C	1.232863	0.162703	-0.000001
	C	-0.134323	-0.453615	0.000004
$\tilde{\nu}$: 213.1, 420.2, 592.0, 927.8, 943.6, 945.0, 1036.9, 1071.7,	C	-1.280683	0.219288	0.000001
1190.2, 1328.5, 1405.6, 1451.1, 1479.7, 1494.1, 1722.0, 3019.6,	H	1.809570	-0.152117	0.878702
3067.3, 3105.4, 3134.2, 3143.7, 3223.	H	1.180965	1.255339	0.000012
B: 47.0135038, 9.2770399, 8.1364109	H	1.809550	-0.152097	-0.878725
	H	-0.161648	-1.542896	-0.000004
	H	-2.241538	-0.284342	-0.000011
	H	-1.304044	1.305851	-0.000001
O_3	O	1.075741	-0.215378	0.000000
$\tilde{\nu}$: 754.7, 1244.6, 1276.4	O	0.000000	0.431441	0.000000
B: 113.1619375, 13.6518028, 12.1821536	O	-1.075741	-0.216064	0.000000
$CH_3CH{=}CH_2{\bullet}O_3$	O	-1.558376	-0.480025	-0.381997
	O	-1.718200	0.772263	-0.404134
$\tilde{\nu}$: 43.0, 63.8, 109.4, 112.3, 131.8, 226.8, 294.8, 422.4, 601.4,	O	-1.136715	-0.977257	0.700930
753.6, 926.5, 943.8, 949.0, 1013.6, 1063.4, 1191.8, 1214.5,	C	0.878850	1.371464	0.229372
1220.8, 1322.7, 1402.6, 1448.4, 1478.6, 1493.5, 1673.1, 3024.9,	C	1.389363	0.161292	0.493132
3076.4, 3111.0, 3152.0, 3159.9, 3244.4	H	0.424228	1.979416	1.001956
B: 5.3262754, 2.1599460, 1.8253199	H	1.356482	-0.211896	1.514312
	H	0.927150	1.804375	-0.765512
	C	2.050621	-0.728922	-0.514643
	H	3.089244	-0.938264	-0.229896
	H	1.542412	-1.698266	-0.575288
	H	2.053807	-0.278215	-1.511138
SP-24	O	-1.323605	-0.595202	-0.409668
	O	-1.614368	0.649376	-0.275869
$\tilde{\nu}$: -208.1, 83.3, 111.8, 187.2, 216.6, 362.0, 428.6, 478.4, 682.4,	O	-0.735923	-1.084055	0.625697
755.0, 916.0, 945.9, 950.8, 991.5, 1051.5, 1133.4, 1144.8,	C	0.461184	1.403470	0.141430
1192.9, 1299.0, 1401.4, 1440.3, 1478.3, 1491.0, 1589.5, 3023.3,	C	1.123800	0.261167	0.496179
3089.1, 3127.2, 3167.5, 3186.1, 3264.5	H	0.503823	1.782142	-0.873720
B: 5.4990216, 2.8093157, 2.2243837	H	0.032768	2.058299	0.888134
	H	1.227169	0.027382	1.550838
	C	1.938326	-0.554870	-0.463311
	H	2.997740	-0.273623	-0.394885
	H	1.870384	-1.622091	-0.240474
	H	1.619427	-0.391663	-1.496966
CH_3-CH-CH_2-OOO (ring: CH–O)	O	1.389205	-0.747615	0.119530
	O	1.392547	0.584054	-0.428903
	O	0.031325	-1.141485	-0.091072
$\tilde{\nu}$: 73.8, 231.2, 315.5, 372.6, 457.3, 679.3, 725.2, 752.6, 840.3,	C	0.258653	1.164744	0.200332
909.6, 945.7, 958.5, 1009.4, 1084.2, 1144.7, 1167.8, 1233.6,	C	-0.701557	-0.039676	0.449431
1332.3, 1348.0, 1384.6, 1412.5, 1485.3, 1498.5, 1509.1, 3021.2,	H	0.529101	1.652432	1.144012
3037.1, 3046.4, 3104.4, 3120.8, 3128.7	H	-0.831757	-0.180542	1.529806
B: 7.0040178, 3.3852028, 2.5787904	H	-0.147867	1.898968	-0.500584
	C	-2.042437	0.029454	-0.260501
	H	-2.604142	-0.897108	-0.114697
	H	-2.638721	0.853461	0.142672
	H	-1.899179	0.186016	-1.333217

$CH_3N=CH_2$

$\tilde{\nu}$: 223.9, 474.9, 700.0, 970.8, 1062.2, 1125.9, 1147.6, 1242.2, 1436.9, 1477.7, 1479.9, 1511.0, 1752.4, 2971.7, 2988.3, 3068.2, 3087.1, 3132.3

B: 53.4473432, 10.6589162, 9.4018477

C	1.145558	0.134532	0.000043
N	-0.140049	-0.535291	-0.000015
C	-1.179386	0.181649	-0.000050
H	1.715662	-0.185899	0.879276
H	1.076912	1.234368	0.000041
H	1.715739	-0.185898	-0.879139
H	-1.164256	1.282516	-0.000008
H	-2.160753	-0.295138	-0.000020

$CH_3N=CH_2 \bullet O_3$

$\tilde{\nu}$: 45.9, 61.5, 84.9, 99.5, 123.9, 227.1, 246.3, 469.3, 690.3, 752.0, 969.5, 1054.9, 1121.3, 1145.8, 1214.5, 1228.7, 1250.4, 1435.7, 1474.2, 1476.7, 1507.0, 1732.3, 2976.8, 3000.0, 3067.6, 3089.4, 3156.6

B: 5.5105919, 2.1607535, 1.9945950

C	-1.098795	1.228529	0.321221
N	-1.354766	0.012005	0.553760
O	1.277977	-0.638379	0.926845
O	1.537311	-0.462963	-0.289548
O	1.552752	0.728183	-0.714941
H	-1.253543	1.704092	-0.658619
H	-0.707241	1.856631	1.120237
C	-1.836422	-0.811099	-0.535908
H	-1.957825	-0.265390	-1.484816
H	-1.136138	-1.640256	-0.691970
H	-2.794908	-1.258413	-0.251866

SP-25

$\tilde{\nu}$: -332.6, 128.5, 169.5, 246.8, 269.4, 439.6, 508.9, 556.2, 742.9, 806.9, 943.0, 988.7, 1075.1, 1092.6, 1120.0, 1148.5, 1204.8, 1426.0, 1441.2, 1477.4, 1493.5, 1560.2, 3006.7, 3065.8, 3087.8, 3123.0, 3223.1

B: 5.7112957, 3.3295653, 2.8009456

O	-1.142650	-0.642596	-0.386166
O	-1.372314	0.650929	-0.535665
O	-0.700647	-0.903640	0.797517
C	0.390075	1.270248	0.315232
H	0.649147	1.861997	-0.565265
H	-0.146603	1.764647	1.116325
C	1.704781	-0.509624	-0.511038
H	2.536390	-1.091627	-0.108739
H	1.026955	-1.187527	-1.045443
H	2.083057	0.215135	-1.244145
N	1.000971	0.148012	0.573802

CH_3-N-CH_2-OOO

$\tilde{\nu}$: 129.0, 184.0, 254.6, 327.9, 464.4, 540.1, 642.5, 676.4, 766.1, 895.3, 998.6, 1024.0, 1128.8, 1146.2, 1210.1, 1269.2, 1335.5, 1360.4, 1433.6, 1462.9, 1474.9, 1495.8, 3056.7, 3084.4, 3144.4, 3157.6, 3180.1

B: 6.1001488, 3.634015, 3.0851548

O	-1.449086	-0.617916	-0.452142
O	-1.063925	0.679004	-0.499083
O	0.160943	-1.213948	0.465996
C	-0.300959	0.927643	0.686140
N	0.733386	-0.078263	0.493650
H	0.071007	1.950350	0.642529
H	-0.871223	0.685224	1.584967
C	1.727115	0.158161	-0.574555
H	2.149691	1.158116	-0.457886
H	2.513205	-0.587901	-0.454808
H	1.263234	0.050113	-1.558034

Table S10. QCC results for CH_3NCH_2 photolysis.
Energies (/Hartree) and energy differences (/kJ mol^{-1}) between stationary points on the PES of the CH_3NCH_2 dissociation reactions. Results from CCSD(T*)-F12a/aug-cc-pVTZ//M06-2X /aug-cc-pVTZ calculations.

	M06-2X/aTZ		CCSD(T*)-F12a/aTZ	
Species	E_{Elec}	E_{ZPE}	E_{Elec}	$\Delta E_{v=0}$
$CH_3N=CH_2$	-133.922699	0.068777	-133.765118	0.0
SP-26a (*E*)	-133.775100	0.059177	-133.616727	364.4
SP26a (Z)	-133.780891	0.058967	-133.624944	342.3
CH_3NC	-132.711700	0.045649	-132.544189	
H_2	-1.168883	0.010172	-1.174431	
Sum products	-133.880583	0.055820	-133.718620	88.1
SP-26b	-133.765779	0.061651	-133.628400	340.2
CH_4	-40.501737	0.044961	-40.456533	
HCN	-93.424003	0.016612	-93.312535	
Sum products	-133.925740	0.061573	-133.769068	-29.3
$\dot{C}H_3$	-39.825355	0.029787	-39.777335	
$\dot{N}CH_2$	-93.971161	0.025414	-93.861154	
Sum products	-133.796517	0.055201	-133.638489	296.8
(Z)-$CH_3N\dot{C}H$	-133.255130	0.054793	-133.092022	
H	-0.498207	0.000000	-0.499821	
Sum products	-133.255130	0.054793	-133.092022	418.2

Table S10, continued.
T_1 and D_1 diagnostic values, vibrational frequencies (cm^{-1}), Rotational constants (GHz) and Cartesian coordinates (A) of selected species listed above.

Species				
$CH_3N=CH_2$	C	-1.174295	0.182239	0.000000
	H	-1.140148	1.277953	-0.000001
T_1= 0.0182, D_1= 0.0664	H	-2.152857	-0.290246	0.000000
$\tilde{\nu}$: 191.0, 326.9, 393.0, 598.4, 627.1, 982.6, 1044.2, 1140.9,	C	1.143064	0.133755	0.000000
1257.8, 1309.3, 1446.7, 1471.6, 1491.6, 1506.4, 1541.7, 3022.0,	H	1.705616	-0.186179	0.876736
3100.7, 3147.7, 3172.6, 3286.4, 3619.8	H	1.062666	1.226585	-0.000001
B: 42.7480952 9.9050061 8.5808449	H	1.705616	-0.186181	-0.876735
	N	-0.141930	-0.533985	0.000000
SP-26a (*E*)	C	-1.075092	0.347232	-0.000000
	H	-2.216171	0.414084	-0.000000
T_1= 0.0145, D_1= 0.0347	H	-2.175617	-0.598941	0.000000
$\tilde{\nu}$: -1594.1, 183.4, 441.3, 576.2, 650.4, 742.6, 989.2, 1131.8,	C	1.198711	0.114671	-0.000000
1153.6, 1431.8, 1490.6, 1494.9, 1665.3, 1861.6, 2831.6, 3051.8,	H	1.726561	-0.250963	0.879198
3135.6, 3144.2	H	1.186639	1.205993	-0.000001
B: 60.4700000, 0.0061643, 10.0836301	H	1.726561	-0.250964	-0.879198

	N	-0.141383	-0.470090	0.000000
SP26a (Z)	C	-1.231954	-0.103554	-0.000000
	H	-1.130602	1.543089	-0.000001
T_1= 0.0182, D_1= 0.0594	H	-1.986486	0.726458	-0.000001
$\tilde{\nu}$: -1608.3, 205.0, 416.9, 583.4, 790.1, 841.5, 976.0, 1121.5,	C	1.146708	0.176247	0.000000
1141.2, 1251.6, 1447.6, 1480.0, 1493.8, 1936.2, 2921.0, 2995.0,	H	1.717871	-0.107624	0.881653
3127.8, 3155.4	H	0.940276	1.254462	-0.000001
B: 49.4000000, 0.0021248, 10.5456127	H	1.717872	-0.107626	-0.881653
	N	-0.106779	-0.534988	0.000000
SP-26b	C	0.000000	1.094056	-0.000000
	H	1.032787	0.464173	-0.000000
T_1= 0.0291, D_1= 0.1138	H	0.136922	2.173899	-0.000000
$\tilde{\nu}$: -930.8, 410.6, 502.7, 597.9, 681.9, 749.8, 888.2, 999.7,	C	0.674799	-1.342723	-0.000000
1097.3, 1348.9, 1497.7, 1516.0, 1768.9, 1956.8, 3129.6, 3136.1,	H	1.007454	-2.377714	-0.000000
3322.5, 3457.3	H	0.432666	-0.917723	-0.948841
B: 35.6300000, 0.0074616, 8.0769720	H	0.432666	-0.917723	0.948841
	N	-1.013041	0.438156	0.000000

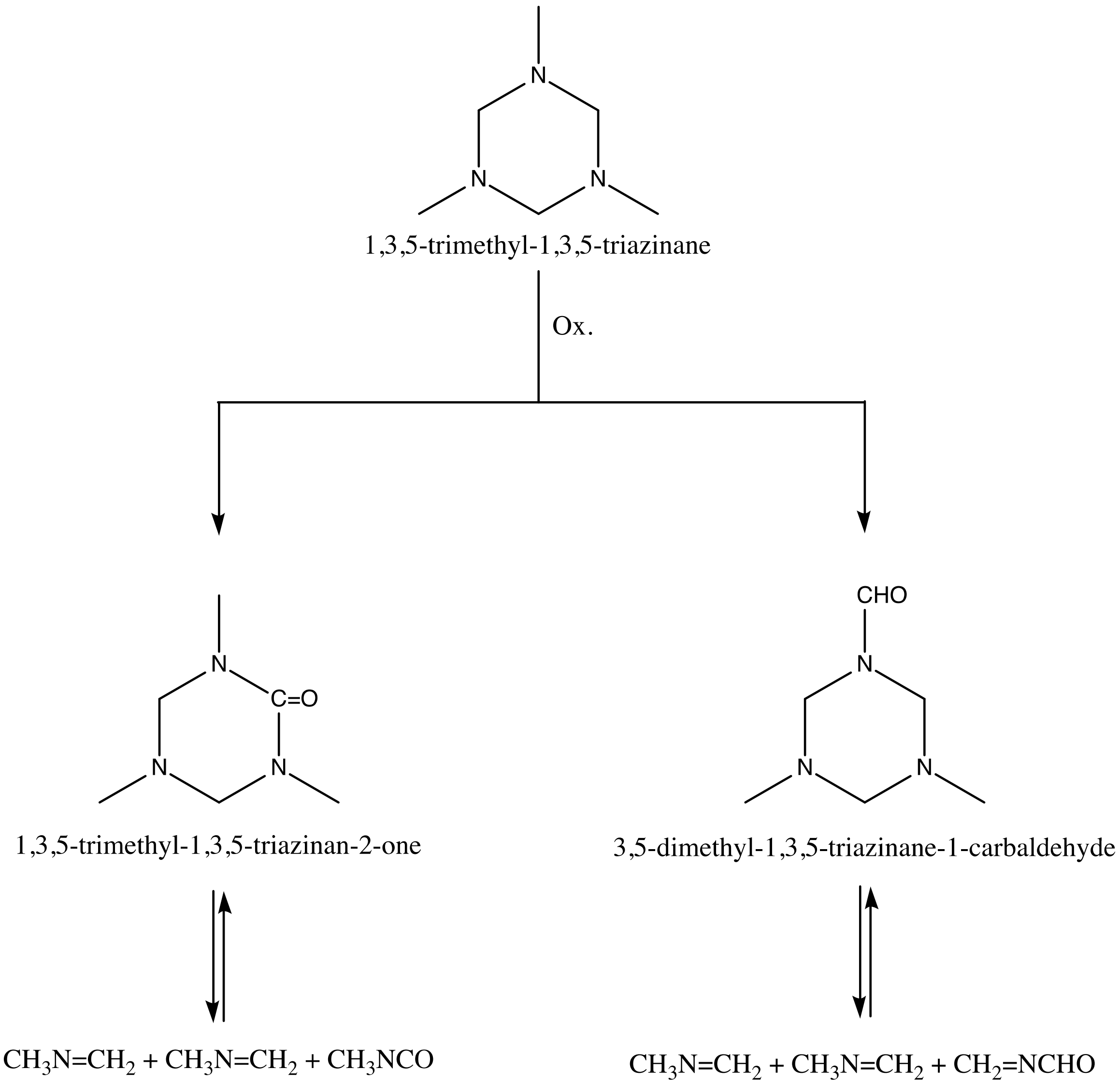

Scheme S1. Primary products in the OH initiated photo-oxidation of TMT under atmospheric conditions

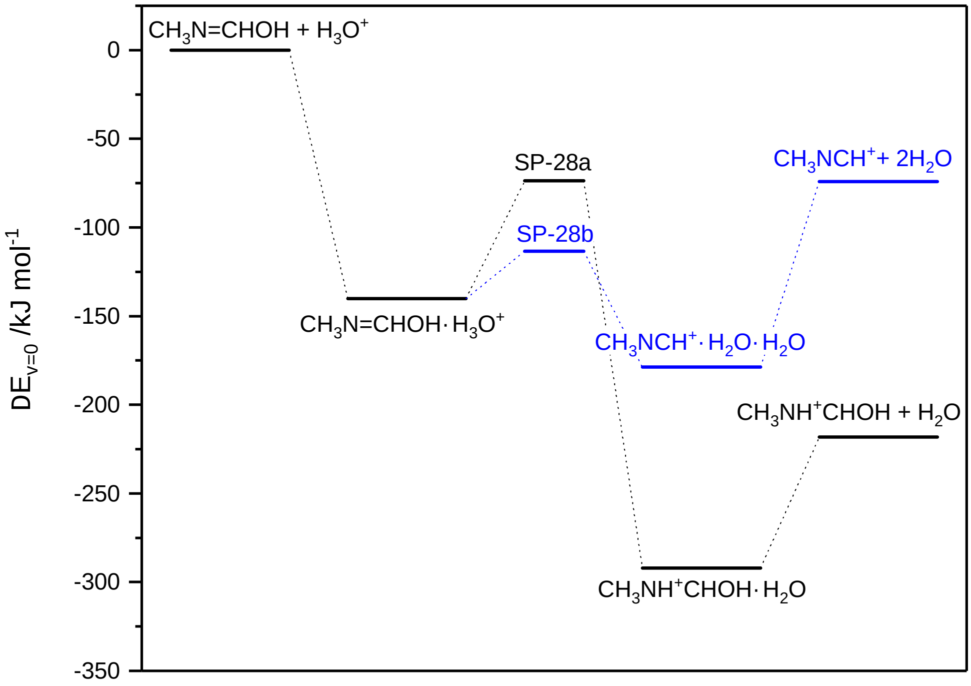

Figure S7. Relative energies of stationary points on the PES of the $CH_3N{=}CCHOH + H_3O^+$ reaction.
Results from CCSD(T*)-F12a/aug-cc-pVTZ//M06-2X/aug-cc-pVTZ calculations.

Table S11. QCC results for $CH_3N{=}CCHOH + H_3O^+$.
Energies (/Hartree) and energy differences (/kJ mol^{-1}) between stationary points on the PES of the $CH_3N{=}CCHOH + H_3O^+$ protonation reaction, Figure S7. Results from CCSD(T*)-F12a/aug-cc-pVTZ//M06-2X /aug-cc-pVTZ calculations.

	M06-2X/aTZ		CCSD(T*)-F12a/aTZ	
Species	E_{Elec}	E_{ZPE}	E_{Elec}	$\Delta E_{v=0}$
H_3O^+	-76.702238	0.034443	-76.644108	
$CH_3N{=}CHOH$	-209.177289	0.074670	-208.949441	
Sum reactants	-285.879526	0.109113	-285.593548	0.0
$CH_3N{=}CHO(H){\bullet}HOH_2^+$	-285.934965	0.108898	-285.646684	-140.1
SP-28a	-285.904280	0.105209	-285.617671	-73.6
$CH_3NH^+CHOH{\bullet}H_2O$	-285.995080	0.113543	-285.709180	-292.0
CH_3NH^+CHOH	-209.532823	0.088540	-209.306685	
H_2O	-76.430107	0.021551	-76.370983	
Sum products	-285.962929	0.110091	-285.677668	-218.3
SP-28b	-285.920969	0.107019	-285.634637	-113.4
$CH_3NCH^+{\bullet}H_2O{\bullet}H_2O$	-285.940478	0.105485	-285.658015	-178.8

CH_3NCH^+	-133.034155	0.057204	-132.871028	
2 H_2O	-152.860213	0.043101	-152.741965	
Sum products	-285.894368	0.100305	-285.612994	-74.2

Table S12. Calculated rate coefficients for the $CH_3N{=}CH_2$ + OH reaction.
Calculated rate coefficients (/10^{-12} cm^3 $molecule^{-1}$ s^{-1}) for the gas phase reaction of $CH_3N{=}CH_2$ with OH radicals. See main text for definition of the routes a – e.

T /K	k_a	k_c	k_d	k_e	k_{tot}
200	7.623	0.334	0.010	0.054	8.021
210	6.599	0.363	0.012	0.053	7.027
220	5.793	0.392	0.013	0.054	6.252
230	5.149	0.420	0.015	0.055	5.640
240	4.628	0.449	0.017	0.057	5.152
250	4.201	0.479	0.019	0.060	4.759
260	3.847	0.510	0.022	0.064	4.442
270	3.551	0.541	0.025	0.068	4.184
280	3.301	0.573	0.028	0.073	3.975
290	3.088	0.607	0.032	0.078	3.804
298	2.940	0.634	0.035	0.083	3.692
300	2.905	0.641	0.036	0.084	3.666
310	2.748	0.677	0.040	0.090	3.555
320	2.611	0.713	0.045	0.097	3.467
330	2.490	0.752	0.051	0.105	3.398
340	2.383	0.792	0.057	0.113	3.346
350	2.285	0.836	0.064	0.122	3.308

Table S13. Rate coefficients for the $CH_3N{=}CH_2$ + OH reaction at discrete values of *p, T*
Units: /10^{-12} cm^3 $molecule^{-1}$ s^{-1}.

T \ *p*	10 mbar	50 mbar	100 mbar	250 mbar	500 mbar	1000 mbar
220	4.96	5.20	5.33	5.60	5.89	6.25
230	4.57	4.81	4.92	5.13	5.37	5.64
240	4.24	4.48	4.58	4.76	4.94	5.15
250	3.96	4.21	4.30	4.45	4.59	4.76
260	3.71	3.98	4.06	4.19	4.31	4.44
270	3.50	3.78	3.86	3.98	4.08	4.18
280	3.31	3.61	3.70	3.80	3.89	3.97
290	3.15	3.47	3.56	3.66	3.73	3.80
298	3.04	3.37	3.46	3.56	3.63	3.69
300	3.01	3.35	3.44	3.53	3.60	3.67
